# Principles of dynamical modularity in biological regulatory networks

**DOI:** 10.1038/srep21957

**Published:** 2016-03-16

**Authors:** Dávid Deritei, William C. Aird, Mária Ercsey-Ravasz, Erzsébet Ravasz Regan

**Affiliations:** 1 Hungarian Physics Institute, Faculty of Physics, Babes¸-Bolyai University, Cluj-Napoca 400084, Romania; 2 Center for Network Science, Central European University, Budapest, 1051, Hungary; 3 Center for Vascular Biology Research, Department of Medicine, Beth Israel Deaconess Medical Center, Harvard Medical School, Boston, MA 02215, USA; 4 Biochemistry and Molecular Biology, The College of Wooster, Wooster, OH 44691, USA

## Abstract

Intractable diseases such as cancer are associated with breakdown in multiple individual functions, which conspire to create unhealthy phenotype-combinations. An important challenge is to decipher how these functions are coordinated in health and disease. We approach this by drawing on dynamical systems theory. We posit that distinct phenotype-combinations are generated by interactions among robust regulatory switches, each in control of a discrete set of phenotypic outcomes. First, we demonstrate the advantage of characterizing multi-switch regulatory systems in terms of their constituent switches by building a multiswitch cell cycle model which points to novel, testable interactions critical for early G2/M commitment to division. Second, we define quantitative measures of *dynamical modularity*, namely that global cell states are discrete combinations of switch-level phenotypes. Finally, we formulate three general principles that govern the way coupled switches coordinate their function.

Approximately every 9 years, the number of new FDA-approved drugs for every billion dollars spent on research and development drops by half^1^. This trend, termed “Eroom’s law”, stands in stark contrast with the rapid progress in computer technology quantified by Moore’s law^2^. Eroom’s law warns that our understanding of complex biological regulation has deep shortcomings, as neither the birth of molecular biology, nor the completion of the human genome project has altered its downward slope. Before the advent of molecular biology, pharmacology relied on phenotypic screening: a direct search of substances that alter a cell’s or organism’s phenotype, with no prior assumptions about their mechanism of action. Newer, molecular target-based approaches aim to disrupt well-mapped molecular pathways known to contribute to cellular pathology. In spite of recent industry preference for this rational approach, more first-in-class drugs were discovered between 1999 and 2008 via phenotypic screening than target-based approaches^3^. In human disease settings, the phenotypic changes expected from target-based drugs often fail to manifest, or trigger additional, unexpected reorganization among seemingly unrelated aspects of a cell’s global phenotype (in the target cell itself or the surrounding tissue), leading to toxicity. A poignant example is anti-angiogenic therapy aimed at solid tumors^4^. While an FDA-approved antibody that blocks a critical growth factor signaling pathway can indeed reduce tumor vessel density and shrink certain primary tumors, the resulting low-oxygen environment can push cancer cells into an invasive, metastatic phenotype^5^.

In general, the most intractable complex diseases affect *more than one* facet of cellular function^6^. Cancer cells, for example, exploit genomic and phenotypic heterogeneity to create therapy-resistant subpopulations and survive in wildly different microenvironments^7^. To do this, they rely on coordinated *phenotype-combinations* drawn from a functional arsenal that includes proliferation, resistance to cell death, new blood vessel recruitment, invasive migration and capillary transmigration^8^. In different combinations and/or contexts, most of these phenotypes are critical to development or health. Indeed, there is extensive evidence that discrete phenotypes co-occur in multiple modular combinations in different conditions or cell types. For example, expression patterns in large microarray-data collections reveal discrete combinations of cellular sub-states^9^,^10^. Furthermore, phenotype disruptions generated by gene knockdown screens cluster into distinct failure modes, in which one of several coordinated phenotypic traits is destroyed, the rest remain untouched^11^,^12^. Deciphering how phenotype coordination goes awry in disease is largely an open problem, in spite of mechanistic understanding of individual functions. We are reasonably well-equipped to build predictive regulatory/signaling models of specific phenotypes such as apoptosis^13^,^14^, proliferation^15^,^16^,^17^ or inflammatory signaling^18^,^19^. What we lack, however, is an understanding (and appropriate modeling frameworks) of how these phenotypes are *coordinated* within single cells into a coherent global response.

Generating distinct phenotype combinations requires a regulatory system that maintains sharp distinctions between discrete cell states. For such sharp distinctions to exist, intermediary states between discrete phenotypes must be transient, and only occur during abrupt transitions between them. They thus require *barriers* between distinct phenotypes, or *multistability* (e.g., survival vs. apoptosis). Within the regulatory system, multistability arises from positive feedback among regulatory molecules (e.g., mutual inhibition between two self-activating transcription factors), often combined with epigenetic modifications which stabilize ON/OFF gene-states^20^,^21^. A growing number of single-cell studies indicate that multi-stable behavior is extremely common. Multistability of circuits responsible for cellular lineage specification was first posited as a theoretical explanation for the existence of discrete cell types with identical genomes^22^. Later experiments identified the bistable switches responsible for several lineage splits (reviewed in^23^,^24^). Even in fully differentiated cell types, bistabile circuits are responsible for irreversible phenotypic changes such as apoptosis^13^ or commitment to division^25^. Upon closer inspection, even cellular phenotypes that we expect to be tunable are often multistabile (e.g., signal-bound receptor endocytosis^26^, the onset of biomass production in bacteria^27^, or cell shape^28^; see Supplementary Note 1).

Embedded into the large, densely wired networks of the cell^29^,^30^, these multi-stable circuits nevertheless maintain a degree of dynamical autonomy as *switches*, in control of regulatory decisions among mutually exclusive phenotypes (Fig. 1A). System-wide cellular interaction networks are known to have hierarchically modular organization; they are composed of nested sub-graphs with high intra-module link densities and sparser inter-module connections at multiple scales^31^,^32^,^33^,^34^,^35^,^36^. Here we posit that the key dynamical building blocks, or *dynamical modules* of the regulatory system are robust regulatory switches. With this premise, we explore the dynamical properties of coupled switch-ensembles responsible for coordinating the combinatorial expression of distinct *switch-phenotype combinations* (Fig. 1B).

## Results

### Two multi-stable switches drive the mammalian cell cycle

The mammalian cell cycle (Fig. 2A)^37^ serves as an excellent case study for the coordination among tightly connected bistable circuits, as the three switches responsible for its abrupt phenotype-transitions are experimentally documented and extensively modeled^38^,^39^,^40^. First, cells entering the cell cycle from a quiescent (G0) state pass a tipping point in late G1, after which they no longer require growth factor stimulation to complete a division. This *restriction point* is a switch-like transition controlled by a bistable molecular regulatory circuit^25^,^41^,^42^. Second, *entry into* mitosis from G2, marked by the sudden disintegration of the nuclear envelope holding the duplicated chromatin, is similarly governed by a bistable switch^43^,^44^,^45^. Third, *exit from* mitosis (the metaphase-anaphase transition) is marked by abrupt clearance of the *Spindle Assembly Checkpoint* (SAC). The SAC prevents sister chromatid separation until every chromosome-pair is attached to the mitotic spindle, ready for synchronized separation. The switch-like transition past the SAC is also controlled by a well characterized bistable switch^46^,^47^,^48^. In fact, the success of modeling these cell cycle switches individually, without the need to use the entire cycle-generating circuit, suggests that this network is dynamically modular.

Using insight from these individual switch-level models, we separated the molecular mediators of restriction point control into our first module, termed the *Restriction Switch* (Fig. 2B, Supplementary Table S1). The network responsible for toggling the cell from G2 into mitosis, then past the spindle assembly checkpoint into cytokinesis (and thus into G0/G1) constitutes our second module, the *Phase Switch* (Fig. 2C, Supplementary Table S2). We then turned to a minimalistic dynamic modeling framework well suited to capture the switch-like function of regulatory circuits, namely Boolean modeling^22^,^49^ (*Methods MBOL1*). Boolean models use the crudest approximation to describe the activity of their regulatory molecules: ON (active) or OFF (inactive). This captures an important qualitative feature of interactions as diverse as biochemical reactions, signaling, cooperative binding or genetic regulation, namely that they respond to changes in input concentration via sigmoid functions with a step-like region of steep change^50^,^51^. In the past decade, Boolean models have emerged as a powerful framework for modeling complex, context-dependent regulatory phenotypes (Supplementary Note 2). This framework allows us to focus on the logic, rather than the kinetic details, of cell cycle regulation.

In contrast to the customary modeling approach of initializing our model with an initial ON/OFF state matched to a biological phenotype and, then investigating its response to a stimulus, here we ask: what is the full dynamic repertoire of our regulatory circuit? What are the states it can stably maintain, and what transient paths does it reach them by? To this end we enumerate every trajectory our model can take from all 2^N^ possible initial states (biological or not), and visualize the resulting *state transition graph* (*Methods MBOL2*). Figure 2D indicates that the *Restrictions Switch* has two locally stable attractor states. One stable state corresponds to cells that have yet to pass the restriction point (labeled *Before Restriction Pt*.), while the other represents mitogen-independent commitment to the next division (*Past Restriction Pt*.). All unstable states of this circuit follow trajectories into one of these two states, partitioning the system’s state space into two attractor basins (green/pink on Fig. 2D). The *Phase Switch*, on the other hand, is tri-stable (Fig. 2E). Its three point-attractors represent i) cells that completed cytokinesis (daughter cells in *G0* or *G1*), ii) cells in *G2* and iii) mitotic cells at the spindle assembly checkpoint (*SAC*). Thus, the pseudo-energy landscape or *attractor landscape* of these switches consists of multiple valleys, separated by barriers of highly unstable states from which the system’s dynamics relaxes back into the valleys (for a quantitative estimate of barrier heights, see *Methods MBOL_noise*, Supplementary Note 3 and Supplementary Fig. S1). These regulatory barriers guarantee that the transitions from *G0/G1* to *G2*, from *G2* into mitosis, and from the *SAC* into anaphase are abrupt and robust.

### The cell cycle switches toggle each other to generate cyclic dynamics

Inside the cell, the two switches are tightly coupled to form the control network of the mammalian cell cycle (Fig. 2F, Supplementary Table S3). To fully account for the processes that influence and link these switches, we introduce four additional nodes: *Growth Factor* (*GF*), *Replication*, *4N DNA* and *Metaphase*. These nodes represent regulatory sub-networks rather than molecular species^19^,^52^ (*Methods FullCC*). The resulting full *Cell Cycle Model* has a limit cycle: the mammalian cell cycle (Fig. 2G, *Supplementarity* Fig. S2), and a fixed-point G0 attractor (Fig. 2H).

The rationale for constructing *individual* models for the two switches, as well as a coupled, modular *Cell Cycle Model*, is that it allows us to evaluate the dynamics of the system in terms of the behavior of its building blocks, the switches. We begin this with visualizing the attractor landscape of a network of *isolated* switches (Fig. 3A; Supplementary Methods 1 details an efficient sampling method, well-suited to identify nearly all attractors even in very large state-spaces). This landscape is composed of every combination of every attractor (phenotype) of each switch. Its state transition graph is thus a set of isolated trees, representing attractor basins of individual switch-phenotype combinations. To visualize this, we use the phenotypes of individual switches as axes of a “coordinate system”, and arrange the combinatorial set of attractor basins on the resulting grid (Fig. 3A, *x* – two *Restriction Switch* states; *y* – three *Phase Switch* states; *z* – two *GF* states; see Supplementary Methods 2). Each switch-phenotype combination is marked with a distinct color. Next, we compare the global attractor landscape of the *coupled* multi-switch network to its disconnected counterpart (Fig. 3B). This landscape is composed of the same ensemble of global network states. The coupled dynamics, however, creates a new set of connections (state-transitions) between them, giving rise to a *different* state transition graph. To visualize this change, we preserved the 3D position of each global network state (nodes on Fig. 3A), but altered its color to reflect the attractor basin it belongs to in the coupled network. This resulted in a color change for all but one basin on the bottom (*No GF* plane of Fig. 3A vs. 3B), indicating that in the absence of growth factors most switch-phenotype combinations are unstable, and converge to a single global *G0* state; namely: the {*Restriction* = *Before RP & Phase* = *G0/G1 & GF* = *OFF*} combination. In contrast, growth factors give rise to a global cycle that toggles through a series of different switch-phenotype combinations. The visual representation thus allows us to “read off” global phenotypes as discrete combinations of switch-level phenotypes.

In addition to visual inference, this process can be automated by matching each global state along an attractor loop to the most similar switch-phenotype combination, and eliminating steps with no switch-phenotype change (Supplementary Fig. S2). This mapping reveals the way the *Restriction* and *Phase Switches* toggle each other’s state (aided by the external nodes) in order to sustain the global cell cycle rhythm (Fig. 3C). Intriguingly, this global cyclic behavior is *not* a property of a single switch, but it emerges from the connections between the underling multi-stable switches. In summary, comparing the dynamics of decoupled switches to the coupled cell-cycle model (Fig. 3A vs. 3B) allows us to step away from the web of biochemical interactions inside the individual switches. Instead, we can directly translate the system’s dynamical attractors into switch-phenotype combinations.

### When do continuously cycling cells pass the restriction point?

Intriguingly, our switch-level view of the cell cycle indicates that the *Restriction Switch* (responsible for commitment to a new cell cycle), re-commits a dividing cell to yet another division *before* cytokinesis is complete (note the {*Restriction* = *Past RP* & *Phase* = *SAC* & *GF* = *ON*} combination). Indeed, direct simulation of mitogen withdrawal by turning OFF the GF node at different time-points along the cell cycle confirms this (Fig. 4A). Our network commits to a new division the moment Cyclin A is deactivated upon mitotic entry (white boxes on Fig. 4A), and completes another full cycle before finally settling into the *G0* state (bottom panel, Fig. 4A). This behavior is surprising, as the canonical understanding of the restriction point posits that it occurs in late G1^53^. Published models of the full mammalian cell cycle reflect this canonical understanding^15^,^16^,^54^,^55^,^56^; they describe cells that become mitogen-independent late in G1, complete a division cycle, reset to an uncommitted early G1 state, and again require growth factors for further division. A recent series of experiments by Spencer *et al*., however, show that a large fraction of continuously cycling human cells actually pass the restriction point before cytokinesis, in late G2/M of the previous cycle (60–75%, depending on cell type)^57^. In fact, about 20% of cells that experience mitogen withdrawal as early as 6 hours *before mitosis* (in G2) not only complete the division in progress, but enter the subsequent cycle. The entire G1 phase of this final cycle lacks environmental mitogens. Closer examination of individual cell states following cytokinesis revealed that continuously cycling cell populations stochastically assume two distinct phenotypes. The majority of cells start with slightly elevated Cdk2 activity, which increases immediately after cytokinesis and leads to rapid S-phase entry. The remaining cells, with slightly lower Cdk2 activity following cytokinesis, exit into a temporary G0-like state where Cdk2 activity remains low. Subsequently, these cells spontaneously reenter the cell cycle after highly variable pause lengths. In fact, the variability of this G0-like interval (experimentally recorded as the M-to-S interval) was found to account for 70% of the cell-cycle length variability. Based on these results, the authors hypothesized that mammalian cells have two distinct restriction points.

In contrast, our model shows that a single bistable switch is responsible for both commitments. Depending on the history of each individual cell, the *Restriction Switch* can either flip its state in late G1 (upon mitogen exposure in G0; Supplementary Fig. S3), or early in Metaphase (in continuously dividing cells; Fig. 4A). Running our model with a stochastic input where *GF* is randomly set to ON with probability *p*_*GF*_ in every time-step allows us to qualitatively reproduce these experimental observations. As Fig. 4B indicates, the random dynamics of our model in an environment with *p*_*GF*_ = 30% exhibits both extremely short cytokinesis-to-S intervals (indicative of commitment during the preceding M phase), and long exits into a G0-like state (blue boxes/bars). The distributions of these time-windows decreases exponentially with window width (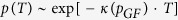 above *T* > 7 time-steps), pointing to a memory-free process with a mitogen-dependent “escape rate” *κ*(*p*_*GF*_) (Fig. 4C). Log-linear fitting of these distributions reveals that the G0 escape rate increases roughly as the 3^rd^ power of *p*_*GF*_ (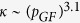; Fig. 4D). The non-exponential transients at low cytokinesis-to-S intervals (*T* ≤ 7) come from cells that have not yet stabilized in G0 before re-committing to another division, allowing us to separate cells into a group that commits to the next division before cytokinesis (blue box on Fig. 4C inset), and another group that linger in G0/G1 longer than the G1-phase of freshly growth-stimulated cells (red box on Fig. 4C inset). The latter fraction declines sharply with growth factor saturation. Experimentally measured values in different cell types are commensurate with low or medium growth stimulation in our model (Fig. 4E).

Prior descriptions of the bistable mechanism responsible for irreversible commitment at the restriction point have focused on the *RB* ┤E2F → Cyclin E ┤RB positive feedback loop^25^,^42^,^58^,^59^,^60^. Once flipped in late G1, this circuit remains committed even in the absence of active Cyclin D (responsible for flipping it by inactivating RB). In G2, however, active CyclinA/Cdk2 complexes block E2F activity (and thus Cyclin E expression). Thus, the above feedback-loop is unable to commit cells to another division. Our model’s ability to reproduce Restriction Point passage in early Metaphase requires additional layers of feedback between E2F → Cyclin D and E2F → Myc, previously modeled only in isolation from the *Phase Switch*^42^ (Supplementary Note 4 and Supplementary Fig. S4). In addition, most published cell-cycle models include an inhibitory link from Cyclin B/Cdk1 to E2F, mirroring the action of Cyclin A/Cdk2^15^,^16^,^17^. The origin of this modeled link is not clear, as its cited source documents inhibitory Cyclin A/E2F1 complex formation, but explicitly shows that Cyclin B does not bind E2F1^61^. We have found no further published evidence for Cyclin B/Cdk1 mediated E2F1 inhibition. Were this interaction added to our model, it would block the Restriction Switch from committing at the G2/M transition, delaying mitogen independence until after the inactivation of Cyclin B/Cdk1 at the start of anaphase (Supplementary Fig. S5).

Based on our 2-switch model, we conclude that the critical event in commitment before cytokinesis is Myc-mediated accumulation of sufficient E2F1 to sustain its own activity, as well as the transcription of Cyclin D and/or Myc following the loss of growth signaling. We expect this to occur abruptly upon Cyclin A deactivation at the start of mitosis. To conclude, our model makes three experimental predictions for mammalian cells capable of restriction point passage in G2/M: 1) There is a Myc threshold at mitotic entry, above which cells make sufficient E2F1 before the loss of growth signals to sustain commitment to another cycle after cytokinesis. The effect of mitogen removal during G2 depends on whether this loss can lower Myc before the transition occurs. 2) Experimental disruption of the E2F1 → Cyclin D1 and/or E2F1 → Myc positive feedback will block the ability of cells to pass the restriction point in G2/M; and 3) Cyclin B/Cdk1 lacks the capacity to inhibit E2F transcription.

### Modular view of the Restriction Point

Modular construction of the Cell Cycle Model allows us to examine the behavior of the *Restriction Switch* as a semi-autonomous circuit (highlighted in Fig. 5A), while treating the rest of the (changing) network as a series of environments within which the *Restriction Switch* operates. To this end, we generate “environmentally constrained” versions of the *Restriction Switch* by freezing each of its external inputs. This procedure reveals that the *Restriction Switch* is only bistable in the absence of growth factors (Fig. 5B). When the *GF* node is ON (and assuming an intracellular environment with the *Phase Switch* in *G0/G1*, as shown on the time-trace on Fig. 5B), the *Restriction Switch* is locked into a single stable state: *Past RP*. This monostability guarantees that upon growth factor stimulation the regulatory network deterministically enters the cell cycle.

More importantly, this method allows us to examine the behavior of the *Restriction Switch* as the cell cycle unfolds (time-trace in Fig. 5C). Under continuous growth stimulation, the *Restriction Switch* is predictably toggled between its *Past RP* and *Before RP* states (Fig. 5C, top). Its behavior is dominated by *Replication* (which resets it into the *Before RP* state), and growth factors, which otherwise keep it in the *Past RP* state. In intracellular environments the *Restriction Switch* encounters upon abrupt growth-factor removal at various points along the cell cycle (Fig. 5C, bottom), the *Restriction Switch* regains its bistability in almost every cell-cycle stage. Exception are S and G2 (when it is locked into *Before RP*), and a brief G2-M transition window (from Cdk1 activation to the loss of Cyclin A activity), when the circuit has a single stable state at the border of *Before RP*/*After RP* (yellow on Fig. 5C). The presence of growth factors between this time-step and the next, where the *Past RP* state is dominant, is critical for early commitment to the next cycle (i.e., exit from cytokinesis with the *Restriction Switch* stably in *Past RP*; *Supplemental* Fig. S6). If growth factors are absent during this window, the switch transitions to *Before RP* at the onset of anaphase (the attractor with a larger basin by this point), and the cell exits into G0.

A module-centric view of the *Restriction Switch* also allows us to explain the non-intuitive effects of the cell cycle inhibitor p21 on restriction point passage. As described in Supplementary Notes 5–6, a p21-positive version of our model reproduces the p21-dependent bifurcation between temporary G0 exit and early cell cycle commitment^13^ (Supplementary Fig. S7), as well as the effect of baseline p21 expression on the stochasticity (heterogeneity) of cell cycle entry^62^ (Supplementary Fig. S8).

### Dynamical Modularity—phenotypes of multi-switch systems are combinations of switch-phenotypes

**Figure 1:**
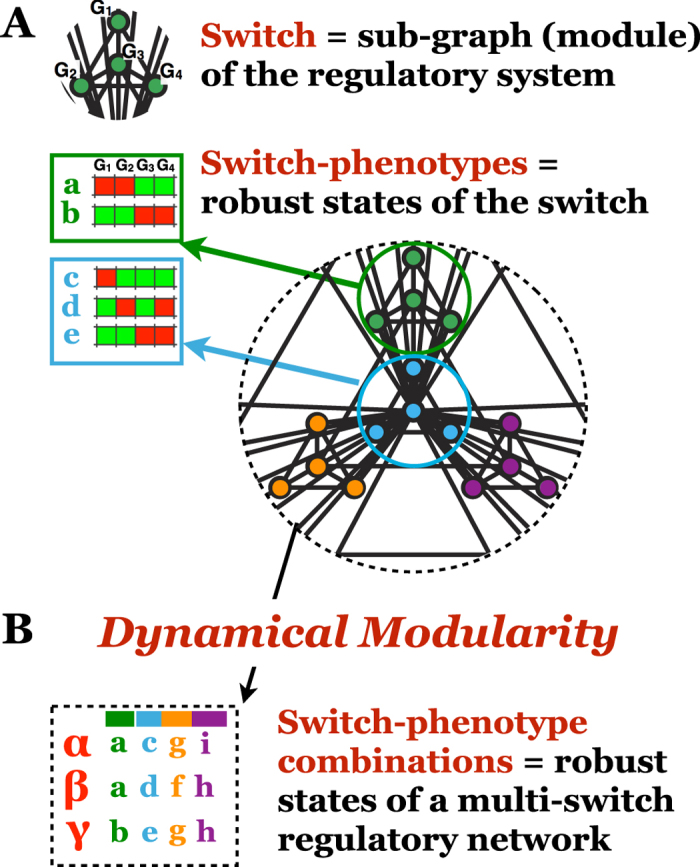
Premise of dynamical modularity. (**A**) A regulatory switch (*top*) is a multi-stable subgraph of the cell-wide regulatory network. In isolation from the network, each switch can maintain multiple mutually exclusive stable states (or rhythmic behaviors), which correspond to discrete cellular functions (*green/blue letters*: phenotypes of the green/blue switch; *red/green squares*: ON/OFF expression/activity patterns of the subgraph in each phenotypic state). (**B**) Dynamical modularity: when coupled to each other to form a modular network, regulatory switches coordinate the expression of distinct switch-phenotype combinations (e.g., in the global state *α*, the green/blue/orange/purple switch expresses its phenotype *a/c/g/i*, respectively).

Our switch-level description of the cell cycle makes the critical assumption that a biological regulatory system’s global phenotypes are combinations of the phenotypes of its switches, a property we call dynamical modularity (Fig. 1). Here we explicitly test this assumption by measuring whether it holds true in biological versus randomized multi-switch networks. To this end we propose an *Attractor Modularity Measure* (*AMM*), designed to quantify the extent to which attractors of a multi-switch system are combinations of the attractors of its switches. To calculate *AMM*, we first map each global phenotype of the multi-switch system onto the most similar combination of individual switch-phenotypes (Fig. 6A; *Supplemental Methods 3*). In a network that conforms to our dynamical modularity premise, we expect all global attractors to fall onto (or toggle through) precise combinations of switch-level phenotypes, resulting in *AMM* = 1 (*Methods* 5). On the other hand, the existence of even one global state that is significantly different from *all* switch-phenotypes caries a severe penalty (Fig. 6B), resulting in low overall *AMM*. In our original p21-null Cell Cycle model 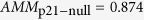, while the p21-dependent model introduced in Supplementary Note 6 has 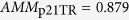, indicating high but not perfect dynamical modularity.

How do these values compare to the *AMM* of random networks? To answer this, we measured *AMM* in three randomized ensembles (Fig. 6C, Supplementary Methods 4): i) random networks with the same node and link number as the Cell Cycle (and input switch-nodes *GF* & *p21TR*); ii) cell cycle models with randomized node-to-switch assignments (preserving the global cell cycle dynamics), and iii) cell cycle switches with rewired inter-switch links (preserving the switches and their internal dynamics). *AMM* was higher in the Cell Cycle Models than in any completely random network. Although it fell in the high range among controls randomized for switch-assignment and inter-switch links, among these it was not an outlier (Fig. 6C, black dashed line).

Closer inspection of randomized systems with high *AMM* values revealed that they occur in two situations (Supplementary Note 7). The first class of random high-*AMM* networks have robust multi-stable switches, but their coupling is either too restrictive (resulting in few global attractor that are near-perfect combinations of switch-phenotypes; Supplementary Fig. S9A), or too loose (resulting in multiple similar switch-phenotype combinations; Supplementary Fig. S9B). In the second class of high-*AMM* random networks, individual switches are composed of loosely connected nodes, leading to several similar switch-phenotypes (Supplementary Fig. S9C). Consequently, global attractor states are typically close to at least one of them. In conclusion, dynamical modularity by itself is not restrictive enough to set apart multi-switch biological networks from a large sub-class of random networks.

### Further Constraints on Dynamical Modularity

As noted above, it is possible to connect robust switches such that the multi-switch system has a single stable switch-phenotype combination. This coupling is so strict that it renders every switch within the regulatory network completely non-functional. The resulting network is strongly homeostatic, but cannot regulate changes in phenotype. By contrast, every phenotype of every switch within our *Cell Cycle Models* plays a biological role in at least one global phenotype. Each switch has some influence on the network’s dynamics, integrating information from its environment and its neighboring switches. To quantify this feature and distinguish the *Cell Cycle Model* from random high-*AMM* networks, we propose the *Switch Stability Measure* (*SSM*). As detailed in *Methods SSM*, *SSM* is built in three layers: measuring the prevalence of each individual switch-phenotype 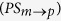 of each switch (*SS*_*m*_) in the dynamics of the multi-switch system (Fig. 7A,B). In our cell cycle models, 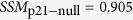 and 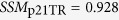. In contrast, *AMM* and *SSM* are rarely *simultaneously* high in random networks (Fig. 7C). Nevertheless, the value-pairs seen in the *Cell Cycle Models* are not unique. Randomized networks with high *AMM* as well as *SSM* are typically made of robust but loosely coupled switches, where nearly every switch-phenotype combination is a different global state (Supplementary Fig. S9B).

In addition to high-*AMM* networks with loose inter-switch coupling, some randomized networks contain switches composed of loosely linked molecules. Clearly, these molecules do not belong to a single biological switch. When studied alone, nodes within these “switches” rarely restrict each other’s state, and give rise to multiple similar switch-phenotypes. In contrast, a robust biological regulatory switch is responsible for a sharp decision between a few distinct outcomes. To quantify this, we posit that attractor states of a robust biological switch are as different from each other as possible, minimizing the chance that noise-induced transitions toggle the switch. We thus define the *Switch-Quality SQ*_*m*_ of switch *m* as the normalized average Hamming distance between each pair of its attractors (0 for mono-stable circuits; see *Methods* 7). To assure that even a single low-quality switch in a dynamically modular network leads to a low network-wide *Switch Quality Measure*, we define *SQM* as a geometric average over the switches: 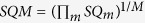.

Using similar logic, we can distinguish higher-level dynamical modules made of tightly coupled switches from those that sit in distant regions of the cell-wide regulatory network and barely influence each other. Loosely coupled switches give rise to systems that generate a variety of similar phenotype combinations (several similar global attractors). In contrast, higher-level dynamical modules govern tightly coordinated phenotype-rearrangements among *all* constituent switches (few, very different global attractors). To track how well the inter-switch links restrict the phenotype-combinations 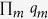 that could, in theory, coexist in a network, we define a *Switch Coordination Measure* 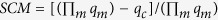, where *q_c_* is the number of global attractors. *SCM* is highest in systems in which *q*_*c*_ is minimal. Combining *SCM*, *SQM*, and the Switch-Quality *SQ*_*c*_ of the coupled system itself, we define the *Switch Quality & Coordination Measure*, 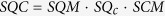. Thus, *SQC* is small in any system that is not made of high quality, highly coordinated switches.

When *AMM*, *SSM* and *SQC* are considered together, none of the random networks (Fig. 7D,E, blue), or randomized molecule-to-switch assignments (Fig. 7D,E, red) give rise to higher values on all three measures than those of the Cell Cycle models (Fig. 7D,E, large black point). We often observe networks that score higher on two measures, but completely fail to satisfy the third (e.g., high *SQC* and *AMM*, but 0 *SSM*; yellow arrows). This gives us confidence that our choice of switches within the Cell Cycle models is optimal. The only random networks capable of outranking the Cell Cycle are very rare instances within the link-randomization ensemble (green points on Fig. 7D; 1 out of 1000 within the p21-null ensemble alone).

### Principles of dynamical modularity in cellular regulation

The three measures proposed above were built to test three specific assumptions. Though their definitions above are specific to Boolean models (Methods 8), the concepts they are based on are generalizable to other modeling frameworks such as continuous or agent-based modeling. Here we argue that these assumptions apply to multi-switch regulatory systems *in general*, and that dynamical modularity is a core property of cellular regulation. We formulate three general principles by which regulatory systems achieve a hierarchical, modular coordination of their phenotypic arsenal.

1. **The Principle of Modular Dynamics** states that *phenotypes of multi-switch regulatory circuits are combinations of switch-phenotypes* (Fig. 8A), quantified in Boolean models by *AMM*. Even though arbitrary connections between switches can easily produce global phenotypes that are *not* combinations of switch-phenotypes, this principle states that biological interactions connecting regulatory switches do not destroy the characteristic of these switch-level cell states. Instead, they influence which switch-phenotypes are selected under different circumstances, without creating new ones. As a result, the essential features of regulatory dynamics can be reduced to switch-phenotype changes. The *coordination* between switches, however, is an emergent property of multi-switch systems. This phenomenon has actually been observed in Boolean regulatory models, and leveraged to reduce computational complexity^63^,^64^,^65^. This principle does not imply that continuously tunable regulatory components (with no multi-stability) are completely absent from cells. It does, however, posit that tunable signal-processing layers feed into core multi-stable circuits. At certain tunable signal thresholds, these circuits transition between discrete phenotypes in a switch-like manner. For example, the strength of growth receptor signaling in single cells may be tunable by growth factor availability, but the subsequent cell cycle entry is an all-or-none decision^39^. Thus, a key role of tunable signaling circuits is to “translate” the environment into internal signals able to flip a switch.
2. **The Principle of Phenotype Conservation** guarantees that each switch has a biological “decision making” function. It states that *every switch-phenotype is present in at least one global phenotype of the multi-switch circuit* (Fig. 8B), quantified in Boolean models by *SSM*. Thus, no switch is unconditionally locked out of any of its robust phenotypic states.
3. **The Principle of Robust Coordination** addresses the hierarchical nature of modularity. It states that *the regulatory system is a hierarchy of Dynamical Modules, each a robust switch with a minimal number of radically different phenotypes* (Fig. 8C), quantified in Boolean models by *SQC*. Thus, dynamical modules at higher levels of the hierarchy, themselves composed of lower-scale switches, act as robust switches between small numbers of complex phenotypes. Within the lowest-scale switches, dense regulatory interactions create a small number of robust phenotypes, collectively expressed by the molecules of a switch. Similarly, inter-switch connections within higher-level (multi-switch) modules severely restrict the number of global phenotypes. Principles 2 and 3, gives rise to an optimization: they require enough phenotype-combinations to accommodate every switch-phenotype (Principle 2), but no more (Principle 3).

## Discussion

Our multi-switch model of the mammalian cell cycle brings some unexpected insights.

First, construction of two separate switches, the *Restriction Switch* and the *Phase Switch*, helped us understand how essential phenotypic transitions of the cell cycle emerge from the impressive mess of interactions that connect its key regulators (86 interactions among 21 molecules, or 4.1 per molecule). Our exploration was aided by a constant back-and-forth between a mechanistic molecule-level description and a higher-level focus on transitions between inter dependent switch-phenotypes. As a result, our model not only reproduces the cyclic molecular cascade of the cell cycle, but reveals the high-level logic by which the two switches bring about this rhythmic sequence of sharp transitions (Figs 3,5 and 6). In the process, we have found that reproducing the experimentally observed order of transitions required changes to previously published models, leading us to the following predictions: 1) Mammalian cells have a critical Myc threshold at mitotic entry, above which they sustain commitment to another cycle in spite of the loss of growth signals during late G2/early Metaphase; 2) disruption of the E2F1 → Cyclin D1 and/or E2F1 → Myc positive feedback will block the ability of cells to pass the restriction point in G2/M; and 3) Cyclin B/Cdk1 does not inhibit E2F1 transcription.

**Figure 2:**
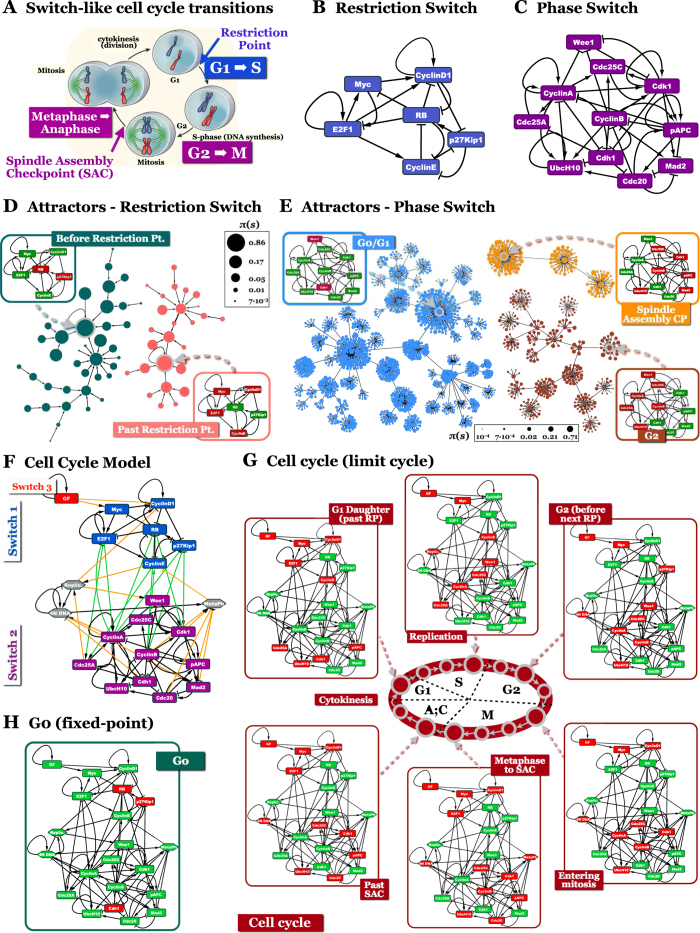
The mammalian cell cycle arises from the coordinated behavior of two modules. (**A**) Overview of the mammalian cell cycle (*labels with colored backgrounds*: abrupt, switch-like transitions along the cycle; *colored text*: pertinent checkpoints). Reprinted by permission from Macmillan Publishers Ltd: *Nature*^37^, copyright (2010). (**B**) *Restriction Switch* in control of restriction point passage. (**C**) *Phase Switch* in control of G2 → M and SAC → G1 passage. (**D**) The *Restriction Switch* is bistable; its 2 attractors represent node activity before (left, green basin) and after (right, pink basin) the restriction point (*Red/green nodes on networks*: ON/OFF). (**E**) The *Phase Switch* is tri-stable; its 3 attractors represent node activity in G0/G1 (left, blue basin), G2 (bottom right, brown basin) and at SAC (top right, orange basin). Node colors on the state transition graphs in (C-D) denote attractor basin membership (*node size*: visitation probability Π(*S*); *p*_GateError_ = 0.02; see *Methods MBOL_noise*). (**F**) Cell Cycle Model composed of the *Restriction Switch*, *Phase Switch*, as well as *Growth Factor*, *Replication*, *4N DNA* and *Metaphase* nodes. (**G,H**) The Cell Cycle Model has a limit cycle representing growth factor-driven cycling (**G**) (detailed in Supplementary Fig. S2), and a fixed-point attractor corresponding to quiescent G0 cells (**H**).

Second, dynamically modular modeling allowed us to examine the effect of changing regulatory environments from the point of view of a single switch. To this end we distinguish between the “free” dynamics of a switch, and its dynamics under specific environmental drive. In building an isolated switch, one ignores all outside regulatory integrations converging on its nodes. This is true of all regulatory model building, and requires the often-unexamined assumption that these outside regulators are either absent or present in a way that allows the model’s internal nodes to maximally influence each other (we formalized this in Supplementary Methods 4.2). Consequently, “free” switches have phenotypes that can be forbidden by certain environments, but may nevertheless be robustly sustained by internal feedback in the *absence* of such a forbidding influence (e.g., free vs. *GF*-driven *Restriction Switch* on Fig. 2A vs. Fig. 5). We argue that in-depth understanding of multi-stable regulatory systems is greatly aided by separate attention to the internal logic of its switches (regulatory feedback loops responsible for the existence of its switch-like behavior), and subsequently, to the effect of certain environments on the state-space of a switch. Applying this to the *Restriction Switch* helps us track cell cycle-dependent changes in the stability of its committed vs. uncommitted states (Fig. 5), pinpointing the moment most cells commit to the next division (early Metaphase), but also the appearance of a robust option to not commit, accessible via biological noise.

Third, our models make the unexpected prediction that it requires far shorter growth stimulation for a cell to commit in early Metaphase than in G0/G1. Indeed, we found that a minimal well-timed *GF* pulse for 1 time-step per cycle can keep the p21-null model continuously cycling (Supplementary Fig. S6). This is even more pronounced in the p21-high model, which can *only* cycle if it commits to the next division at the SAC (it cannot enter from G0! see Supplementary Fig. S7D). An interesting potential application of this prediction is an *in vitro* setup in which cells are pulsed to cycle by a periodically appearing/disappearing growth signal flowing through a microfluidic device. In theory, such a setup could be used to sustain rapid synchronous cycling in a population. An intriguing application may be iPS cell reprogramming, recently shown to be deterministic in mammalian cells that undergo a sequence of several very rapid proliferation cycles^66^.

Guided by the question: *do the two switches of the cell cycle communicate in a way that sets them apart from randomly coupled sub-graphs?*, we proposed three general principles that constrain the coordination of discrete phenotypes. We posited that cellular regulatory networks are composed of a hierarchy of robust multi-stable switches, a hierarchy that obeys three specific principles: dynamical modularity, phenotype conservation and robust coordination of switches that form higher-level dynamical modules. Shown to hold in the network controlling the cell cycle, these principles raise the question: *how do we experimentally prove their general validity?* We expect the rise of single-cell based assays to render the experimental identification of the underlying multi-stable switches increasingly routine. Consequently, it will gradually become feasible to test our proposed principles in a diverse array of systems, even if proving them in general requires an experimentally tested dynamical model of the entire cellular regulatory network, out of reach for the foreseeable future.

In the long run, we expect the strongest support for our principles to come from their utility. If they are true properties of cellular regulation, then building increasingly complete dynamical regulatory models may mandate obeying them, or may be significantly aided by their use. Beyond a certain size, experimental validation of mechanistic dynamical models will necessitate the ability to abstract away from detailed, molecule-level descriptions of regulatory interactions to a larger-scale view of relationships between phenotypes, a need our principles address directly. Dynamically modular networks are amiable to greatly simplified descriptions that capture their dynamics in terms of phenotype-level rules of influence between switches. For example, a dynamically modular model uniting the regulatory switches critical to cell growth, proliferation, apoptosis, migration, inflammation and cell-to-cell communication could provide insight into how breakdown in these core processes leads to cell- and tissue-specific disease. Such a model would benefit drug development in multiple ways. First, it could predict unexpected drug side effects that involve nonlinear changes in the coordination of regulatory processes not directly targeted by the drug. As these models would provide direct phenotype-level description of the cell’s global state in response to a drug, their predictions could be tested in functional as well as molecular assays. Second, dynamically modular models could aid the search for novel interventions designed to bring about particular *global* combinations of cellular phenotypes. Conventional approaches target single phenotypic outcomes such as cell death or inflammation, but restoring or maintaining a desired healthy state may require specific outcome-combinations. Third, fully restoring cells or tissues to health may require more than locking them into a single global state, deemed “optimal”. Indeed, an ideal cure would restore the global dynamical behavior of healthy cells (tissues), along with their full repertoire of responses to changing micro-environmental conditions. Finding such interventions, without accurate dynamical models able to predict the full global phenotype repertoire of cells in response to the intervention, is not possible.

Finally, our work raises a new evolutionary question: why and how did complex regulatory systems acquire dynamical modularity? Studies on evolution of modularity in signal processing circuits offers some insight^67^. Standard genetic algorithms that evolve circuits to compute a single, complex logical function typically produce non-modular circuits, even if the function itself is modular. If, however, the evolving circuit-population is required to adapt, mid-evolution, to goals that retain the modules of this function but combine them in different ways at different times, the best performing systems evolve modules to solve these unchanging sub-tasks. Even the most specialized cell type of a multicellular organism faces micro-environmental changes that require multi-faceted phenotypic responses. At the lowest level, robust switches of the cellular regulatory network may have evolved as solutions to recurrent sub-problems (e.g., is mitogen available long enough to start DNA synthesis?). At higher levels, these discrete regulatory decisions are coordinated to bring about combinatorial (modular) phenotypes best suited to particular cellular environments. Our proposed principles offer a new understanding of the tradeoff between generating robust phenotypes, and the flexibility of responding to environmental demands. Consequently, they may prove valuable as bio-inspired design principles for man-made decision making systems engineered to juggle a hierarchy of choices (e.g., robotic controllers^68^,^69^, group decision making^70^ or logic circuit design^71^).

## Methods

### Boolean Modeling Framework

Throughout this manuscript we model regulatory systems using a Boolean formalism. Building a Boolean model requires knowledge of the regulatory wiring diagram (Fig. 2B,C,F), as well as the logic by which combinatorial regulation occurs. In order to capture this combinatorial logic, Boolean models approximate the concentration/expression of regulatory molecules as ON (active; red regulatory nodes in Fig. 2D,E,G,H) or OFF (inactive; green regulatory nodes in Fig. 2D,E,G,H)^72^. This approximation, although simplistic, captures an important qualitative feature of a broad class of molecular processes: namely their abrupt, sigmoid concentration dependence (seen in biochemical reactions, signaling, cooperative binding, genetic regulation)^50^,^73^,^74^,^75^,^76^,^77^,^78^. The combinatorial influence of multiple regulators converging on a node is encoded by Boolean functions (gates) that characterize its response to the state of its regulators (Supplementary Tables S1–S3).

In a Boolean representation, cells may have 2^*N*^ possible states or “expression profiles” (represented by dark green/pink nodes on Fig. 2D and light blue/brown/orange colored nodes on Fig. 2E), where *N* represents the number of regulatory molecules. All but a very small fraction of these 2^*N*^ states, however, fail to satisfy the Boolean rules of any given model. Dynamics from these states proceed in discrete time steps, in which all nodes receiving inputs that require a different ON/OFF state than their current one change. This change, in turn, can lead to a further cascade of inconsistencies between input and output in downstream nodes. This cascade typically ends in a steady state, in which all Boolean rules are satisfied (*fixed point attractor*, Fig. 2D,E). Alternatively, the system may converge onto a series of states that repeat in a cycle (*limit cycle attractor*, Fig. 2G and Supplementary Fig. S2).

### The state space of a Boolean regulatory network

The collection of all sequential state-changes can be represented as a network of states: the *state transition graph*, connected by arrows depicting the natural dynamics of the system (Fig. 2D,E). The subset of states which lead to a single attractor form its *attractor basin*. Conceptually, the attractor basins of a regulatory network can be thought of as a valley pseudo-energy landscape or *attractor landscape*, separated by regulatory barriers (Supplementary Figs S1A,B). Visualized in 3D, the *x*–*y* coordinates of this energy landscape denote distinct states, while *z* corresponds to the pseudo-energy of each state. For continuous systems with two independent internal state variables, this landscape may be directly calculated^79^. For high-dimensional systems and Boolean models, however, state transition graphs offer a useful alternative for visualizing the energy landscape. Attractors of Boolean regulatory models correspond to phenotypes driven by these circuits^80^,^81^. These phenotypes are expected to be robust against small perturbations, a feature that translates to stability of their respective attractors against random node flips, or noise. Complete immunity against all possible flip combinations, however, is not possible for circuits that have more than one attractor basin, and consequently, regulate a choice among several functional states (phenotypes).

### Biological noise and the regulatory barriers of Boolean switches

Multiple, mutually exclusive phenotypes (e.g., survival vs. programmed cell death) are governed by multi-stable circuits, or switches. In these switches, biological noise can occasionally lead to transitions between attractor basins. Thus, in the presence of noise the network’s dynamics explores the connections and barriers between individual attractor basins^82^,^83^. Consequently, assuming that a small amount of noise affects each logic gate in each time-step offers an elegant way to estimate the long-term probability that the system spontaneously visits *any state* (not just the attractors). It also mitigates some of the drawbacks of synchronous node update^84^,^85^,^86^. In this manuscript we follow the method of Zhang *et al*.^87^, who used a Markov chain approach to calculate the stationary probability 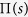 of finding the ergodic system in any state *s* (not just the attractors). To this end, we first calculate the probability matrix *M*_*ij*_ of every state transition *s*_*i *_→ *s*_*j*_ the system can have in a single time-step. Given a nonzero probability *p*_*E*_ that any gate returns the wrong output in every time-step, the system’s dynamics is a Markov process (i.e., all *M*_*ij*_ transitions take place with a nonzero probability). From each state *s*_*i*_ (each row of *M*_*ij*_) there will be a single transition with probability (1 − *p*_*E*_)^*N*^ corresponding to the deterministic, synchronous state transition observed in the noise-free system. In addition, there will be *N* transitions with probability *p*_*E*_(1 − *p*_*E*_)^*N*−1^ where only one of the *N* gates was affected by error, *N*_2_ further transitions with probability 
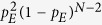
 with two simultaneous gate errors, and so on.

**Figure 3:**
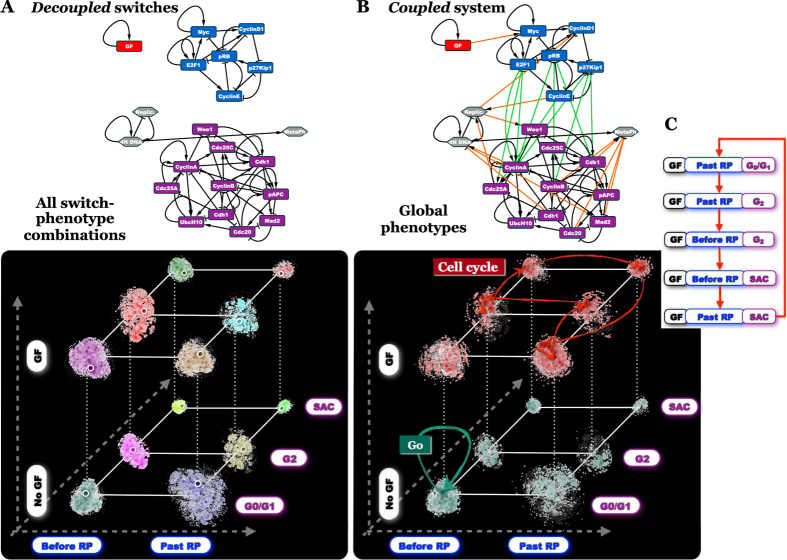
Comparing the dynamics the Cell Cycle network to that of its decoupled switches reveals the sequence of discrete switch-level phenotypes as they follow each other during division. (**A**) Decoupled, the *Restriction Switch* (top, blue), *Phase Switch (top, purple)*, and *Growth Factor switch* (top, red) create a dynamical system with 12 fixed-point attractors, representing all switch-phenotype combinations. The 12 attractor-basins are laid out on a 3D grid, where switch-level attractors are organized along individual axes (*Restriction Switch*: *x*-axis, blue labels; *Phase Switch*: *y*-axis, purple labels; *Growth Factors*: *z*-axis, black labels; *node colors of the state transition graph (STG)*: attractor basin membership; *node size & color saturation*: visitation probability with *p*_GateError_ = 0.02). (**B**) Coupled, the three switches give rise to the Cell Cycle Model (top graph). This coupled system’s STG is represented using the same 3D state-node position as in (**A**), with each state-node re-colored (and resized) to indicate its attractor membership dictated by the dynamics of the coupled network (we omitted most STG links from this figure for clarity; see Supplementary Fig. S10). This procedure reveals a single basin on the *No Growth Factors* plane with the fixed-point attractor G0 (dark green basin) and another basin on the *Growth Factors* plane with a periodic attractor, the cell cycle (red arrows and basin). Sampling of the state transition graphs (*p*_GateError_ = 0.02), generation of individual basin layouts and their arrangement along a 3D module-phenotype grid are described in Supplementary Methods 1, 2.

**Figure 4:**
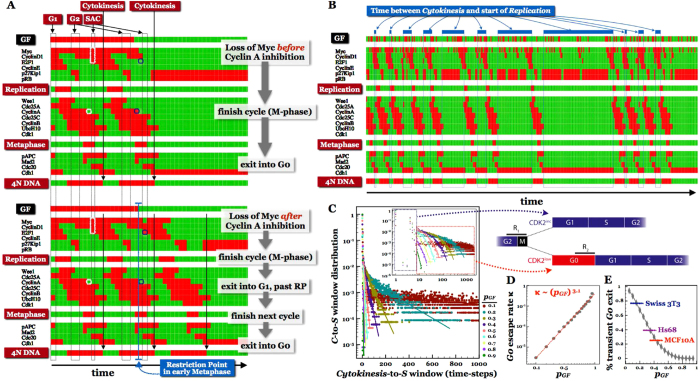
Continuously cycling cells pass the Restriction Point in early metaphase and exhibit stochastic cell cycle exit/entry to/from G0. (**A**) *Top*, time-series of Cell Cycle Model’s dynamics. (*Rows*: regulatory molecules; *columns*: time-steps; *red/green*: cell-wide ON/OFF activity and/or expression of each regulatory molecule as a function of time; *p*_GateError_ = 0). *GF* withdrawal at the G2/M boundary *before* Cyclin A inhibition results in immediate cycle exit following cytokinesis. *Bottom*, GF withdrawal in early Metaphase *after* Cyclin A inhibition leads to commitment to an additional cycle. (**B**) Stochastic cell cycle exit and entry driven by a randomly fluctuating *GF* input with *p*_*GF*_ = 30%. (*Blue boxes/bands*: duration of G0/G1 between subsequent divisions, from Cytokinesis to the next S phase). (**C**) Distribution of the C-to-S window *T* as a function of *p*_*GF*_. Straight lines are exponential fits of the *p*(*T*) distribution, *p*(*T*) ~ exp[−*κ*(*p*_*GF*_) · *T*] for *T* > 7. (7 represents the number of time-steps required to finish cytokinesis and reach G0 if the restriction point is not cleared in early M). *Inset*: log-log scale *p*(*T*) distributions, along with our assignment of cells into those that pass the restriction point in early Metaphase (*blue box*: *T* ≤ 7), and those that transiently exit into G0 (*red box*: *T* > 7), as illustrated in the schematic above panels (**D**,**E**). Reprinted from Cell 155/2, S. L. Spencer et al, The Proliferation-Quiescence Decision Is Controlled by a Bifurcation in CDK2 Activity at Mitotic Exit, 369–383, Copyright (2013), with permission from Elsevier. (**D**) Escape rate *κ* from the transient G0 state as a function of growth stimulation, *p*_*GF*_, revealing the logarithmic dependency *κ* ~ (*p*_*GF*_)^3.1^. (**E**) Percentage of cells that transiently exit the cell cycle as a function of growth stimulation. Horizontal lines mark experimentally reported percentages in three mammalian cell lines^57^.

**Figure 5:**
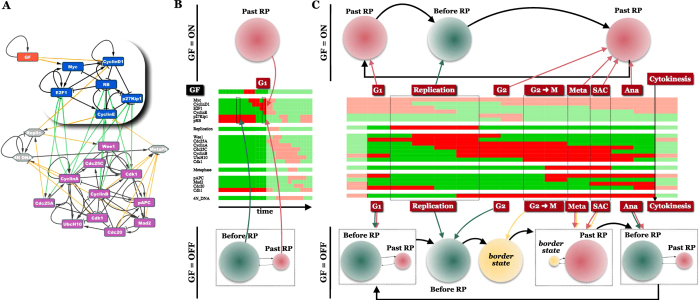
For the Restriction Switch, the cell cycle is a changing environment that modulates the stability of its phenotypes. (**A**) *Restriction Switch* embedded in the Cell Cycle network. (**B**) Attractor basins of the *Restriction Switch* when its internal dynamics are constrained by constant inputs from a *Phase Switch* in *G0*, and a *GF* node locked ON (*top*) or OFF (*bottom*). *Middle*: dynamics of the full model exposed to a short *GF* pulse that triggers a single cycle. (*Green/red arrows*: *Restriction Switch* phenotypes corresponding to the *Before RP*/*After RP* basins; *basin area*: visitation probability; *p*_GateError_ = 0.02). (**C**) Cell cycle phase-dependent attractor basins of the *Restriction Switch*, when its internal dynamics are constrained by a changing *Phase Switch*, as well as a *GF* node locked ON (*top*) or OFF (*bottom*). *Middle:* deterministic cell cycle dynamics of the full model. Arrows map phases of the cell cycle to attractors of the *Restriction Switch*, altered by the changing constraints (*green/red*: *Before RP/After RP*; *yellow*: a border state halfway between *Before RP* and *After RP*).

In stationary state, the overall probability of the system transitioning into state *s*_*i*_ must be balanced by the probability of it leaving *s*_*i*_:


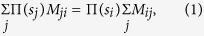


where 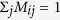. In matrix form: 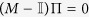 (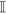 is the identity matrix), an underdetermined system of 2^*N*^ linear equations. Adding the additional constraint that the stationary probabilities across the *entire* state space add up to 1, 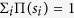, renders the system of equations determined with a single solution. The exact calculation, however, is only feasible on very small Boolean networks (it’s a system of 2^*N*^ + 1 equations). For larger networks we estimate 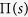 as the visitation probability of state *s*, sampled by long runs of noisy dynamics (see Supplementary Methods 1). The size of state transition graph nodes on Figs 2 and 3, as well as their color saturation on Fig. 3, are proportional to 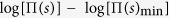. Using 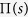, an energy-like quantity associated with each network state during noisy Boolean dynamics can be defined as: 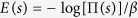, where 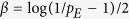 is a function of gate error probability *p*_*E*_^87^.

At a birds-eye view, the stability of an individual attractor (and thus, biological phenotype) may be characterized as the overall probability of finding the system within its basin of attraction:


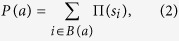


where *s*_*i*_ is an initial condition from which the system’s noise-free dynamics leads to attractor *a*. *B*(*a*), the basin of *a*, denotes the set of all such states. Using a similar logic, we can compute the overall probability of spontaneous transitions between attractor basins as:


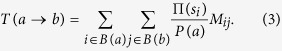


These transition probabilities can be used to gauge the robustness of a multi-stable switch, as detailed in Supplementary Note 1.

### Cellular processes that couple the two cell cycle control switches

To account for the processes that link the *Restriction* and *Phase Switches*, we introduced four additional nodes representing large cellular events or regulatory sub-networks outside our current modeling scope. First, a bistable (self-activating) *Growth Factor* node (*GF*) represents the environment and subsequent signaling that couples growth receptors to AKT and MAPK, culminating in a decision to activate the *Myc* transcription factor. Second, the *Replication* node stands in for the entire process of DNA replication. Replication is turned ON by cyclin E and Cdc25A activation (Cdk2 activation is implicitly assumed when cyclin E and Cdc25A are active), and turned OFF by completion of DNA synthesis. This completion is tracked by the third node, denoted *4N DNA*, turned ON by active *Replication* plus *cyclin A* activation (required for the completion of DNA synthesis), and self-sustaining until cytokinesis restores 2N DNA content and turns it OFF (in our model this is marked by activation of the *APC*^*Cdh*1^ complex). Fourth, the *Metaphase* node represents spindle assembly, turning ON after the cell enters mitosis, and OFF after it clears the spindle assembly checkpoint.

### Defining the Attractor Modularity Measure *AMM*

**Figure 6:**
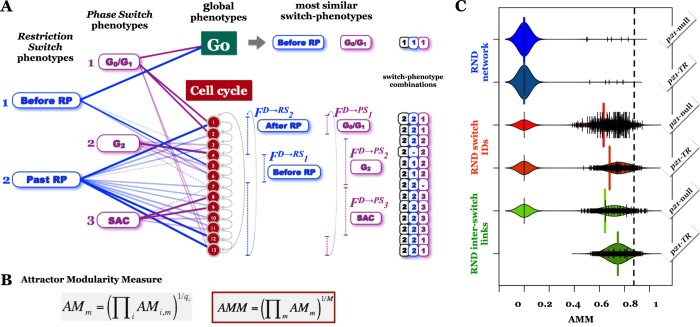
Quantifying dynamical modularity via the Attractor Modularity Measure. (**A**) Translating a sequence of global states into a series of switch-phenotype transitions. (*Blue/purple labels*: phenotypes of the *Restriction/Phase Switch*; *green box*: G0 state of the global dynamics; *red nodes*: sequence of global states corresponding to the global cell cycle phenotype; *blue/purple dashed vertical lines*: segments of the *Restriction/Phase Switch* that map onto the same switch-phenotype *j*, denoted 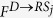 and 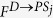; *saturated lines*: global states that fall closest to each switch-phenotype combination along a 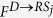 or 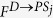 sequence; *unsaturated lines*: global states that fall onto, but *not* closest to a switch-phenotype; *dashed lines*: global states that are equidistant from two switch-phenotypes; (**B**) *Attractor Modularity Measure AMM*, defined as the geometric mean of the attractor modularity values *AM*_*m*_ of individual switches. *AM*_*m*_, in turn, is the geometric mean of the modularity of individual global attractors, as detailed in *Methods AMM*. (**C**) Bean-plots of the *AMM* distribution in 1000 random networks created with complete (*blue*), switch-assignment (*red*) and inter-switch link & gate (*green*) randomization (Supplementary Methods 4; *small black ticks*: *AMM* in individual random networks; *colored background*: estimated density distribution; *colored vertical lines*: average *AMM*). *Black dashed line*: *AMM*_p21−null_/*AMM*_p21TR_ of the Cell Cycle Models.

The first step towards defining the *AMM* measure is to map every global phenotype of the multi-switch system onto the most similar combination of individual switch-phenotypes (Fig. 6A). To this end, similarity is defined as the normalized overlap between each phenotype of an isolated switch *m*, and the ON/OFF state of its constituent molecules in the global attractor state (Supplemental Fig. S11). By extension, we map cyclic global phenotypes or open state-sequences onto a matched series of switch-phenotype combinations (Cell Cycle on Fig. 6A). In this case, the overlap of a global limit cycle with a phenotype of switch *m* is defined by the highest overlap the cycle achieves (namely, the closest the global rhythmic behavior of the system gets to a particular switch-level phenotype; fully saturated lines on Fig. 6A). For a precise definition of overlap between an *arbitrary* global state-sequence and arbitrary switch-phenotypes, including cyclic switch-behaviors, see Supplementary Methods 3 and Supplementary Figs S11–S15).

In order to claim that a global phenotype is a switch-phenotype combination, this global phenotype has to have high overlap with the attractor states of every switch. If the global phenotype is a cyclic behavior, we expect it to either avoid the basin of certain switch-phenotypes altogether, or implement them precisely at some point along the cyclic trajectory. We quantify this via the *Attractor Modularity AM*_*i,m*_ of global phenotype *i* with respect to switch *m*. *AM*_*i,m*_ is defined as:


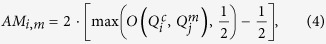


where 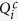 represents the *i*^th^ global attractor (phenotype), and 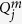 represents the 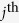 attractor of switch *m*. Their overlap, 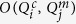, is based on similarity of switch-node expression states between 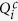 and 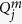, generalized to cover comparisons between arbitrary global and switch-level limit cycles, as described in Supplementary Methods 3.

*AM*_*i,m*_ severely penalizes global states that are significantly different from all switch-phenotypes of *m*. Thus, its lowest value 0 is reached when the overlap between 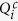 and 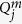 is 1/2, representing a global attractor *i* in which the switch *m* is poised halfway between two completely different phenotypes: the opposite of dynamical modularity. The 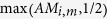 is necessarily because of the way we defined overlap between limit cycle switch-phenotypes and global cycles in Supplementary Methods 3.2. If, for example, a global cycle executes the steps of a switch limit cycle with relatively high pairwise state-overlap but in a scrambled order, it is possible for 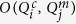 to be nonzero but below 1/2. We consider these situations far from dynamically modular, and treat them as worst-case scenarios with *AM*_*i,m*_ = 0.

The attractor modularity of the entire coupled system with respect to the switch *m* is defined as:


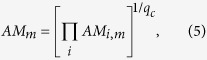


where *q*_*c*_ is the number of global (coupled) attractors. Thus, *AM*_*m*_ is constructed to be very low if *any* of the global attractors are significantly different from all phenotypes of switch *m*.

Lastly, we define the global *Attractor Modularity Measure* as the geometric mean of attractor modularity across the switches, 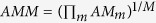 (*M* = number of switches). High *AMM* requires simultaneously high *AM*_*m*_ for every switch.

### Defining the Switch Stability Measure *SSM*

**Figure 7:**
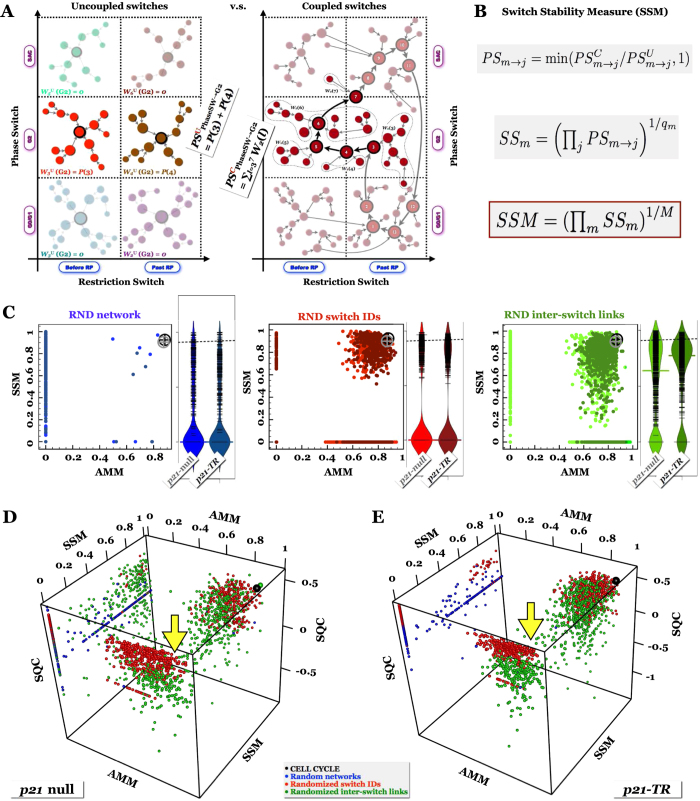
Together, the three dynamical modularity measures distinguish modular Cell Cycle Models from their randomized counterparts. (**A**) Measuring *SSM* by comparing the overall visitation probability of a particular switch phenotype (e.g., G2 of the *Phase Switch*) in the system of decoupled switches to its visitation probability by the coupled multi-switch model (*p*_GateError_ = 0.02). *Left*: schematic attractor landscape of the decoupled *Restriction* and *Phase Switches* with *GF* = ON. (*Saturated nodes*: combinatorial attractor basins in which the *Phase Switch* is in G2; *black border*: attractor states; *no border*: unstable states; *transparent nodes*: attractor basins in which the *Phase Switch* is not in G2). *Right*: schematic attractor basin of the Cell Cycle attractor (*GF* = ON) in the model with coupled switches. (*Saturated nodes*: attractor states [black border] along the cell cycle during which the *Phase Switch* is closest to its G2 phenotype, along with unstable states [no border] of the basin that flow onto the cycle through this G2-associated section; *transparent nodes*: attractor/unstable states that do not map onto the G2 of the Phase Switch. (**B**) *SSM* is defined in three steps (*Methods SSM*): i) *Phenotype Stability PS*_*m*→*p*_ is defined based on the above comparison; ii) *Switch Stability SS*_*m*_ is the geometric mean of *PS*_*m*→*p*_ across the phenotypes of switch *m*, and iii) *SSM* is the geometric mean of *SS*_*m*_ across the switches. (**C**) Scatter plot of *AMM* and *SSM* in randomized networks. (*Black/grey circle with cross*: *SSM*_p21−null_ and *SSM*_p21TR_). (**D,E**) Scatter plot of *AMM*, *SSM* and *SQC* in randomized networks matched to the p21-null (**D**) and p21-TR (**E**) Cell Cycle Models. (*Black*: Cell Cycle; *blue*: random networks; *red*: randomized node-to-switch assignments; *green*: randomized links connecting the two cell cycle switches; *large green dot*: 1 of 1000 random networks that outperform the p21-null Cell Cycle on all three measures; *yellow arrow*: random systems with high *AMM* and *SQC*, but 0 *SSM*).

**Figure 8:**
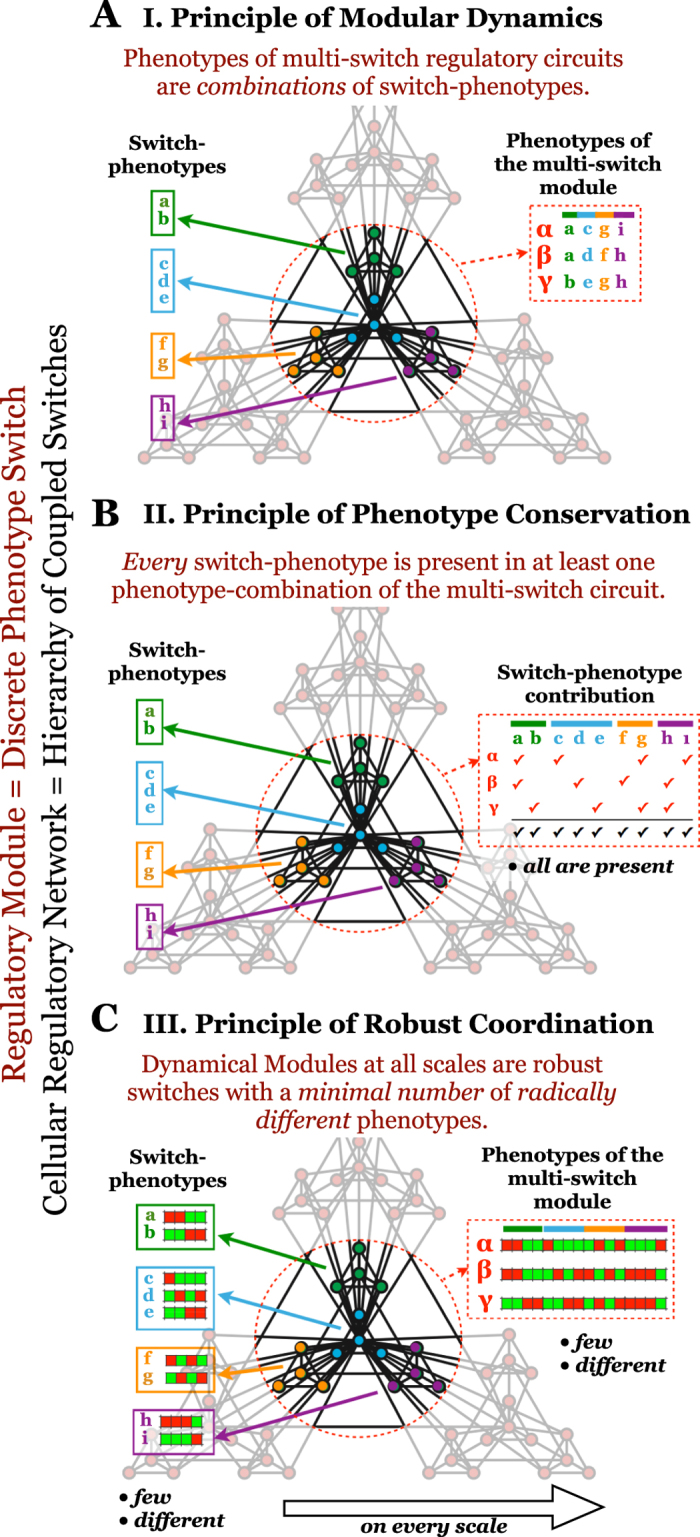
General principles of dynamical modularity in cellular regulation. (**A**) *Principle of Modular Dynamics.* Colored sub-graphs denote circuits that act as individual switches (dynamical modules). Each switch regulates a decision among a few discreet phenotypes (its attractors), labeled by colored letters. The red dashed circle highlights a higher-level dynamical module, composed of several lower-scale switches. Each phenotype of the higher-level, multi-switch module is a combination of the phenotypes of its constituent switches, as illustrated by their labels. (**B**) *Principle of Phenotype Conservation.* Every phenotype of every lower-scale switch (*top right row*) is present in at least one global phenotype (*red checkmark*: membership in a global phenotype; *black checkmark*: membership in at least one global phenotype). (**C**) *Principle of Robust Coordination.* Each switch on every scale has only a small number of very different phenotypic states, as illustrated by differences among the expression/activity pattern of single-switch (*left*) and multi-switch (*right*) phenotypes (*red/green*: ON/OFF expression/activity).

In order to claim that the coupled system’s dynamics replicates a switch-phenotype, two conditions have to be met. First, there needs to be at least one coupled attractor that maps onto this switch-attractor. We test this while calculating *AMM*, in that we compute the overlap 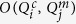 between every coupled attractor *i* and switch-*m* attractor *j*. Second, it is important that even if a coupled 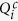 attractor exists that overlaps with switch-*m* attractor *j*, its basin (and thus the overall stability of this global phenotype) not be overly small. In order to quantify this, we first employ noisy Boolean dynamics to calculate (or sample) the long-term probability 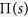 of finding the coupled system in any state *s* in the presence of gate error *p*_*E*_ (see *Methods MBOL_noise*). Next, we estimate the size of the state space region (in terms of visitation probability) from which the system’s dynamics flows into each individual attractor state (Fig. 7A). For fixed-point attractors, this equals the overall probability of finding the system somewhere in their basin. To generalize it to limit-cycles, we map each state *s* of the coupled system onto individual attractor states 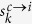 by starting the coupled system in initial state *s*, and updating it in the absence of noise until an attractor state of *i* is reached for the first time: 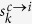, the *k*^th^ state of attractor *i*. For each coupled attractor state 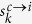 we then sum up the probability 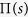 of all states that map to 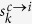: 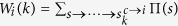. As we wish to approximate the overall probability of finding the coupled system in a state that maps onto switch-*m* attractor *j*, we go through each coupled attractor *i* for which 
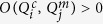
 and sum up the *W*_*i*_(*k*) probabilities along the (full) flyby segment 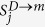 mapped into switch-*m* attractor *j* (for definition of 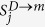, see Supplementary Methods 3). Stated differently, we sum all *W*_*i*_(*k*) for all rows *k* of the mapping matrix 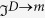 (Supplementary Figs S13 and S14) for which there are nonzero elements in *any* of the columns that correspond to switch-*m* attractor *j*:


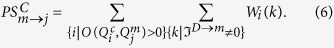


Lastly, we do not expect this overall probability 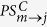 to be larger than the stability of phenotype *j* in the isolated switch *m*. Consequently, we compute a similar overall probability 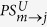
for the composite system made of all *M* switches, but in which none of the inter-switch links are present. The final value of 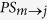 is then computed as


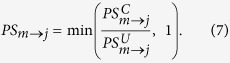


### Normalizing the *Switch Quality* measure

We posit that in a *robust* phenotype switch, the attractor states are as different from each other as possible (thus minimizing the chance that random, noise-induced node state flips can toggle the switch). We can measure this distance using the normalized Hamming distance between attractors. In general, this can be defined as the average normalized Hamming distance between every state pair along the two attractors:


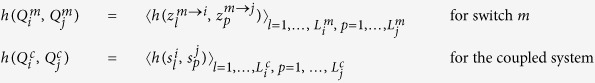


Next, we define the *Switch Quality Measure*, *SQ*, as the ratio between the average of 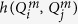 across all attractor pairs of the switch, and the *maximum average* Hamming distance the same number of point attractors could have, in theory:


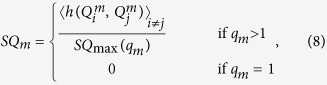


where *q*_*m*_ is the number of switch-*m* attractors.

The *maximum* average normalized Hamming distance *q* number of *N*-node point-attractors is:


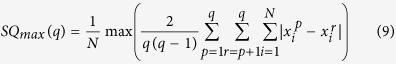



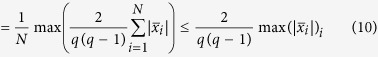


where 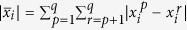 (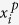 is the value of node *i* in point attractor *p*). In essence, 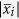 represents the number of bit-pairs with *different* values inside the binary array 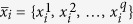. Assuming that *n*_0_ elements in this array have value 0 and *n*_1_ elements have value 1 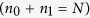, 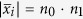. The maximum value of 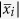 is achieved when half of the array has value 0 and half has value 1. For example, in a binary array with 6 elements (6 attractors), the maximum pairwise difference between bits occurs when there are 3 elements with value 0 and 3 with value 1, in which case 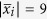. In general:


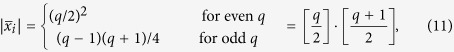


where [*x*] denotes the integer part of *x*. Thus, an upper bound for the average normalized Hamming distance can be written as:


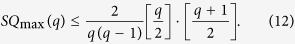


This maximum cannot always be reached with *q distinct* point attractors (especially if *q* is large), as there may not be *q* distinct combinations of 0’s and 1’s in which half of the values are 0. Nevertheless, when *q* is small (typical in our modular systems) this maximum can, in theory, be achieved. Using the method above, we calculate the value of *SQ*_*m*_ for each switch, as well as for the coupled system (*SQ*_*c*_).

### Requirements of measuring *AMM*, *SSM* and *SQC* in arbitrary Boolean networks

To summarize, calculating *AMM*, *SSM* and *SQC* in arbitrary Boolean networks with arbitrary switch-assignments requires:

- (1) a method to cut the network into individual switches by automatically generating reduced Boolean rules that dictate the *internal* dynamics of an arbitrary subgraph (detailed in Supplementary Methods 4.2);
- (2) a full list of attractors for each switch, obtained via exact enumeration when the size of the network permits, the sampling method described in Supplementary Methods 1 otherwise;
- (3) a full list of attractors for the full coupled system of switches via the sampling method in Supplementary Methods 1;
- (4) the steady state visitation probability of states and attractor basins in the coupled as well as uncoupled system of switches (also generated via the sampling method in Supplementary Methods 1).

## Additional Information

**How to cite this article**: Deritei, D. *et al*. Principles of dynamical modularity in biological regulatory networks. *Sci. Rep*. **6**, 21957; doi: 10.1038/srep21957 (2016).

## Supplementary Material

Supplementary Information
