# Supplementary material for: Principles of dynamical modularity in biological regulatory networks
Source: Sci Rep. 2016 Mar 16;6:21957. doi: 10.1038/srep21957 (PMC4793241; doi:10.1038/srep21957)
Supplement: Supplementary Information [file srep21957-s1.pdf]

Principles of dynamical modularity in biological regulatory networks
– *Supplemental Information* –

Dávid Deritei[1,2], William C. Aird[3], Mária Ercsey-Ravasz[1], Erzsébet Ravasz Regan[*,3,4]

[1]Hungarian Physics Institute, Faculty of Physics, Babeş-Bolyai University, Cluj-Napoca 400084, Romania
[2]Center for Network Science, Central European University, Budapest, 1051, Hungary
[3]Center for Vascular Biology Research, Department of Medicine, Beth Israel Deaconess Medical Center, Harvard Medical School, Boston, MA 02215, USA
[4]Biochemistry and Molecular Biology, The College of Wooster, Wooster, OH 44691, USA

[*]Corresponding author (e-mail: eregan@wooster.edu)

SUPPLEMENTARY NOTES

1. Multistability and abrupt switching in seemingly tunable biological processes

Upon closer inspection, even cellular phenotypes that we expect to be tunable are often multistabile. For example, after a general class of C2 domain-containing receptor signaling mediators translocate to the plasma membrane (in response to stimulus), their behavior bifurcates: in a subset of cells they remain at the surface, in the rest they all reenter the cytoplasm [1]. Another example comes from bacteria, where the decision to start division is preceded by another switch-like transition during lag phase: a sudden onset of biomass production or cell growth [2]. This is triggered by the accumulation of proteins that transport and process the available energy source above a critical threshold.

Surprisingly, even the shape of individual cells spread in 2D was found to be multi-stable, clustered in discrete shape-space regions [3]. Cells transitioned between shapes in rapid, stochastic flips. Moreover, knockdown of most genes influenced the distribution of cells among wild-type states, while creation of novel shapes was surprisingly rare.

2. Successes of Boolean modeling in biology

Small multi-stable circuits have been extensively studied in biochemistry and genetic regulation [4, 5, 6]. A common theme emerging from these studies is that regulatory circuits show a remarkable lack of sensitivity to internal parameters, and to modeling details in general [7, 8, 9, 10, 11, 12]. These studies raised the possibility that the essential features of biological interactions are adequately described by simple response functions with discrete values, such as Boolean logic functions [13]. Boolean models have indeed shown remarkable faithfulness to cellular dynamical processes and phenotypes [4, 14, 15] (e.g., cell cycle [16, 17, 18], segment polarity [8, 19], abscisic acid signaling [20, 21], apoptosis [22, 23, 24, 25], floral morphogenesis [26], differentiation [27]). From a theoretical perspective, theorems on sequential dynamical systems offer proof that discrete representations of continuous nonlinear systems can capture all salient features of their dynamics [28].

3. Regulatory barriers separating the stable states of cell cycle control switches

In order to compare the robustness of the *Restriction* and *Phase Switches*, we compared the transition probability across the *weakest* barrier in each switch (i.e., largest $T(a \to b)$; ; see *Methods 3*) to those measured in ensembles of matched, randomized networks (same number of nodes, links and Boolean gate bias). These randomized controls were chosen from a subset of all random Boolean networks using the following procedure:

- Generate a random directed graph with N nodes and K links.
- Rewire the network until all N nodes are part of a single strongly connected component (every node is reachable from every other node via a directed path):
- Generate random Boolean gates for each node, using a probability p_0 for a 0 output ($1 - p_0$ for output of 1), matching the p_0 measured in the biological circuit used for the comparison.
- Guarantee that all inputs of each Boolean gate are functional. While there exists an input i to a Boolean gate that does not alter its output for *(*any) combination of its other inputs, pick a random combination of all inputs except i, and flips the output of the gate for $s_i = 0$ or 1, chosen randomly.
- Check whether the final network has the same number of attractors as the biological circuit used for comparison. If not, discard network.

Compared to random Boolean networks with a matched number of point-attractors, the *Restriction Switch* has a very high barrier between its two phenotypes (**Fig. S1C**, two-tailed Mann-Whitney U test: $p_{\text{RS}} \simeq 0.001$). The *Phase Switch* performs slightly better than an average matched random Boolean network, but not significantly so (**Fig. S1D**, $p_{\text{PS}} \simeq 0.44$), and it is average among networks with similar basin-size distributions. This is largely due to its weak barrier between *SAC* and *G0/G1*. We suspect that the high biological transition barrier between these states leverages the tension-based stabilizing effect of unattached kinetochores, external to the modeled *Phase Switch*. Interestingly, even its noise-driven state-transitions have an overall preference for a *G2* $\to$ *SAC* $\to$ *G0/G1* flow (i.e., toggling in the biologically relevant direction; see arrow thickness on **Fig. S1D**), while strongly resisting the opposite (i.e., turning back the cell cycle clock). One exception is the *G2* $\to$ *G0/G1* flip, the barrier of which is the second lowest. In cells, this transition can actually occur under prolonged *G2* arrest, which leads to loss of cyclins and mitotic bypass to a G0-like senescent phenotype with 4N DNA content [29].

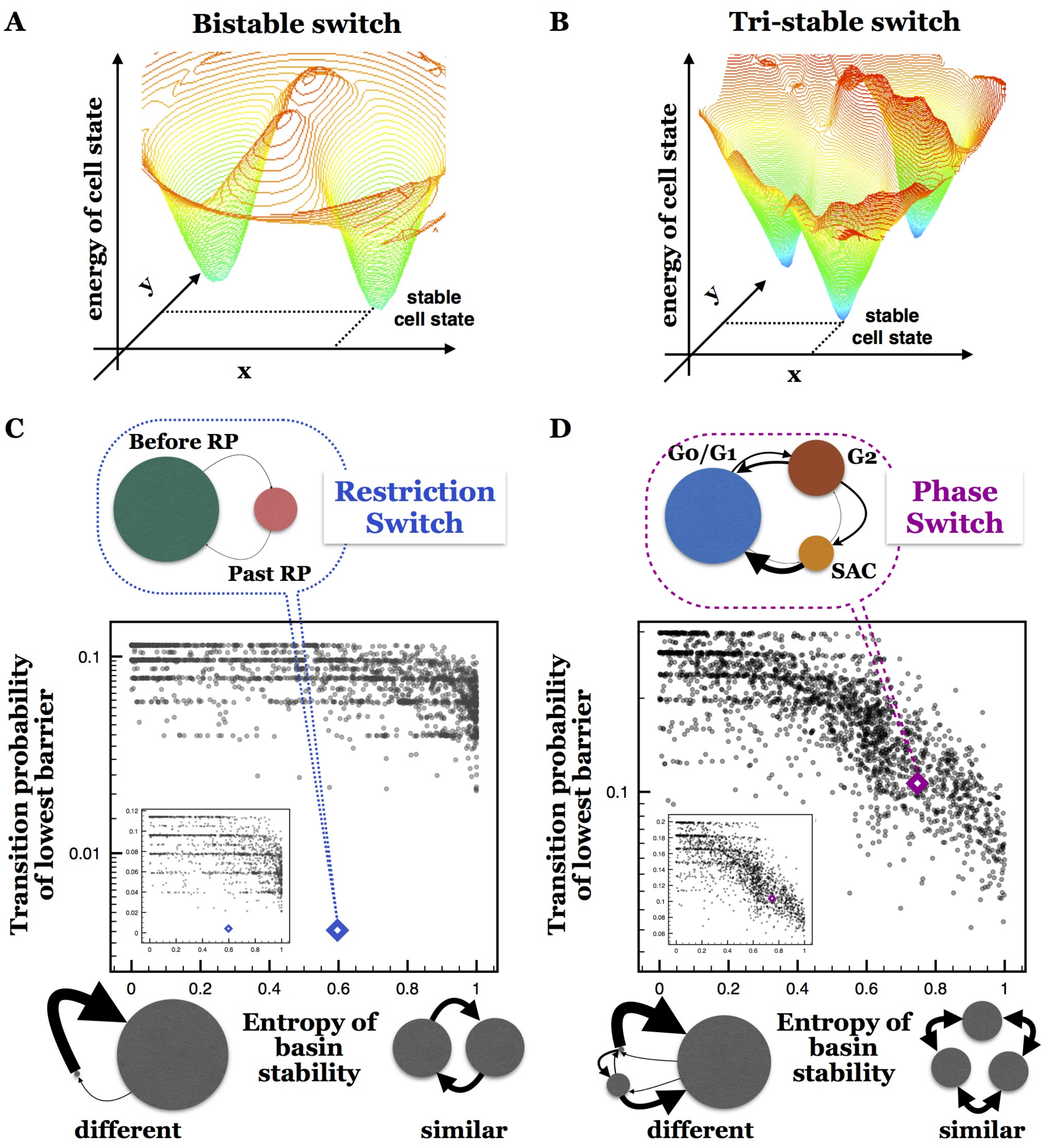

Figure S1: **Transition probability across the lowest barrier in *Cell Cycle* switches vs. matched random networks. (A-B)** Quasi-energy landscape of a bistable (A) and tri-stable (B) switch, visualizing the attractor basins leading to stable cell states (phenotypes), and the regulatory barriers cells have to overcome to transition between them. **(C)** *Top:* phenotype-level dynamics of the *Restriction Switch* (node size: phenotype stability; arrow size: probability of noise-drive phenotype-transitions). *Bottom:* maximum transition probability vs. basin stability entropy, defined as $H_B = -\sum_i^{N_a} P(i) \cdot \log[P(i)]$, where $P(i)$ is the overall probability of observing the system in the basin of attractor i (blue diamond: *Restriction Switch*; grey dots: random networks with $N = 6$ nodes, $K = 16$ links, $p_0 = 0.63$ and $N_a = 2$ attractors; 2000 samples; gate error probability $p_E = 0.02$). **(B)** *Top:* phenotype-level dynamics of the *Phase Switch*. *Bottom:* maximum transition probability vs. basin stability entropy (purple diamond: *Phase Switch*; grey dots: random networks with $N = 11$ nodes, $K = 40$ links, $p_0 = 0.599$ and $N_a = 3$ attractors; 2000 samples; gate error probability $p_E = 0.02$). Insets show the same data with linear y-axis.

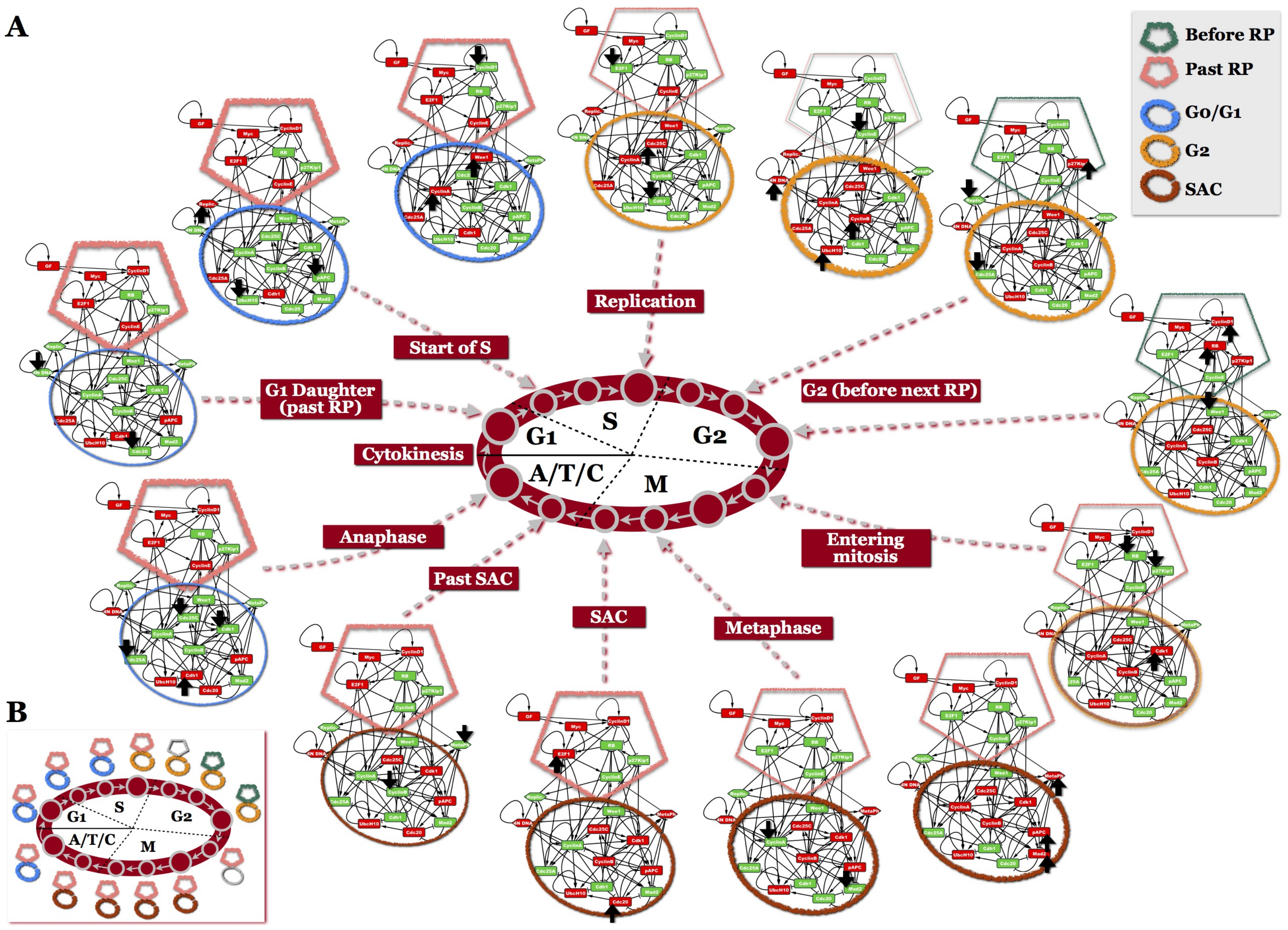

Figure S2: **The Cell Cycle phenotype.** *Black up/down arrows:* nodes that turn ON/OFF between time-steps; *outlines around each switch:* overlap between its node-states and the most similar switch-phenotype, as indicated by Figs. 2D-E (*color:* switch-phenotype; *thickness:* overlap).

4. Molecular mechanisms of early Restriction Point passage

As **Figure S3A** indicates, our cell cycle model enters the cell cycle from *G*0 after a *G*1 phase that is significantly longer than in continuously cycling cells (7 time-steps between the start of mitogen stimulation and the first *R*eplication step, vs. 1 *G*1 time-step in continuously cycling cells). Mitogen independence, however, is reached after the first 3 time-steps (**Fig. S3B**).

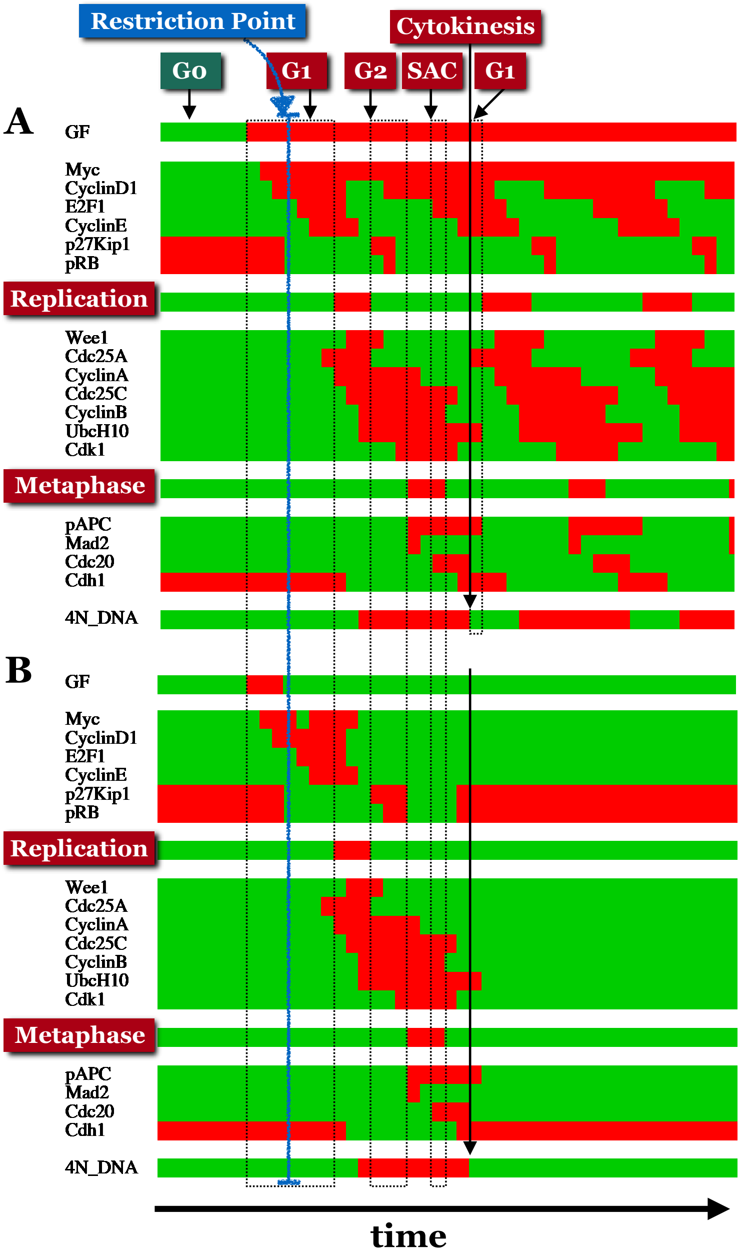

Figure S3: **Simulation of mitogen-exposure and mitogen-pulse, classically used to pinpoint the location of the Restriction Point. (A)** Cell cycle entry from prolonged *G*0. **(B)** Smallest mitogen-pulse that leads to completion of a cell cycle. *Red/green squares*: ON/OFF node states; *blue line*: time-point at which the *Restriction Switch* flips.

Our model differs from previous cell cycle models in a few important molecular details. First, active *E2F* OR *Myc* can induce *E2F* transcription in the absence of active *Cyclin A* AND *RB* [30, 31, 32], and conversely, active *E2F* alone can induce *Myc* [33, 34]. In addition to *Myc*, active *E2F* also impacts *Cyclin D* activation [35]. As the precise regulatory logic of *Cyclin D* as a function of growth factors, *Myc* and *E2F* is not known, we attempted to take into account both production and stability of *Cyclin D*. To this end, we assumed that if *GF* OR *CyclinD* are present, either *Myc* OR *E2F* can maintain above-threshold

CyclinD activity. In the absence of *GF* or active *CyclinD*, both Myc AND *E2F* are required to turn it back ON.

In late G1, neither the *E2F* ⇄ *Myc* or *E2F* ⇄ *Cyclin D* feedback are necessary for restriction point passage, as *Cyclin A* does not block *E2F* and *Cyclin E* maintains *RB* repression. At the G2 → M transition, however, these feedback mechanisms are critical for early commitment to the next cycle. In G2, the *Restriction Switch* is poised for commitment: growth factors keep *Myc* ON, driving re-expression of *Cyclin D* (lost during replication) [36], and leading to *RB* / *p27Kip1* suppression (**Fig. 4A**). *Cyclin A/Cdk2* activity, however, keeps *E2F*-mediated transcription at bay [37]. The moment *Cyclin A* is deactivated at the G2 → M boundary by *Cyclin B* competition for *Cdk1* and degraded by *APC*, the circuit can achieve growth factor-independence for another cycle. This requires *E2F* → *Cyclin D* feedback (**Fig. S4**); in its absence, loss of *Cyclin A* leads to reactivation of *RB* and *p27Kip1*. Thus, lack of *E2F* → *Cyclin D* induction delays mitogen independence until *E2F* can prevent loss of *Cyclin D* indirectly, by keeping *Myc* active (in our model, this occurs in early Anaphase). As **Fig. S4A** indicates, the absence of *E2F1* ⟶ *CyclinD* feedback regulation is critical for Restriction Point passage immediately after M-phase entry. Its absence delays commitment until the Metaphase-Anaphase transition, where *E2F* is already ON and the *E2F* ⟶ *Myc* feedback can keep *Myc* active. Lack of the *E2F* ⟶ *Myc* feedback alone, on the other hand, is insufficient for delaying commitment in our model

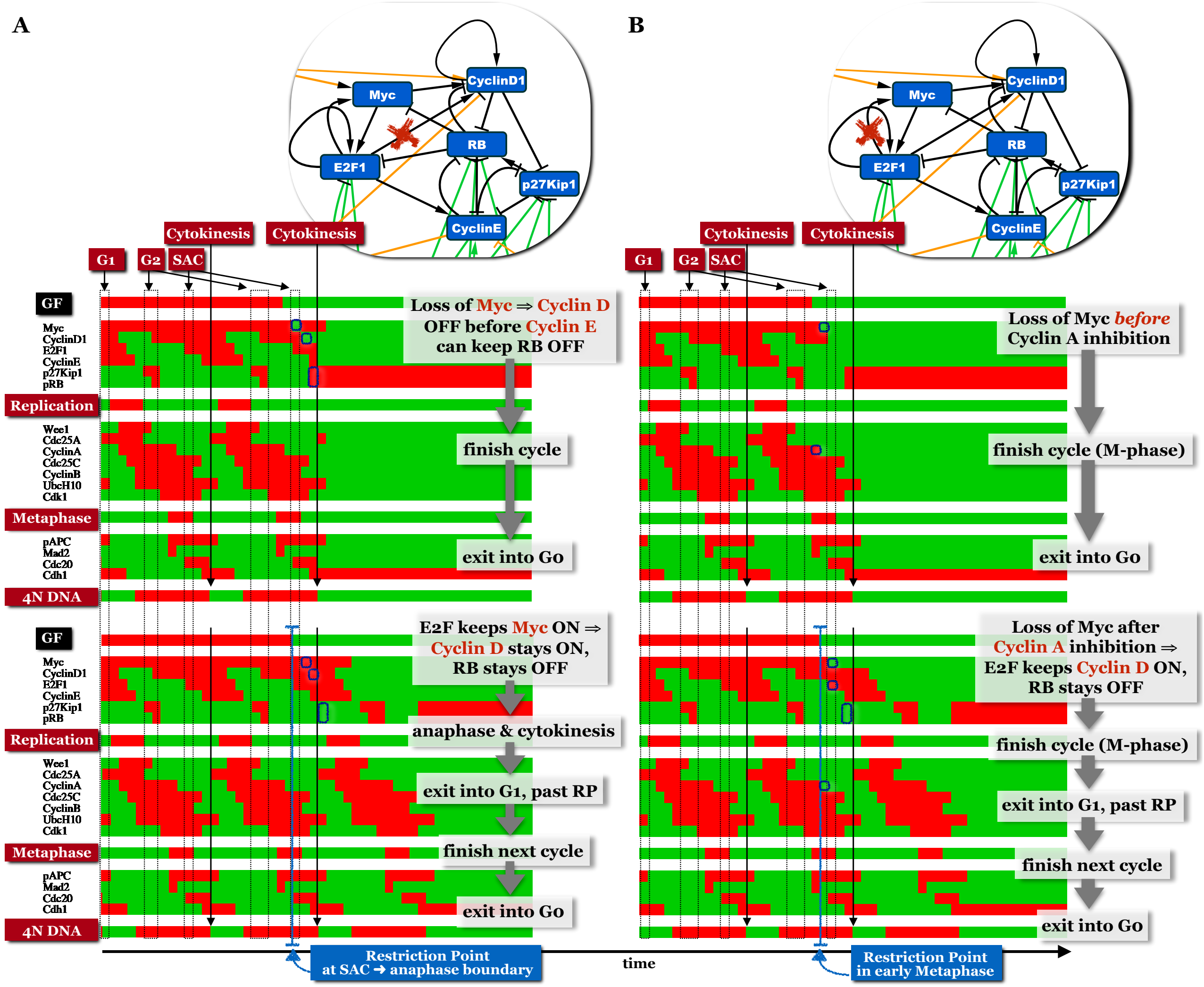

Figure S4: **In silico mitogen withdrawal in modified Cell Cycle models without** *E2F1* → *CyclinD* **or** *E2F1* → *Myc* **feedback. (A)** *Top*, Lack of *E2F1* ⟶ *CyclinD* activation in the *Restriction Switch* (red X). *Middle*, Mitogen withdrawal in Metaphase results in loss of *CyclinD* followed by reactivation of *RB* and *p27Kip1*, blocking commitment to the next cycle. *Bottom*, Mitogen withdrawal at the Metaphase → Anaphase transition after *E2F1* is already active, allowing it to sustain *Myc* and *CyclinD*. *Dark blue boxes*: states of *Myc*, *CyclinD*, *RB*, *p27Kip1* and *CyclinA* in two divergent mitogen withdrawal experiments: *top*, the latest moment of withdrawal that does not lead to RP passage, and *bottom*, the earliest moment of withdrawal that results in RP passage.

(**Fig. S4B**). In this case, *E2F1* is sufficient for keeping *Cyclin D* activity up during Metaphase, and *RB* remains OFF. It is worth noting, however, that this behavior depends on the exact choice of a $CyclinD = f(Myc, E2F1, GF, RB, CyclinD)$ Boolean gate (Supplementary Table S3).

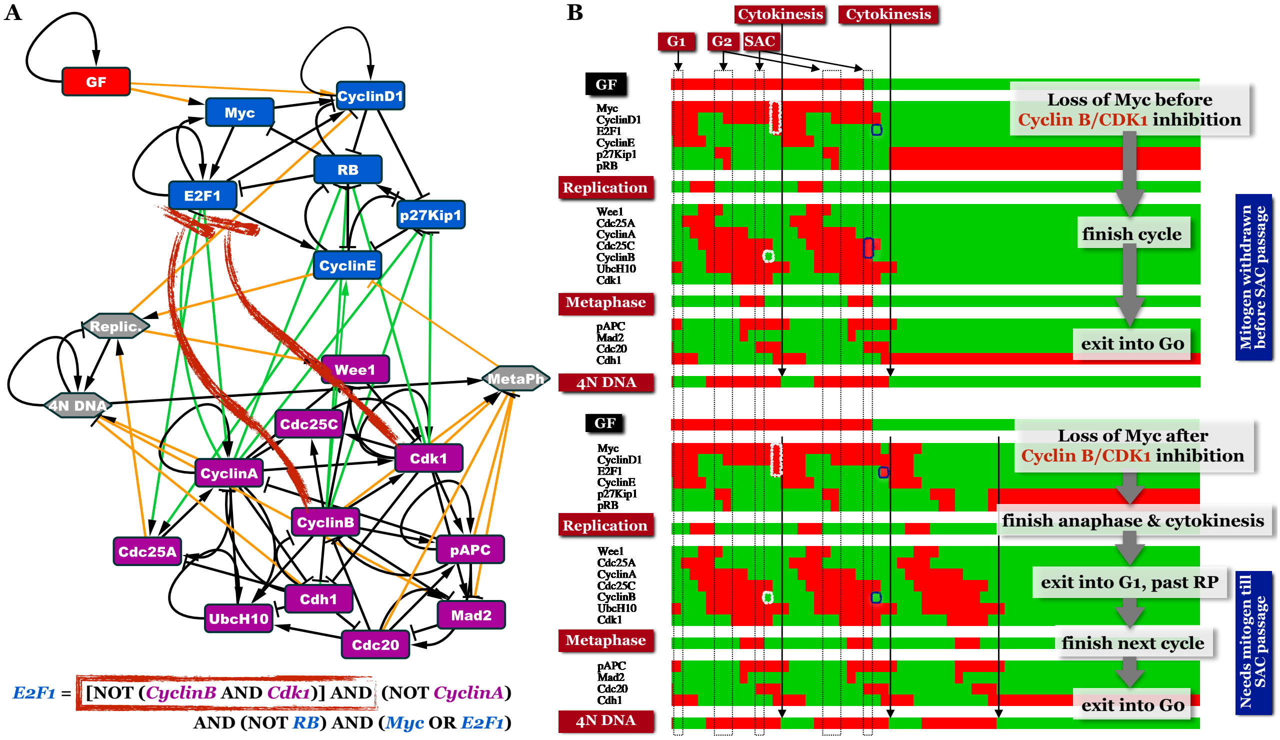

Figure S5: **In silico mitogen withdrawal in a modified Cell Cycle model with** *CycB/Cdk1* ⊣ *E2F1*. **(A)** *Top*, This model differs from the one on **Fig. 2F** in that the presence of *E2F1* is inhibited by active CycB/Cdk1 complexes (red arrows). *Bottom*, Boolean gate governing *E2F1* update (*red box*: modification from model in **Fig. 2F**; note that in both models the *Cyclin A* node denotes active, *Cdk2* or *Cdk1*-bound *Cyclin A* complexes, while the *Cyclin B* node represents *Cyclin B* expression and thus requires active *Cdk1* for its activity). **(B)** *Top*, Mitogen withdrawal at the last time step before the Metaphase → Anaphase transition does not result in *E2F1* reactivation, as *CyclinB/Cdk1* is still active at this point. *Bottom*, Mitogen withdrawal immediately after the Metaphase → Anaphase transition allows *E2F1* reactivation, as *CyclinB/Cdk1* is no longer active. (*White boxes*: moment of *Cyclin B* degradation, followed by *Myc, CyclinD1* and *E2F1* accumulation in continuously cycling cells; *dark blue boxes*: states of *Cyclin B* and *E2F1* in two divergent mitogen withdrawal experiments: *top*, the latest moment of withdrawal that does not lead to RP passage, and *bottom*, the earliest moment of withdrawal that results in RP passage.)

Most published Cell Cycle models include *E2F1* inhibition by *CyclinB/Cdk1*, in addition to *CyclinA* ⊣ *E2F1* (**Fig. S5**, red arrows). This inhibition delays commitment to an additional round of division until after *Cyclin B* is degraded, which takes place at SAC passage.

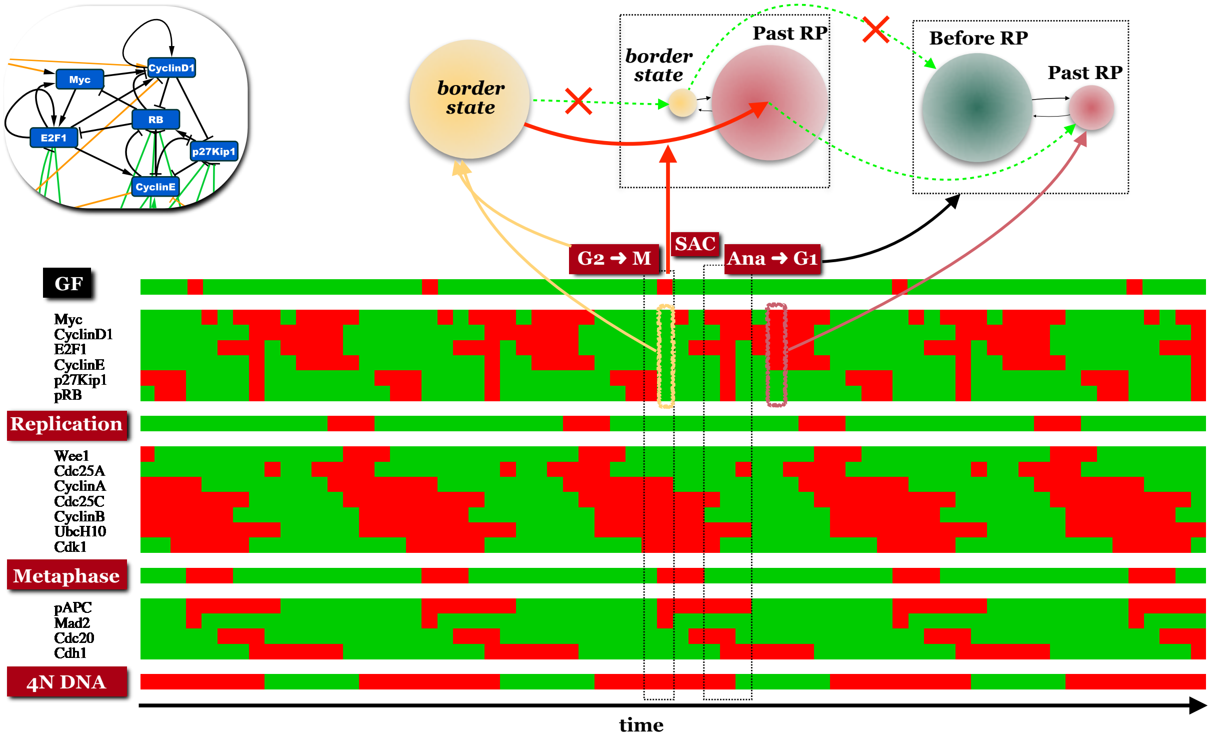

Figure S6: **Minimal cyclic growth factor pulse for continuos cycling.** *Left*, *Restriction Switch* with no *p21*. *Top right*, Attractor basins of the *Restriction Switch* in the changing environment provided by the *Phase Switch* in the absence of growth factors. *Green dashed arrows*: trajectory of the *Restriction Switch* in a growth-factor free environment; *red X and arrows*: effect of the 1-step *GF* pulse on the *Restriction Switch* trajectory. *Bottom right*, time-course of the cell cycle model driven by minimal *GF* cyclic growth factor stimulation.

5. Modeling the p21-positive Restriction Switch

Spencer et al. have shown that a cell's decision to exit into G0, as opposed to immediately entering the next cycle, is in fact controlled by nuclear *p21* [38]. Moreover, high levels of *p21* in quiescent cells were shown to render their cell cycle entry stochastic [39]. This *p21*-induced cell bistabilty is caused by positive (double-negative) feedback between *p21* and active *Cyclin E/Cdk2*. On one hand, *Cyclin E/Cdk2* activates the *SCF/Skp2 complex*, which then degrades *Cyclin E/Cdk2*-bound and phosphorylated *p21* [40]. On the other hand, *p21* not only blocks *Cyclin E/Cdk2* activity, but it inhibits *Cyclin D* and thus interferes with the mitogen signal that turns on *Cyclin E* in the first place.

To test the effect of *p21* on bistability of the *Restriction Switch*, we created a ***p21*-high** version of our cell cycle model (**Fig. S7A**). Here, the *Restriction Switch* includes a $p21_B$ node, corresponding to active, nuclear *p21* in a cell line with high basal *p21* expression (though not as high as *p53*-driven *p21*) (red border node in **Fig. S7A**). To account for the *Cyclin E/Cdk2* ⇄ *p21* feedback loop, we assume that $p21_B$ activity (and/or localization) is inhibited by *CyclinE* OR [*CyclinA* AND *Cdc25A*] (corresponding in out model to *Cyclin A/Cdk2*), but it is otherwise active. $p21_B$, in turn, inhibits *CyclinD* unless both *Myc* AND *E2F* contribute to *CyclinD* transcription, AND [*CyclinD* OR *GF*] are (already) present. In addition, *RB* cannot be ON to weaken *E2F*, unless both *CyclinD* AND *GF* are active. We did not include direct inhibition of *Cyclin E* by $p21_B$, as it would irrevocably lock the entire cell cycle model into a growth arrested G0 state. That said, this interaction is likely to be relevant for modeling G1 arrest by *p53*-driven *p21*, and/or mitotic catastrophe-driven endoreduplication [41].

In contrast to the *p21*-null *Restriction Switch*, the *p21*-high switch is bistable in both the absence and presence of growth factors (**Fig. S7B**). In agreement with experiments, mitogens alter the stability of *Before RP* versus *After RP*, but do not deterministically commit a G0 cell to proliferation. In the absence of randomness, the *p21*-high model can actually maintain quiescence (G0) following *GF* exposure (**Fig. S7D**). A cell cycle attractor also exists, indicating that once the *Restriction Switch* makes a noise-driven transition past its barrier to *After RP*, it maintains its commitment. In fact, the *p21*-high *Restriction Switch*is only deterministically committed to *After RP* during Metaphase (**Fig. S7B, S7D**). Thus, a well-timed growth factor pulse at the start of mitosis can keep the *p21*-high network cycling (**Fig. S7E**), even though a similar pulse does not trigger G0 exit.

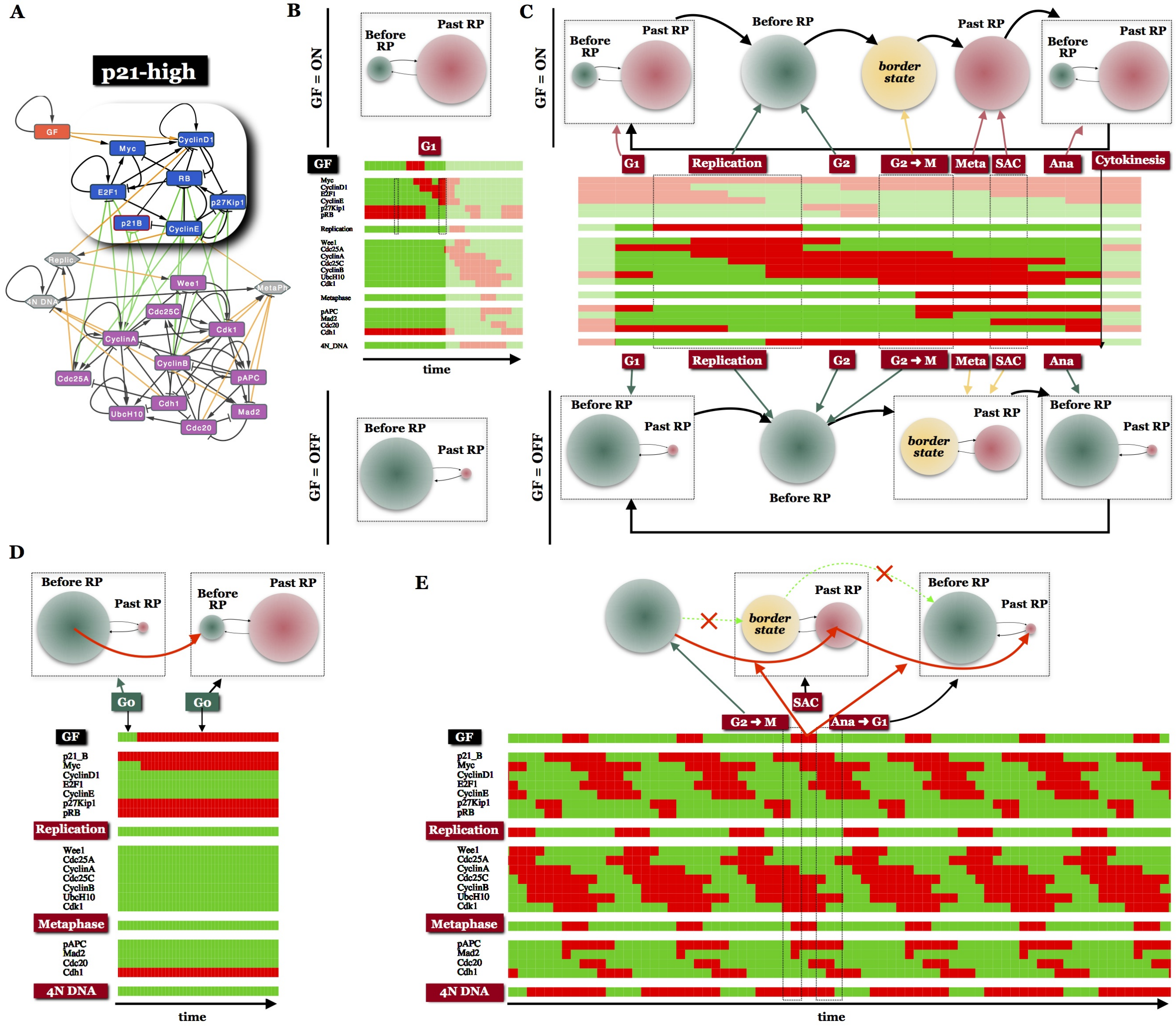

Figure S7: **Cell cycle model with a** ***p21*****-high** ***Restriction Switch*****.** **(A)** A *p21*-high *Restriction Switch* embedded in the Cell Cycle network ($p21B$ marked by red border). **(B)** Attractor basins of the *p21*-high *Restriction Switch* when its internal dynamics are constrained by the *Phase Switch* in G0 (*middle*), and *GF* locked ON (*top*) or OFF (*bottom*). **(C)** Cell cycle phase-dependent attractor landscape of the *p21*-high *Restriction Switch* constrained by a changing *Phase Switch* (*middle*) and *GF* locked ON (*top*) or OFF (*bottom*). Arrows map phases of the cell cycle to attractor basins of the *p21*-high *Restriction Switch*. **(D)** Molecule-level time-course of the *p21*-high cell cycle model upon *GF* exposure (*bottom*). *Top*, attractor basins of the *Restriction Switch* in the absence (*left*) and presence (*right*) of growth factors; *red arrow*: effect of turning ON the *GF* node when this model is in *G0*. **(E)** Molecule-level time-course of the *p21*-high cell cycle model driven by minimal cyclic *GF* stimulation (*bottom*). *Top:* attractor basins of the *Restriction Switch* in the changing environment provided by the *Phase Switch* in the absence of growth factors. *Green dashed arrows*: trajectory of the *Restriction Switch* in a growth-factor free environment; *red X and arrows*: effect of the 3-step *GF* pulse on the *Restriction Switch* trajectory.

6. Modeling the effect of cell-type specific baseline *p21* expression

In order to model cell-type specific differences in restriction point passage, we united the two models by adding a new 1-node switch representing p21 transcription (*p21TR*), required but not sufficient for nuclear p21 protein activity (p21-TR model, **Fig. S8A**). By stochastically tuning the probability of both *GF* = ON and *p21TR* = ON, we again observe stochastic cell fate decisions in the entire $p_{p21} \in (0, 1)$ range, in the form of extremely short cytokinesis-to-S intervals interspersed with long exits into a

G0-like state (**Fig. S8B**). These G0 pauses have exponential time-window distributions (**Fig. S8B**) and escape rates determined by the combinatorial effect of mitogen dose and p21 transcription ($\kappa(p_{GF}, p_{p21})$ on **Fig. S8C**). As expected, increased p21 transcription introduces a p_{GF} threshold below which G0 escape does not occur. More intriguing is the sharpening effect of p21 on the growth factor dependency of G0 escape (**Fig. S8C**), rendering it ultra-sensitive to mitogens. Thus, cells with high basal p21 expression are expected to show high levels of heterogeneity in cell cycle entry when exposed to non-saturating levels of mitogen, in agreement with experimental observations [39].

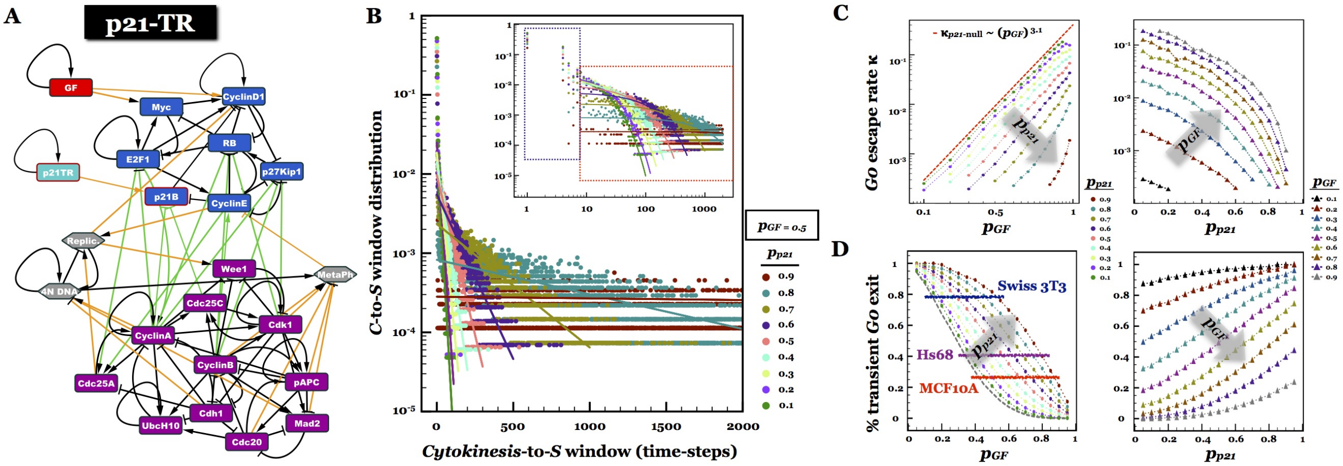

Figure S8: **Cell cycle model with an external $p21_{TR}$ toggle switch controlling the presence of $p21_B$ protein. (A)** Network in which the *p21*-high *Restriction Switch* is sensitive to the external $p21_{TR}$ node driving *p21* transcription. **(B)** Distribution of the C-to-S window T as a function of p_{p21} ($p_{GF} = 0.5$). Straight lines are exponential fits of the $p(T)$ distribution; $p(T) \sim \exp[-\kappa(p_{GF}, p_{p21}) \cdot T]$ for $T > 7$. *Inset:* distributions on log-log scale; *blue box:* cells that pass the restriction point in early Metaphase; *red box:* cells that transiently exit into G0. $N_{\text{div}} = 10.000$ divisions / condition, provided they occurred within $t_{\max} = 5 \cdot 10^7$ time-steps (fewer otherwise). **(C-D)** Escape rate κ from the transient G0 state (C) and percentage of cells that transiently exit the cell cycle (D), as a function of growth stimulation (p_{GF}, *left*) and *p21* transcription (p_{p21}, *right*). *Horizontal lines:* experimentally reported percentages in three mammalian cell lines [38], indicating that small intrinsic differences in $p21$ transcription can account for large differences in cell cycle exit at similar levels of growth stimulation.

7. Randomized networks with high Attractor Modularity Measure

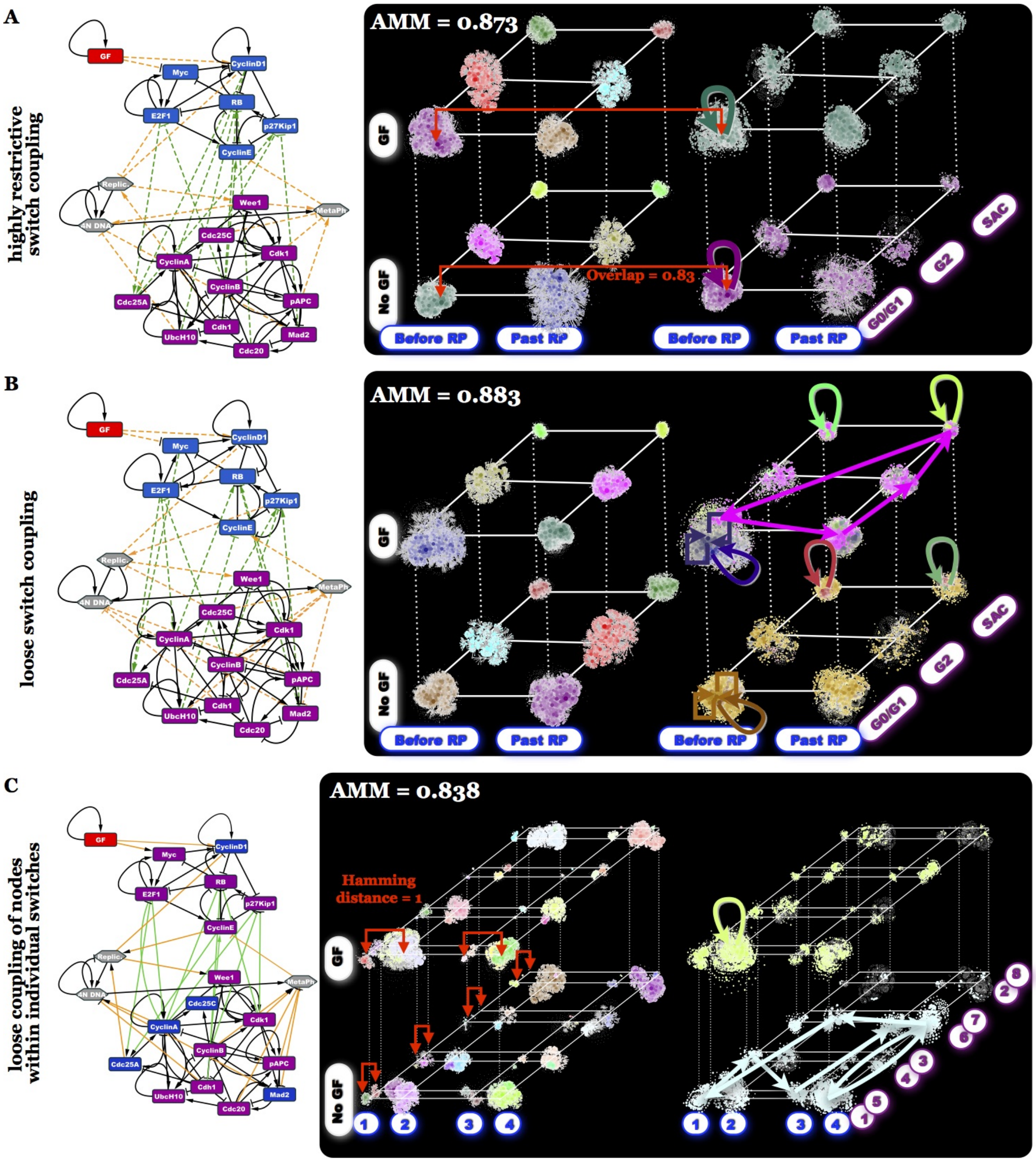

Figure S9: **Randomized networks with high** AMM**.** **(A)** Network in which the two cell cycle switches have been randomly coupled (*left*) in such a way that the system expresses the same *Restriction Switch - Phase Switch* phenotype combination ($\{$*Before RP, Go/G1*$\}$), with or without *GF* (*right*, self-links). *Red arrows:* matched pure (*middle*) and coupled (*right*) switch-phenotype combinations with overlap of ~ 0.83. **(B)** Network with randomly coupled cell cycle switches (*left*) where the switches do not severely restrict each other's phenotype, and thus the system expresses several similar switch-phenotype combination (*right*, self-links). **(C)** Cell cycle network with randomized switch assignment (*left*). Weak connections inside these switches lead to multiple similar phenotypes in both the 6-node Switch 1 (*right*, x-axis/blue labels) and 11-node Switch 2 (y-axis/purple labels). *Red arrows*: switch-phenotypes that differ by the ON/OFF state of a single molecule. The global fixed-point is very similar to pure $\{$2,1,GF$\}$, while the limit cycle toggles close to several switch-phenotype combinations.

SUPPLEMENTARY METHODS

1 Mapping the attractor landscape of large Boolean networks

In order to approximate $\Pi(s)$ for large networks (see main *Methods 3*), we adapted the sampling algorithm proposed by Mann & Klemm to synchronous noisy Boolean dynamics [42]. Our implementation tracks the attractor membership and number of visits of states sampled by noisy dynamic runs. Briefly:

For $r = 1$ to $N_{\text{init}} = 50$

$\rightarrow$ start from a randomly chosen initial state in phase space, s_{init}

$\rightarrow$ for $t = 1$ to $N_{\text{series}} = 50000$

- if s is NOT a known member of an attractor basin, we temporarily interrupt the noisy time series to find its basin membership. This is accomplished using synchronous update, and thus any new state uncovered during this detour is not marked as "visited":
  - mark the system's present state as s_{now}
  - generate a row of states, starting with s_{now} and updating the system's state using noise-free Boolean rules until:
    - $*$ a state with known attractor basin membership b is reached
      - · add entire row of states to basin b, with 0 visits each
    - $*$ OR - the row enters a loop
      - · create a new attractor basin b
      - · add entire row of states to basin b, with 0 visits each
    - $*$ reset the system's present state to $s = s_{\text{now}}$ (resuming noisy dynamics)
- end if
- increase the visit counter of state s_{now}, as well as its basin b
- enumerate every possible state the system could reach from s_{now} with a *single* node-state flip (i.e., *all neighbors* at Hamming distance 1), identify their attractor membership (without marking them as visited), calculate the probability of this accidental transition, and record it in a growing sum of attractor-basin transitions that, though not *observed* on our actual time-trace, could in theory contribute to noise-driven transitions between basins (detailed in [42])
- update the system's state according to Boolean rules in the presence of noise (each gate returns the wrong output with probability p_E)

Following the logic outlined by Mann & Klemm [42], our algorithm gives good transition probability estimates across attractor barriers, even for transitions that are extremely rare. More importantly, we have found that checking the attractor membership of every possible neighboring state along each noisy dynamic path allows the algorithm to "peek" into all corners of the state space (due to the small-world nature of high-dimensional hypercubes [43]), and find even the smallest basins. In fact, the algorithm is extremely slow and not so useful on Boolean networks with a very large number of small basins, but it is very well suited for our needs.

Our implementation is slightly more complicated than the above procedure, because it limits the number of states tracked within each basin b to a maximum ($N_{\text{max}} = 5000$). Consequently, every time the quota of a basin is filled, states with visitation 0 (encountered on noise-free steepest descents from s_{now} states of unknown basin membership) are only added to basin b if there are still other 0-visitation states contributing to the basin's quota. In this case, a random one of these is replaced by the newly encountered state. Once every member of the basin has at least 1 visit, these intermediates are ignored. Moreover, every time a new s_{now} state is added to the basin (visitation $v = 1$), a random member state from the lowest visitation bracket v_{min} is replaced by s_{now} with probability $1/(2^{v_{\text{min}}})$.

2 Visualizing the attractor landscape of multi-switch networks

Visualizing *changes to the attractor landscape* of a multi-switch network when its switches are coupled to each other requires a novel computational tool. To this end, we start with a composite system of switches in the *absence* of links between them. These composites are straightforward to describe, as any combination of individual switch-fixed points is a fixed point of the composite. In case of the disconnected switches of the Cell Cycle model, the *Restriction Switch* has 2 and the *Phase Switch* has 3 fixed points. In addition, the growth factor (*GF*) node itself is bistable and thus acts as a stand-alone switch with 2 states. First, we sampled the attractor landscape of the *decoupled* composite system with $N_U = 2 \cdot 3 \cdot 2 = 12$ attractors (**Fig. 3A**). Second, we added back the links running between switches, and sampled the attractor landscape of the coupled system ($N_C = 2$ attractors, **Fig. 3B**). In order to visualize the resulting *change* in the attractors and their basins, we generated a *single* layout for *all* sampled states (regardless of which of the two, decoupled or coupled sampling processes visited them). To this end, we collected all sampled states into groups corresponding to their attractor basin membership in the *decoupled* composite system. We then visualized each *switch-phenotype combination state-group* using a 2D implementation of the Fruchterman-Reingold network algorithm [44], adapted to weighted links, defined as follows:

- Let M^U_{ij} be the transition probability with which the decoupled system goes from state s_i to s_j, in one type step, in the presence of noise: $p_E^h(1-p_E)^{N-h}$, where h is the Hamming distance between s_j, and the state s_k that follows s_i in one time-step in the *absence* of noise. M^C_{ij} denotes the same probability for the coupled system.
- For the purposes of network layout, we add a directed link of weight $M^U_{ij} + M^C_{ij}$ from s_i to s_j, provided $M^U_{ij} + M^C_{ij}$ is above a small threshold ($w_{\text{min}} = 10^{-9}$). For states that are isolated from the rest of the basin by this thresholding, we keep their strongest connection even though it is below-threshold.
- The Fruchterman-Reingold algorithm displays networks by finding a balance between repulsive forces acting between all node pairs (thus spreading the nodes), and attraction forces between directly linked node pairs. In our implementation, the force of attraction depends on link weight: the force between a node pair $s_i - s_j$ is weighted by the sum of weights along $s_i \to s_j$ and $s_i \leftarrow s_j$.
- Using the Fruchterman-Reingold algorithm we generate a *single* layout for all sampled states, regardless of which dynamics visited them.

The visualization *compares* the attractor membership and visitation probability of these states between the decoupled and coupled dynamical systems. In order to convey the visitation probability of individual states, we mapped the visitation probability of each state onto node size and color saturation (**Fig. 3**; large nodes with saturated color denote high visitation probability, small nearly white nodes have low visitation probability; nodes only sampled by the decoupled or coupled system's dynamics appear as white points). We then arranged the individual attractors of the *decoupled* system along a 3D grid, where the phenotypes of the 3 switches are arranged along 3 axes, and every position in this grid represents a unique switch-phenotype combination (**Fig. 3A**). Next, we visualized the state transition graph and attractor landscape of the coupled system, while *preserving the layout of states* used for the decoupled system, and only changing the color and size of each state-node to show its attractor membership and visitation probability in the coupled system's dynamics (**Fig. 3B**). The self-arrow overlaid on **Fig. 3B** marks the *G0* fixed point of the coupled dynamics. Thanks to the visualization, the switch-phenotype combinations the *coupled* system uses to create its global phenotypes can be read off as coordinates in 3D. Moreover, the arrows tracing the cell cycle give a fair indication of how each individual switch toggles its phenotype as the cell cycles through division.

In order to keep **Figure 3** visually clean, we chose to only show error-free (deterministic synchronous) transitions of the decoupled/coupled dynamics that start and end in the same switch-phenotype combinations. Consequently, nearly all states within individual attractor basins on **Fig. 3A** (decoupled system) are linked into a single network-cluster. This is because synchronous transitions from nearly every state lead to states within the same basin (the few exceptions are states that were only sampled by the coupled dynamics, and the state they transition to was similarly not visited by the decoupled network). In contrast, isolated high-visitation probability states are common on **Fig. 3B** (coupled dynamics). Deterministic transitions from these states lead to states located in *another* basin, representing a different switch-phenotype combinations, as shown on **Fig. S10**. The dense network of transitions running between basins highlight robust, predictable transitions at the switch-phenotype level, as the system converges on an attractor. Notably, in the absence of growth factors, all switch-state combinations lead to *G0* via the appropriate phenotype-sequences observed in cells (e.g. the *Past RP G1* state goes through *G2* and the *SAC* mitotic checkpoint before setting in *G0*.

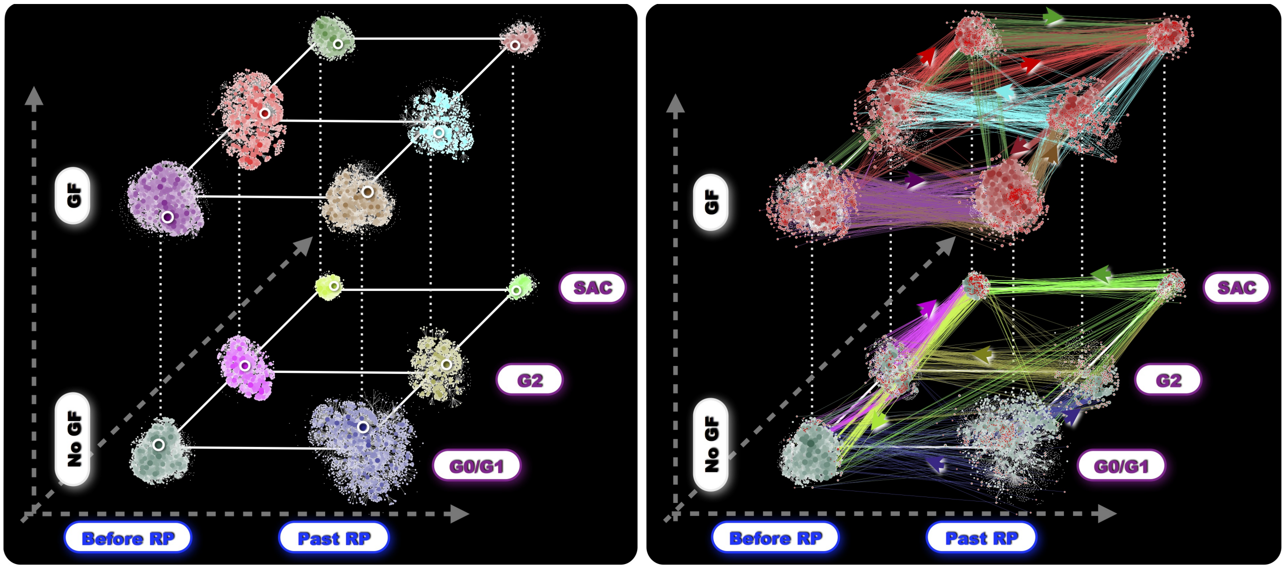

Figure S10: **Full state transition graph of the coupled *Cell Cycle Model*.** *Left:* switch-phenotype combinations if decoupled switches; *right:* global attractors of the coupled system. In addition to the deterministic state transitions among states that fall within a single switch-phenotype combination (also shown in Fig. 3B), colored lines on the right panel indicate deterministic transitions among states in *different* switch-phenotype combinations (*link color:* switch-phenotype combination of origin; *link width:* $\log[\Pi(s)]$ of node of origin; *colored arrows with white shadow:* direction of prominent link-bundles; *red dot:* node of origin).

3 Mapping a sequence of global network states onto a sequence of switch-phenotype combinations

In order to define a meaningful mapping algorithm between an arbitrary dynamic trajectory of the multi-switch network (a sequence of global network states), we first introduce a few notations:

- Let $\mathbf{G}_m, m = 1, \ldots, M$ denote the M isolated switches of the regulatory network, and $\mathbf{G}_c$ the coupled system itself. $\mathbf{G}_m$ and $\mathbf{G}_c$ are deterministic dynamical systems that map a state of the system $s(t)$ to the state in the next time step: $s(t+1) = \mathbf{G}(s(t))$, where $s(t)$ is a Boolean vector with N elements. For the coupled system, the vector has N_c elements (the total number of regulatory nodes), while vectors of switch m have N_m elements.

- Let $D^c = \{s_k, k = 1, \ldots, n \mid s_{k+1} = \mathbf{G}_c(s_k)\}$ denote an arbitrary dynamic trajectory of the multi-switch network with the initial condition s_1 and n time-steps.

- Let $Q_i^c = \{s_k^{c\to i}, k = 1, \ldots, L_i^c \mid s_{k+1}^{c\to i} = \mathbf{G}_c(s_k^{c\to i}); s_1^{c\to i} = \mathbf{G}_c(s_{L_i^c}^{c\to i})\}$ denote the attractor i of the coupled system $\mathbf{G}_c$ (N_c nodes). Q_i^c stands for a dynamic trajectory of $n = L_i^c$ states with cyclic boundary conditions. Fixed-point attractors (steady states) have length $L_i^c = 1$, while limit cycles are characterized by $L_i^c > 1$. Let q_c be the number of attractors of the $\mathbf{G}_c$ system.

- Similarly, let $Q_j^m = \{z_l^{m\to j}, l = 1, \ldots, L_j^m \mid z_{l+1}^{m\to j} = \mathbf{G}_m(z_l^{m\to j}); z_1^{m\to j} = \mathbf{G}_m(z_{L_j^m}^{m\to j})\}$ denote attractor j of switch $\mathbf{G}_m$ and q_m denote the number of attractors in of the $\mathbf{G}_m$ switch network.

- Let $H(s_a, s_b)$ or $H(z_a, z_b)$ denote the Hamming distance between a pair of states s_a, s_b or z_a, z_b in $\mathbf{G}_c$ or $\mathbf{G}_m$, respectively. The normalized Hamming distance $h(s_a, s_b) = H(s_a, s_b)/N_c$.

- Let $O(s, z^m)$ denote the *normalized overlap* between an arbitrary state s of the coupled system $\mathbf{G}_c$ and an arbitrary state z^m of switch $\mathbf{G}_m$:

$$O(s, z^m) = 1 - H(s^{\in m}, z^m)/N_m,$$

  where $H(s^{\in m}, z^m)$ is the Hamming distance between z^m, and the N_m-length subset of node states within the coupled state s which correspond to switch m, denoted $s^{\in m}$ (**Fig. S11**).

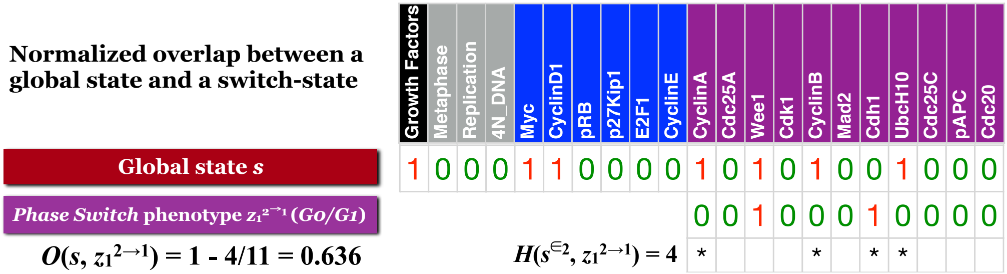

Figure S11: **Defining the normalized overlap.** The normalized overlap of a switch-state z_m (e.g., *Phase Switch* state *G0/G1*) with a global network state s (e.g., the *G2 Arrest* attractor) is computed from the Hamming distance between the switch-state (bottom row of 0,1 values), and the state of switch nodes in the global state, $s^{\in m}$ (top row of 0,1 values). The normalized overlap is computed as $O(s, z^m) = 1 - H(s^{\in m}, z^m)/N_m$.

Our goal is to quantify the degree to which a dynamical trajectory of the global (coupled) system's dynamics, D^c ($k = \{1, \ldots, n\}$), replicates different phenotypes (attractors) of the m switch, and use this overlap measure to map the global sequence D^c onto the most appropriate sequence of switch-phenotype combinations. As long as every phenotype Q_j^m of every switch is a stable state (fixed-point), this requires identifying the most similar switch-m attractor for each state s_k along the trajectory D^c. Developing a general measure applicable to networks with arbitrary switch-attractors required a meaningful definition of overlap between an arbitrary global sequence and a cyclic switch-phenotype. Before we detail this measure, however, we will deal with the fact that in general, a global trajectory D^c may sequentially pass through the attractor basin of *multiple* switch-phenotypes. As the cell cycle in **Fig. 2G** illustrates, it is possible for the global dynamics of a multi-switch system to have a limit cycle, even if all switch-attractors are fixed-points. In this example, the global cell cycle attractor does not simply map onto individual switch-phenotypes. Rather, it is an emergent rhythm driven by influences between switches: the ***Restriction Switch*** in its *past-RP* state toggles the ***Phase Switch*** from *G0/G1* into *G2* and then *M*, which, in turn, toggles the ***Restriction Switch*** back to its *before-RP* state. The presence of growth factors flips the ***Restriction Switch*** again, driving a cyclic behavior.

3.1 Flyby of the global trajectory D^c near switch-attractor Q_m^j

In order to identify the continuous segment(s) of an open or cyclic global trajectory D^c which fall into the neighborhood of the Q_m^j switch-phenotype, we first calculate the normalized overlap $O(s_k, z_l^{m\to j})$ between each state s_k along D^c, and state $z_l^{m\to j}$ of switch-m phenotype (attractor) j (**Fig. S12**). Next, we map each global state s_k onto a list of switch-attractor states $J^{D\to m}(k)$ for which the normalized overlap between s_k and all switch-m attractor states is maximal (links on **Figs. S12C-D**):

$$J^{D\to m}(k) = \{(j,l) \mid O(s_k, z_l^{m\to j}) \geq O(s_k, z_p^{m\to r}),\ \forall\, r = 1,\ldots,q_m;\ \forall\, p = 1,\ldots,L_j^m\}.$$

Note that s_k may be equidistant to several switch-attractor states, resulting on more than one $z_l^{m\to j}$ switch-attractor state associated to $J^{D\to m}(k)$ (e.g., global state 5 on **Fig. S12D**).

In order to compute $J^{D\to m}(k)$, we first create an overlap matrix with coupled states s_k as its rows, and all switch-m attractor states as its columns (**Fig. S13, left**). Next, we pick the maxima of each *row* and set all other elements to 0, obtaining the *mapping matrix* $\Im^{D\to m}$ (**S13**, right). In this matrix, only a subset of switch-m attractor columns contain non-zero elements (e.g., $j = \{3, 5, 6, 7, 8, 9\}$ on **Fig. S13**). These switch-attractors are considered to fall along the D^c trajectory. We define the *flyby* of a global sequence D^c near switch-attractor j as:

$$F_j^{D\to m} = \{s_k \mid s_k \in S(k_{\text{start}}, k_{\text{finish}})\ \&\ \exists\, l \in \overline{1, L_j^m} \mid J^{D\to m}(k) = (j,l)\ \forall\, k = \{k_{\text{start}}, \ldots, k_{\text{finish}}\}\},$$

where $S(k_{\text{start}}, k_{\text{finish}})$ denotes a continuous dynamical trajectory of $\mathbf{G}_c$:

$$S(k_{\text{start}}, k_{\text{finish}}) = \{s_k \mid s_{k+1} = \mathbf{G}_c(s_k)\ \forall\, k \in \overline{k_{\text{start}}, k_{\text{finish}} - 1}\}.$$

Flyby segments are illustrated as colored bands outside/inside the open/cyclic trajectory on **Figs. S12C-D**, and colored vertical bars in $\Im^{D\to m}$ on **Fig. S13**. Note that the first state of a closed dynamical trajectory, such as a global limit cycle, is arbitrary. A continuous segment may thus loop back from $l = L_j^m$ to $l = 1$ (e.g., teal segment on **Figs. S12D, S13**).

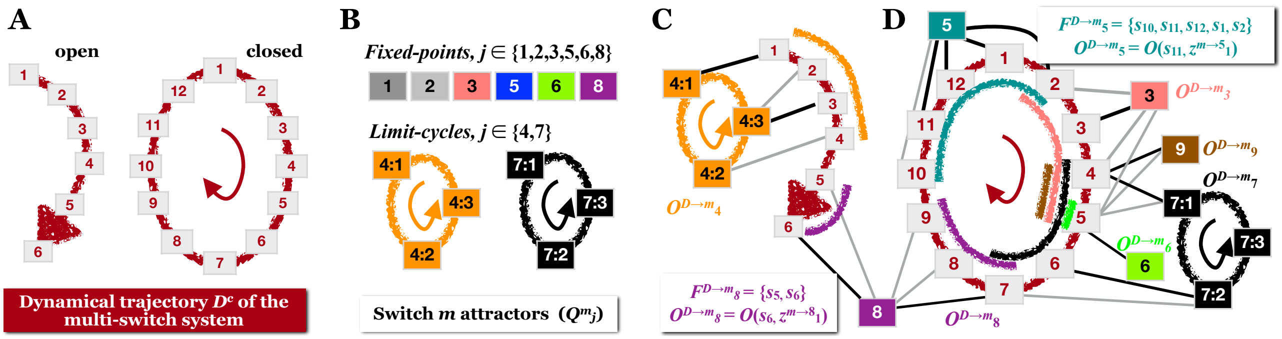

Figure S12: **Mapping global trajectories onto switch-attractors.** **(A)** Open versus closed global trajectories. **(B)** Switch-attractors Q_j^m, $j \in \{1, 7\}$ (7 fixed points, 2 limit cycles). **(C), (D)** Defining the *flyby* of open (C) and closed (D) global trajectories over the attractor basins of a switch. Each global state along D^c is connected to the switch-attractor state(s) with maximum normalized overlap (*link thickness*: normalized overlap). Flyby segments are continuous state-segments mapped into a single switch-attractor (e.g., orange global segment $\{1, 2, 3, 4\}$ on (C)). When this switch-attractor is a fixed point (e.g., $j = 8$), then the overlap of the flyby segment $O_j^{D\to m}$ equals the highest overlap along the segment (e.g., $O_8^{D\to m} = O(s_6, z_1^{m\to 8})$; see below for cyclic switch-attractors.)

Overlap matrix

Hypothetical Switch

Cyclic global state sequence

	1	2	3	4			5	6	7			8	9
				1	*2*	*3*			*1*	*2*	*3*		
1	0.45	0.45	0.73	0.82	0.73	0.82	**0.91**	0.36	0.73	0.64	0.73	0.82	0.55
2	0.36	0.36	**0.82**	0.73	0.64	0.73	**0.82**	0.27	0.64	0.55	0.64	0.73	0.64
3	0.45	0.45	**0.91**	0.64	0.55	0.64	0.73	0.36	0.55	0.45	0.55	0.64	0.73
4	0.45	0.27	**0.87**	0.45	0.18	0.27	0.36	0.55	**0.87**	0.45	0.55	0.64	**0.87**
5	0.55	0.36	**0.64**	0.36	0.27	0.18	0.27	**0.64**	**0.64**	0.55	0.45	0.55	**0.64**
6	0.55	0.36	0.64	0.18	0.45	0.18	0.27	0.36	0.45	**0.87**	0.45	0.55	0.45
7	0.27	0.27	0.55	0.45	0.36	0.45	0.55	0.36	0.55	**0.87**	0.55	**0.87**	0.36
8	0.27	0.27	0.55	0.45	0.36	0.45	0.55	0.36	0.55	0.45	0.55	**0.64**	0.36
9	0.27	0.27	0.55	0.45	0.36	0.45	0.55	0.36	0.55	0.45	0.55	**0.64**	0.36
10	0.18	0.36	0.45	0.55	0.45	0.55	**0.64**	0.27	0.45	0.36	**0.64**	0.55	0.27
11	0.27	0.45	0.55	0.82	0.73	0.82	**0.87**	0.36	**0.87**	**0.87**	0.73	0.82	0.36
12	0.27	0.45	**0.91**	0.82	0.73	0.82	**0.91**	0.36	0.73	0.64	0.73	0.82	0.36

$\Im^{D\to m}$ →

Mapping matrix $\Im^{D\to m}$ **and flyby segments** $F_j^{D\to m}$

Hypothetical Switch

	1	2	3	4			5	6	7			8	9
				1	*2*	*3*			*1*	*2*	*3*		
1	0	0	0	0	0	0	**0.91**	0	0	0	0	0	0
2	0	0	**0.82**	0	0	0	**0.82**	0	0	0	0	0	0
3	0	0	**0.91**	0	0	0	0	0	0	0	0	0	0
4	0	0	**0.87**	0	0	0	0	0	**0.87**	0	0	0	**0.87**
5	0	0	**0.64**	0	0	0	0	**0.64**	**0.64**	0	0	0	**0.64**
6	0	0	0	0	0	0	0	0	0	**0.87**	0	0	0
7	0	0	0	0	0	0	0	0	0	**0.87**	0	**0.87**	0
8	0	0	0	0	0	0	0	0	0	0	0	**0.64**	0
9	0	0	0	0	0	0	0	0	0	0	0	**0.64**	0
10	0	0	0	0	0	0	**0.64**	0	0	0	**0.64**	0	0
11	0	0	0	0	0	0	**0.87**	0	**0.87**	**0.87**	0	0	0
12	0	0	**0.91**	0	0	0	**0.91**	0	0	0	0	0	0

Figure S13: **Computing the mapping matrix $\Im^{c\to m}$ and flyby segments $F_j^{D\to m}$ for cyclic global trajectories that toggle through multiple switch-attractors.** *Left*: normalized overlap of global states (rows) and switch-attractor states (columns). *Bold*: maximum overlap value(s) in each row. *Right*: Mapping matrix $\Im^{D\to m}$ recording these maximum overlap values (0 otherwise). *Colored segments*: flyby segments $F_j^{D\to m}$ for each visited attractor basin. Note that the turquoise segments represent a single $F_j^{D\to m}$ which loops from global state 10 back to 2.

Occasionally, more than one disjoint $F_j^{D\to m}$ flyby segment may exist for a single switch-m attractor Q_j^m (black segment on **Figs. S12D, S13**), meaning that the global system's trajectory attempts to replicate the jth switch-m phenotype more than once.

In order to identify the portion(s) of $F_j^{D\to m}$ that best mimic a switch-attractor Q_j^m, we reduce it to the *closest flyby segment* $\Gamma_j^{D\to m}$ by identifing the closest $s_k \in F_j^{D\to m}$ state(s) to each $z_l^{m\to j}$ switch-state along the switch limit cycle Q_j^m, using normalized overlap $O(s_k, z_l^{m\to j})$ to quantify similarity. We implement this by first picking the maxima in each *column* of the mapping matrix $\Im^{D\to m}$ (blue numbers on **Fig. S14**, left) and setting all other elements at the beginning and end of each $F_j^{D\to m}$ segment to 0. These flanking segments set to 0 correspond to the global trajectory $F_j^{D\to m}$ entering and leaving the Q_j^m basin, while the remaining (non-zero) continuous segment (or single state) between maxima represents the part of $F_j^{D\to m}$ which best replicates Q_j^m. This process results in the *closest flyby matrix* $\aleph^{D\to m}$ (**Fig. S14**, right). The remaining nonzero segments are the *closest flyby segments* $\Gamma_j^{D\to m}$ (colored boxes on **Figs. S14**).

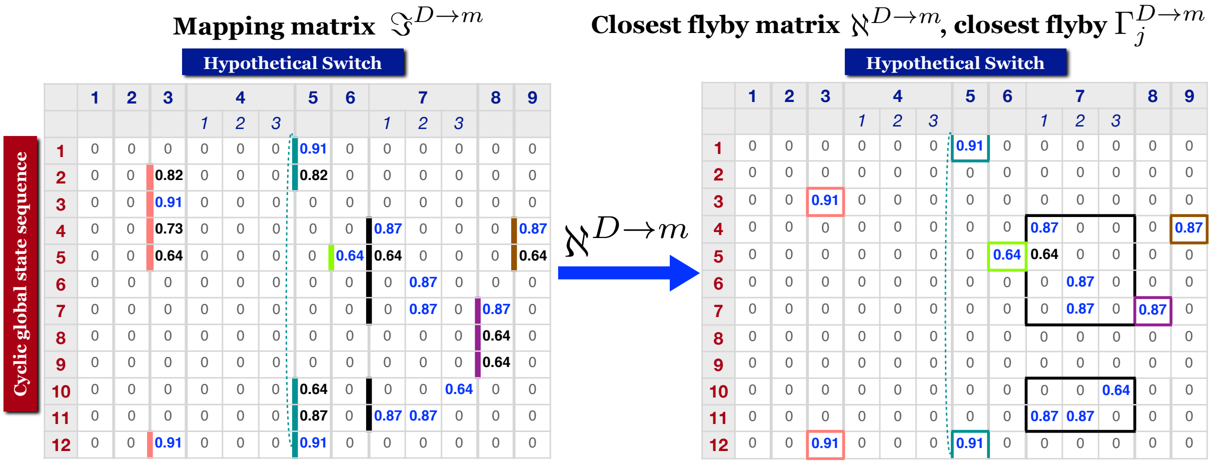

Figure S14: **Computing the closest flyby matrix $\aleph^{c\to m}$ and closest flyby segments $\Gamma_j^{D\to m}$ for cyclic global trajectories that toggle through multiple switch-attractors.** *Left*, Mapping Matrix $\Im^{D\to m}$. *Blue*: maximum overlap value(s) in each column. *Right*, Closest Flyby Matrix $\aleph^{D\to m}$ recording these maximum overlaps, as well as all values inside continuous flyby segments bracketed by these maxima (e.g., switch-attractor 7, column 1). *Colored boxes*: closest flyby segments $\Gamma_j^{D\to m}$ for each visited switch-attractor.

3.2 Overlap of closest flyby segment $\Gamma_j^{D\to m}$ and switch-attractor Q_j^m

We quantify the overlap of D^c with switch-m attractor Q_j^m based on how well its closest flyby segments $\Gamma_j^{D\to m}$ mimics the switch-phenotype represented by Q_j^m. When Q_j^m is a fixed-point (a static phenotype), this equals the overlap value associated with the state(s) of the closest flyby:

$$O(\Gamma_j^{D\to m}, Q_j^m) = O(s_k \in \Gamma_j^{D\to m}, z_1^{m\to j}).$$

When the switch-attractor Q_j^m is a limit-cycle, however, we expect a D^c segment with high overlap to Q_j^m to pass through (or at least approximate) every step of the closed Q_j^m loop. Specifically: **1)** We expect that the $F_j^{D\to m}$ flyby segment has very similar states to those of the switch-cycle. **2)** We expect the *order* of states that mimic each state of Q_j^m to be the same within the flyby sequence as in the switch-cycle (or at least very similar). According to this requirement, a global sequence composed of the same set of states as Q_j^m but running in reverse order should be classified as strongly dissimilar. **3)** We expect a flyby segment that accurately completes a *portion* of a switch-cycle but never closes the loop to have low overlap. We translated these requirements into a single overlap measure via the following multi-step process (**Fig. S15**).

a. Similarity of states and step conservation along $\Gamma_j^{D\to m}$ versus Q_j^m. By definition, each state $s_k \in \Gamma_j^{D\to m}$ maps onto states along the same switch limit-cycle Q_j^m, denoted $J^{D\to m}(k)$. Each step $s_k \to s_{k+1}$ along $\Gamma_j^{D\to m}$ thus maps onto switch-cycle states $J^{D\to m}(k)$ and $J^{D\to m}(k+1)$. For a $\Gamma_j^{D\to m}$ segment that faithfully follows a switch limit cycle, we expect the state(s) in $J^{D\to m}(k+1)$ to directly follow those in $J^{D\to m}(k)$. To test this, we calculate the number of forward time-steps $\Delta^{p,r}(k \to k{+}1)$ between *each pair* of switch-m states $z_p^{m\to j} \to z_r^{m\to j}$, where $z_p^{m\to j} \in J^{D\to m}(k)$ and $z_r^{m\to j} \in J^{D\to m}(k{+}1)$:

$$\Delta^{p,r}(k \to k+1) = \begin{cases} 1 & \text{if} \quad L_j^m = 1 \\ r - p & \text{if} \quad r \geq p, L_j^m \neq 1 \\ r - p + L_j^m & \text{if} \quad r < p, L_j^m \neq 1 \end{cases},$$

where L_j^m is the length of the switch limit-cycle Q_j^m. If s_k and s_{k+1} map onto two consecutive states along the switch-cycle, then $\Delta = 1$ (ideal case). If s_k and s_{k+1} map onto the same switch-state, $\Delta = 0$ (assuming $L_j^m > 1$). In the worst case, s_k maps onto a state immediately *after* the state $s_{k+1}^{c\to i}$ maps onto, thus reversing the direction of the cycle. The distance between these *along* the limit cycle is $\Delta = L_j^m - 1$. When the switch-attractor is a fixed-point ($L_j^m = 1$), $s_k^{c\to i}$ and $s_{k+1}^{c\to i}$ mapping onto it complete the 1-state cycle, thus $\Delta = 1$.

Taken together, the normalized overlaps $O(s_k, z_p^{m\to j})$, $O(s_{k+1}, z_r^{m\to j})$ and the value of $\Delta^{p,r}(k \to k+1)$ characterize the extent to which the $s_k \to s_{k+1}$ step of $\Gamma_j^{D\to m}$ follows the switch limit cycle (**Fig. S15A**). In defining the overlap between this

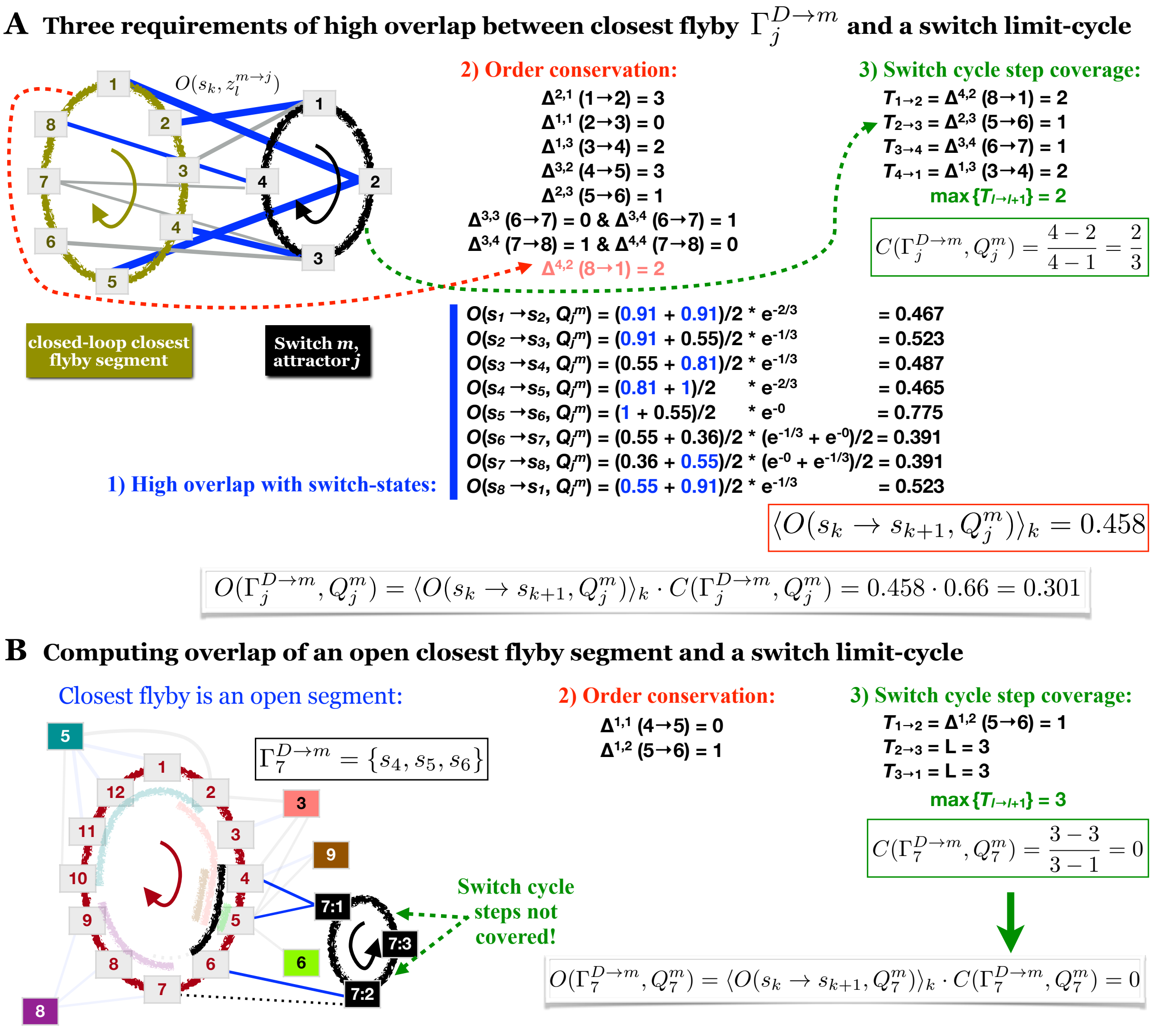

Figure S15: **Overlap between a closest flyby segment and a cyclic switch-attractor.** **(A)** *Left*, overlap between a global 8-state cycle (mustard) that never leaves the basin of a single cyclic switch-attractor (black). Black/blue links run from each global cycle state to the switch-cycle state(s) they best overlap (blue marks the highest-overlap links per *switch* cycle state). *Middle*, $\Delta(k \to k+1)$ denotes the number of forward time-steps between switch-states corresponding to each global cycle transition $k \to k+1$. $O(s_k \to s_{k+1})$ values are computed as products between the average overlap along the links originating from global states s_k and s_{k+1}, and $\exp\left(-\frac{|\Delta(k\to k+1)-1|}{L_j^m - 1}\right)$, where L_j^m is the switch-attractor's length. *Top right*, for each switch-cycle transition, $T_{l\to l+1}$ represents the minimum value of Δ among all global state transitions $k \to k+1$ that map to switch-cycle states which bracket the $l \to l+1$ transition, in that the shortest path along the switch-cycle from the state matching k to the one matching $k+1$ includes the $l \to l+1$ transition. The maximum of T along the switch-cycle is used to compute the *coverage* $C(\Gamma_j^{D\to m}, Q_j^m)$. *Bottom*, overlap $O(\Gamma_j^{D\to m}, Q_j^m)$ between a closest flyby segment and a switch limit cycle is a product of the average of overlaps along the global limit cycle, and the switch-cycle coverage. **(B)** Overlap between an open segment (black) along a global 12-state cyclic trajectory (red) that enters, then leaves the basin of a cyclic switch-attractor (black). The $\Gamma_7^{D\to m}$ closest flyby segment passed through switch cycle transition $1 \to 2$, but fails to implement its other two steps. As a consequence the coverage of this switch cycle is 0.

step and Q_j^m, we exponentially punish the reversal of steps along the cycle:

$$O(s_k \to s_{k+1}, Q_j^m) = \Bigg\langle \frac{O(s_k, z_p^{m\to j}) + O(s_{k+1}, z_r^{m\to j})}{2} \cdot \exp\left(-\frac{|\Delta^{p,r}(k\to k+1) - 1|}{L_j^m - 1}\right)\Bigg\rangle_{z_p^{m\to j}\in J^{D\to m}(k),\; z_r^{m\to j}\in J^{D\to m}(k+1)}.$$

Next, we compute the *average* overlap along $\Gamma_j^{D\to m}$ as $\langle O(s_k \to s_{k+1}, Q_j^m)\rangle_k$. Here we distinguish two cases. First, it is possible that the the global dynamic trajectory is a closed loop (a global cyclic attractor) that maps onto a single switch-attractor j in its entirety (**Fig. S15A**, left). Thus, $F_j^{D\to m} = \Gamma_j^{D\to m} = Q_i^c$. In this case, the global dynamics "inherited" the cyclic dynamics of the switch m. Consequently, we treat $\Gamma_j^{D\to m}$ as a closed loop and calculate Δ and overlap not only for all $k \to k+1$ transitions ($k = \overline{1, L_i^c}$), but also for the $L_i^c \to 1$ transition, thus including $O(s_{L_i^c} \to s_1, Q_j^m)$ in the average (**Fig. S15A**, step marked in pink). Alternatively, if the global trajectory D^c approaches switch-m attractor Q_j^m, spends a continuous segment $\Gamma_j^{D\to m}$ in its basin, then leaves it again, then $\Gamma_j^{D\to m}$ is as an open segment, for which we only calculate Δ and overlap for $k \to k+1$ transitions (**Fig. S15B**, black segment with transitions $4 \to 5$ and $5 \to 6$, but not $6 \to 4$).

b. Coverage of Q_j^m by the closest flyby segment $\Gamma_j^{D\to m}$. Next, we measure how closely each step of the switch limit-cycle Q_j^m is mimicked by $\Gamma_j^{D\to m}$. To this end, we calculate

$$T(l \to l+1) = \begin{cases} \min\limits_k\{\Delta^{p,r}(k \to k+1) \mid (z_l^{m\to j} \to z_{l+1}^{m\to j}) \in (z_p^{m\to j} \to z_r^{m\to j})\} & \text{if } \exists\, k \\ L_j^m & \text{otherwise} \end{cases},$$

where $z_p^{m\to j} \in J^{D\to m}(k)$ and $z_r^{m\to j} \in J^{D\to m}(k+1)$. $T(l \to l+1)$ represents the minimum Δ among all $s_k \to s_{k+1}$ transitions that map onto switch-cycle states which include the $z_l^{m\to j} \to z_{l+1}^{m\to j}$ step in their transition path (**Fig. S15**). If no such $s_k \to s_{k+1}$ transition exists, $T(l \to l+1) = L_j^m$ (**Fig. S15B**, $l = \{2,3\}$). An intuitive example for this case is a flyby segment $\Gamma_j^{D\to m}$ that goes very close to *one* state in a two-state switch-cycle, but none of its elements maps onto the other state. In this case $T(1 \to 2) = T(2 \to 1) = 2$, a scenario for which we expect coverage score 0 (also see **Fig. S15B**).

Ideal coverage, on the other hand, requires *all* steps of the switch-cycle Q_j^m to be covered by individual steps along $\Gamma_j^{D\to m}$ (i.e., $T(l \to l+1) = 1$ for each l). Thus we define the *coverage score* $C(\Gamma_j^{D\to m}, Q_j^m)$ using the *largest* $T(l \to l+1)$ along the cycle, as:

$$C(\Gamma_j^{D\to m}, Q_j^m) = \begin{cases} \frac{L_j^m - \max\limits_l\{T(l\to l+1)\}}{L_j^m - 1} & \text{if } L_j^m > 1 \\ 1 & \text{if } L_j^m = 1 \end{cases}.$$

In summary, perfect traversal of all steps along Q_j^m by $\Gamma_j^{D\to m}$ results in $C(\Gamma_j^{D\to m}, Q_j^m) = 1$, while completely omitting one of its steps gives $C(\Gamma_j^{D\to m}, Q_j^m) = 0$. Putting together the measures devised for the three requirements, we define $O(\Gamma_j^{D\to m}, Q_j^m)$ as:

$$O(\Gamma_j^{D\to m}, Q_j^m) = \big\langle O(s_k \to s_{k+1}, Q_j^m)\big\rangle_k \cdot C(\Gamma_j^{D\to m}, Q_j^m).$$

3.3 Overlap of global attractor Q_i^m and switch-attractor Q_j^m

Leveraging the formula for $O(\Gamma_j^{D\to m}, Q_j^m)$, we are now ready to define an overlap measure between an arbitrary global attractor Q_i^c and an arbitrary switch attractor Q_j^m as:

$$O(Q_i^c, Q_j^m) = \langle O(\Gamma_j^{D\to m}, Q_j^m)\rangle_{\text{distinct } \Gamma_j^{D\to m} \text{ flyby segments}},$$

where the global state segment, $D = Q_i^c$, represents the ith global attractor.

4 Randomization of multi-switch Boolean regulatory models

4.1 Creating randomized multi-switch networks

In order to test whether randomized versions of our Cell Cycle models show markedly different characteristics from the biological system, we used three distinct sets of randomization procedures.

1. **Complete link and gate randomization, with the exception of self-sustaining input nodes**

- Generate completely random directed networks with $N - i$ nodes, where i is the number of 1-node input switches (self-sustaining input nodes) in the original model. For the *p21*-null cell cycle model $i = 1$, while the model with a $p21_{TR}$ input switch has $i = 2$ such nodes. Each random network has $K - \sum_{j=1}^{i} k_j$ links, where k_j represent the number of outgoing links from 1-node switch j. These nodes and their outgoing links will be reattached to the random network, as described below.
- Rewire the random network until every node is reachable through at least one directed path from every other nodes (no input/leaf nodes). This requirement allows all nodes to contribute to the existence and robustness of the system's attractors, as they do in the biological model. As long as the original model satisfies this requirement, there are a sufficient number of links in the random networks to satisfy it as well. We achieve this by an iterative process:
  - identify all input nodes, all leaf nodes, and all "spare" links which originate with nodes with more than one non-self out-link and end in nodes with more than one non-self in-link;
  - for each leaf node we remove a randomly chosen "spare" link and add back a connection from the leaf node to a random node;
  - for each input node we remove a randomly chosen "spare" link and add back a connection from a random node to the input node;
  - check if all nodes are reachable from all others via directed paths; if not, repeat.
- Assign a random Boolean gate to each node, with a probability p_0 of 0 outputs equal to the fraction of 0's among all outputs of all Boolean gates in the original model.
- Guarantee that each link of the random network is functional. Specifically, all inputs of each randomly generated Boolean gate must have an effect on the functions outcome for at least one combination of all other input-values. This requirement guarantees that none of the network's links are completely irrelevant to its dynamics. Whenever a randomly generated Boolean gate fails to satisfy this for an input link, we choose a random combination of all other input-values and flip one of the outputs that corresponds to it. We thus guarantee that in the presence of this particular input-combination, the gate becomes sensitive to the originally irrelevant link.
- For each switch of the biological system with more than 1 node, randomly assign a subset of N_m nodes to random switch m.
- Add all 1-node switches of the original system back to the random network, preserving the input status and self-link of switch-nodes (e.g., GF or *p21*$_{TR}$) and linking the rest of their outgoing connections to randomly chosen nodes of the network, with randomly chosen influence.

2. **Switch assignment randomization.** Here we randomize the assignment of nodes into switches *without* altering the global connectivity or dynamics of the system (the *GF*, *p21*$_{TR}$, *Replication*, *4N_DNA* and *Metaphase* nodes were not included in the randomization). The resulting random switches (and their isolated phenotypes) are thus different in every random network, but the coupled, global dynamics always matched that of the Cell Cycle model(s). Our initial switch memberships within the Cell Cycle Model were based on biological insight about the pathways that are critical to switch-like, bistable transitions within the cell cycle. In order to verify that this choice is indeed optimal, we randomly assign every node of the *Restriction* and *Phase Switches* to two random switches that match their original sizes.

3. **Randomized links between Cell Cycle switches.** Here we randomize the links connecting the *Restriction* and *Phase Switches* without altering their internal connectivity (or dynamics). All nodes of the *Restriction* and *Phase Switch* switches retain their switch-assignment, but the origin of every inter-switch link is rewired to a new node within its switch of origin. As a result, the coupled global dynamics is different in every random network, but the switches in isolation always match those of the Cell Cycle model(s). In addition, we randomize the combinatorial influence of the newly selected inputs:

   - For a node with k rewired, inter-switch inputs $\{i_1, i_2, \ldots, i_k\}$, a select input-value combination $\{v(i_1), v(i_2), \ldots, v(i_k)\}$ is identified such that the remaining intra-switch inputs have maximum influence over node output (see the following subsection a detailed description).
   - For each input i_1 (considered sequentially), half of the node-s outputs correspond to input-value $v(i_l)$, the other half to $1 - v(i_l)$. In order to randomize the influence of node i_l when it is *not* set to $v(i_l)$, this influence is first erased. To this end, a new Boolean gate is generated in which outputs do not depend on the value of i_1, but always match the old gate's $v(i_l)$-value outputs instead. The number of remaining input combinations for which a difference existed (and was now erased) is denoted by n_l .

- A randomized influence for input i_l is reintroduced by selecting n_l random value combinations among the remaining inputs, and flipping the output corresponding to this input-value combination and the value $1 - v(i_l)$ for input node i_l.

4.2 Uncoupling multi-switch Boolean networks into arbitrary isolated switches

In order to work with the randomized versions of the cell cycle network described above, we need general rules for uncoupling a network into arbitrarily chosen, disconnected switches. In addition to removing all links between switches, we need to define the process by which Boolean gates lose some of their inputs while preserving the dynamics driven by the remaining intra-switch inputs (as closely as possible).

Every time a subset of inputs are removed from a Boolean gate, we need to assume that the removed nodes are frozen into either an ON or an OFF state. Whenever k inputs are removed, there are 2^k possible combinations in which these removed inputs may be frozen, and the behavior of the gate in response to its remaining inputs may be different in each case. The question is, *which* one of these 2^k possible "partial" gates should we use? We wish to preserve as many of the remaining intra-switch regulatory influences as possible. To this end, for every combination of input values among the k links marked for removal, we calculate the entropy of the Boolean gate fragment left when their inputs are fixed (i.e., the array of 0/1 outputs the gate returns for every remaining input combination). The entropy of a Boolean gate is defined as $H_G = -p \cdot \log(p) - (1-p) \cdot \log(1-p)$, where p is the fraction of input combinations for which the gate returns 0. In addition to this entropy, we also test whether all remaining inputs of this gate fragment are functional (i.e., there exists at least one value combination among the other intra-switch links for which the input in question dictates the output). We then choose the gate fragment with the highest entropy among the ones that preserve the function of all remaining intra-switch links as the new, reduced gate. If no such gate fragment exists, we simply choose the largest entropy fragment (in case of two equivalent choices, we default to the first one found). The input-value combination among the removed gates that generated the chosen gate fragment will be referred to the *non-canalizing inter-switch inputs*.

Supplementary Table S1. The Restriction Switch

Node	Gate	In-link	Direct	Description & References
**CyclinD1**		***E2F1*** →	Yes	The *Cyclin D1* promoter is bound by *E2F* factors including *E2F1* [45], and *E2F1* overexpression can increase *Cyclin D1* (though its effects are context-dependent, as *E2F1* overexpression can also lead to apoptosis) [45]. Dominant negative *E2F1* overexpression results in a 2-3 fold decrease in *Cyclin D* expression and *Cyclin D/Cdk4,6* activity [35].
		Myc →	No	*Myc* overexpression leads to rapid *Cyclin D1* induction and subsequent cell cycle entry [46], while its absence halves *Cyclin D1* levels [47]. In addition, *Myc* induces *Cdk4*,. aiding the assembly of active *Cyclin D1/Cdk4,6* complexes [48, 47]
		CyclinD1 →	No	In order to take into account both production and stability of *Cyclin D1*, we assumed that the presence of active *CyclinD/Cdk2,4* complexes renders transcriptional maintenance of their levels easier.
	(***Myc*** AND ***E2F1***) OR [***CyclinD1*** AND (***Myc*** OR ***E2F1***)]			The precise regulatory logic of *Cyclin D1* as a function of *Myc* and *E2F* is not known. Here we assume that in the absence of active *Cyclin D1*, both Myc AND *E2F* are required to turn it ON [49]. In contrast, when *Cyclin D1* is already present, either *Myc* OR *E2F* can maintain above-threshold *Cyclin D1/Cdk2,4* activity.
**CyclinE**		***E2F1*** →	Yes	*E2F1* is a potent transcriptional activator of *Cyclin E* [50].
		RB ⊣	No	*Cyclin E* transcription by *E2F1* requires the absence of active, un-phosphorylated *RB* [51].
		p27Kip1 ⊣	Yes	*p27Kip1* binds to and prevents the activation of *Cyclin E/Cdk2* complexes [52].
	E2F1 AND (NOT ***RB***) AND (NOT ***p27Kip1***)			The *CyclinE* node represents active *Cyclin E/Cdk2* complexes, active when transcribed by *E2F1* in the absence of both *RB* (which blocks transcription) and *p27Kip1* (which blocks its activity) [51].
**E2F1**		***RB*** ⊣	Yes	*RB* binds to *E2F/DP1* complexes and switches their DNA binding activity from activation to repression [53, 54].
		E2F1 →	Yes	*E2F1* binds to its own promoter and up regulates transcription (as long as *Cyclin D/E* activity blocks *RB-E2F1* binding) [55].
		Myc →	Yes	*Myc* is required for growth-factor mediated induction of *E2F1* [56, 47]. It binds to and remodels the E2F1 promoter, facilitating *E2F1* transcription [49]. In addition, *Myc* augments protein expression of *E2F1* [57]. Single-cell experiments show that *Myc* is a critical modulator of the amplitude of *E2F* activation [30].
	(NOT ***RB***) AND (***E2F1*** OR ***Myc***)			The precise nature of the regulatory logic by which *E2F1* and *Myc* drive *E2F1* transcription is not known. Here we assume that in the absence of *RB*, either active *E2F1* OR *Myc* can induce *E2F1* transcription [30, 31, 32]. When *RB* is present, *E2F1* is inactive [53, 54].
**Myc**		***E2F1*** →	Yes	*E2F1* binds and activates the *c-Myc* promoter [34, 33, 58, 59].
	E2F1			*E2F1* activates *Myc* transcription.
**p27Kip1**		***CyclinD1*** ⊣	Yes	Active *Cyclin D/Cdk4,6* complexes competitively bind to *p27Kip1* and progressively inhibit its ability to keep *Cyclin-E/Cdk2* inactive, thereby inducing cdk2 activity and cell-cycle progression [52].
**p27Kip1**		***CyclinE*** ⊣	Yes	Active *Cyclin-E/Cdk2* phosphorylate *p27Kip1* at threonine 187 (Thr187) [60], which marks it for degradation by the SCF^{SKP2} complex at the onset of S-phase [61]. (*Cyclin-E/Cdk2* complexes remain active in the presence of *p27Kip1* and promote its degradation when *Cyclin-A* is also active, included in the multi-switch model – *Suppl. Table S3*.)
	NOT (***CyclinD1*** OR ***CyclinE***)			Either *CyclinD1* (representing active *Cyclin D/Cdk4,6* complexes) or *CyclinE* (representing active *Cyclin E/Cdk2* complexes) can inhibit *p27Kip1* activity.
**RB**		***CyclinD1*** ⊣	Yes	*Cyclin D1/Cdk4,6* complexes bind and phosphorylate *RB*, inhibiting its activity [62, 63, 64].
		CyclinE ⊣	Yes	*Cyclin E/Cdk2* complexes bind and phosphorylate *RB*, inhibiting its activity [65, 64].
		p27Kip1 →	No	Active *p27Kip1* can counteract the inhibitory effects of active *CyclinE/Cdk2* complexes [52].
	(NOT ***CyclinD1***) AND [(NOT ***CyclinE***) OR ***p27Kip1***]			*RB* is active in the absence of *CyclinD1* and *CyclinE*, and can maintain activity when active *p27Kip1* is present to counteract *CyclinE* [52].

Supplementary Table S2. The Phase Switch

Node	Gate	In-link	Direct	Description & References
Cdc20		***Cdh1*** ⊣	Yes	*APC/C*^{Cdh1} complexes degrade *Cdc20*, leading to a complete switch from *APC/C*^{Cdc20} to *APC/C*^{Cdh1} during mitotic exit [66, 67].
		Mad2 ⊣	Yes	Eukaryotic cells do not separate their replicated genome until they pass the Spindle Assembly Checkpoint (SAC). Namely, all their chromosomes need to be are aligned with respect to the metaphase plane and the two copies of each chromosome need to be attached to opposite poles of the mitotic spindle [68]. This physical alignment is monitored via *Mad2*: kinetochores that remain unattached to microtubules catalyze the sequestration of *Cdc20* and thus inhibit *APC/C*^{Cdc20} [69, 70].
		pAPC →	Yes	*Cdc20* becomes active in early mitosis by binding to *APC/C*, an event that requires *Cyclin B/Cdk1*-mediated phosphorylation of several core *APC/C* subunits [66, 71].
	pAPC AND (NOT ***Cdh1***) AND (NOT ***Mad2***)			In our model, *APC/C*^{Cdh1} complex formation is represented by the joint presence of an active *Cdc20* node, together with phosphorylated *APC/C*, labeled *pAPC*. *Cdc20* is thus ON when both *Cdh1* and *Mad2* are absent, and *pAPC* is ON (present and phosphorylated). (*APC/C*^{Cdh1} complex formation was represented by the *Cdh1* node alone, described below.)
Cdc25A		***Cdh1*** ⊣	Yes	The *APC/C*^{Cdh1} complex degrades *Cdc25A* at mitotic exit [72, 66].
		CyclinA →	Yes	*Cdc25A* protein levels are stabilized during S and G2 by *Cdk2*-dependent phosphorylation [73]. During the cell cycle, this requires *Cdc25A* transcription by *E2F1* and activation by *Cyclin E/Cdk2* (Supplementary Table S3). Here we assume that once *Cdc25A* transcription stops in G2 (*Cyclin A* deactivates *E2F1*), the presence of active *Cyclin A/Cdk1* complexes can still maintain *Cdc25A* activity [74].
	CyclinA AND (NOT ***Cdh1***)			In the absence of *Cdh1* (*APC/C*^{Cdh1}), *Cdc25A* is kept ON by *CyclinA*.
Cdc25C		***CyclinA*** →	Yes	*Cyclin A/Cdk1* complexes can activate *Cdc25C* [75].
		CyclinB →	Yes	*Cyclin B/Cdk1* complexes are potent activators of *Cdc25C*, forming a positive feedback loop that causes switch-like mitotic entry [76, 75].
Cdc25C		***Cdk1*** →	Yes	*Cyclin B/Cdk1* complexes are potent activators of *Cdc25C* [76, 75].
	CyclinA OR (***CyclinB*** AND ***Cdk1***)			*Cdc25C* is active in the presence of *Cyclin A,B/Cdk1* complexes.
Cdh1		***CyclinA*** ⊣	Yes	Active *Cyclin A/Cdk1,2* complexes phosphorylate *Cdh1* during S, G2 and early mitosis, impairing its interaction with *APC/C* until late stages of mitosis when *Cdk1/2* activity falls [66, 77].
		CyclinB ⊣	Yes	*Cyclin B/Cdk1* phosphorylates *Cdh1* during mitosis, impairing its interaction with *APC/C* [66, 77].
		Cdk1 ⊣	Yes	*Cyclin B/Cdk1* phosphorylates *Cdh1* during mitosis, impairing its interaction with *APC/C* [66, 77].
	(NOT ***CyclinA***) AND [NOT (***CyclinB*** AND ***Cdk1***)]			*Cdh1* activity requires the absence of active *Cyclin A* and *Cyclin B/Cdk1*.
Cdk1		***Cdc25C*** →	Yes	*Cdk1* is subject to inhibitory phosphorylation by *Wee1* or *Myt1*, and its dephosphorylation is carried out by activated *Cdc25C* [76, 78, 75].
		CyclinA →	Yes	In order to carry kinase activity, *Cdk1* must form a complex with *Cyclin A* or *Cyclin B* [78].
		CyclinB →	Yes	In order to carry kinase activity, *Cdk1* must form a complex with *Cyclin A* or *Cyclin B* [78].
		Wee1 ⊣	Yes	*Wee1* is a nuclear protein that ensures the completion of DNA replication prior to mitosis by blocking nuclear *Cdk1* activation. Thus, *Cdk1* is likely to be activated in the cytoplasm and requires nuclear localization to initiate both cytoplasmic and nuclear mitotic transformations. The human wee1 kinase appears to coordinate the transition between DNA replication and mitosis by protecting the nucleus from cytoplasmic *Cdk1* kinase [79].
		Cdk1 →	Yes	We assume that the presence of fully activated, nuclear *Cdk1* is able to overcome the effect of active *Wee1*, given that *Wee1* is very sensitive to *Cdk1*-mediated inhibitory phosphorylation [80].
	Cdc25C AND (***CyclinA*** OR ***CyclinB***) AND [***Cdk1*** OR (NOT ***Wee1***)]			Full *Cdk1* activity requires the presence of *Cdc25C*, one of its cyclin partners (*A* or *B*), and either the absence of *Wee1*, or sustained presence of *Cdk1* itself.

**CyclinA**	***Cdc25A*** →	Yes	*Cdc25A* promotes active *Cyclin A/Cdk2* complex formation by removing inhibitory phosphorylation of *Cdk2* [81, 82].
	CyclinA →	Yes	We assume that once activated, *Cyclin A/Cdk2,1* complexes can sustain their activity until *Cyclin A* is degraded.
	pAPC ⊣	Yes	*Cyclin A* is degraded by *APC/C*^{Cdc20} [83].
	Cdc20 ⊣	Yes	*Cyclin A* is degraded by *APC/C*^{Cdc20} [83].
	Cdh1 ⊣	Yes	*Cyclin A* is degraded by *APC/C*^{Cdh1} in the presence of the *UbcH10* protein [77, 84, 17].
	UbcH10 ⊣	Yes	*Cyclin A* degradation by *APC/C*^{Cdh1} requires *UbcH10* [84].
	(***Cdc25A*** OR ***CyclinA***) AND {NOT [(***pAPC*** AND ***Cdc20***) OR (***Cdh1*** AND ***UbcH10***)] }		*Cyclin A* activity may be induced by active *Cdc25A*, promoting the formation of active *Cyclin A/Cdk2* complexes [81, 82], or maintained in the absence of *Cdc25A* by already active *Cyclin A* . *Cyclin A* is degraded by *APC/C*^{Cdc20} (represented in the model as [*pAPC* AND *Cdc20*]), or *APC/C*^{Cdh1} (represented by *Cdh1*) in the presence of the *UbcH10* protein [77, 84, 17].
**CyclinB**	***pAPC*** ⊣	Yes	*Cyclin B* is degraded by *APC/C*^{Cdc20} [77].
	Cdc20 ⊣	Yes	*Cyclin B* is degraded by *APC/C*^{Cdc20} [77].
	Cdh1 ⊣	Yes	*Cyclin B* is degraded by *APC/C*^{Cdh1} [77].
	NOT (***pAPC*** AND ***Cdc20***) AND (NOT ***Cdh1***)		In contrast to the *CyclinE* and *CyclinA* nodes, which represent active cyclin/Cdk complexes, the *Cyclin B* node is ON when the concentration of *Cyclin B* proteins is high, without any assumption about the activity of *CyclinB/Cdk1* complexes (consequently, downstream effects of *CyclinB/Cdk1* require [*CyclinB* AND *Cdk1*]). *Cyclin B* is degraded by *APC/C*^{Cdc20} or *APC/C*^{Cdh1} [77], and it accumulates in their absence.
**Mad2**	***pAPC*** ⊣	Yes	Multi-ubiquitination by *APC/C*^{Cdc20} leads to the dissociation of *Mad2* from *Cdc20*, abrogating the role of *Mad2* in maintaining the SAC [69].
	Cdc20 ⊣	Yes	Multi-ubiquitination by *APC/C*^{Cdc20} leads to the dissociation of *Mad2* from *Cdc20*, abrogating the role of *Mad2* in maintaining the SAC [69].
	CyclinB →	No	At the start of mitosis (marked by activation of *Cyclin B/Cdk1* complexes), *Mad2* is recruited to unattached kinetochores to prevent these misaligned sister chromatids from separating, thus establishing and maintaining the SAC [85, 86].
	Cdk1 →	No	At the start of mitosis *Mad2* is recruited to unattached kinetochores to prevent these misaligned sister chromatids from separating, thus establishing and maintaining the SAC [85, 86].
	NOT (***pAPC*** AND ***Cdc20***) AND ***CyclinB*** AND ***Cdk1***		*Mad2* is ON in metaphase, when *CyclinB* and *Cdk1* is active, and *APC/C*^{Cdc20} (*pAPC* AND *Cdc20*) is inactive.
**pAPC**	***pAPC*** →	Yes	Activated *APC/C*^{Cdc20} initiates the Metaphase → Anaphase transition by degrading *Cyclin B* and *securing* [87, 68]. Once active, *APC/C*^{Cdc20} no longer requires sustained *CyclinB/Cdk1* phosphorylation.
	Cdc20 →	Yes	Once active, *APC/C*^{Cdc20} no longer requires sustained *CyclinB/Cdk1* phosphorylation.
	CyclinB →	Yes	*CyclinB/Cdk1* activation triggers mitotic entry and promotes *APC/C*^{Cdc20} activity via APC/C subunit phosphorylation [88, 68].
	Cdk1 →	Yes	*CyclinB/Cdk1* activation triggers mitotic entry and promotes *APC/C*^{Cdc20} activity via APC/C subunit phosphorylation [88, 68].
	(***pAPC*** AND ***Cdc20***) OR (***CyclinB*** AND ***Cdk1***)		*pAPC* is ON when *APC/C* is phosphorylated: if *CyclinB* AND *Cdk1* are ON, or if *APC/C*^{Cdc20} is already active.
**UbcH10**	***Cdh1*** ⊣	Yes	*UbcH10* is degraded by *APC/C*^{Cdh1}.
	Cdc20 →	Yes	The presence of *APC/C*^{Cdh1} substrates, including *Cdc20*, inhibit the autoubiquitination of *UbcH10* but not its function, thus preserving APC activity [84].
	CyclinA →	Yes	The presence of *APC/C*^{Cdh1} substrates, including *Cyclin A*, inhibit the autoubiquitination of *UbcH10* but not its function, thus preserving APC activity [84].
**UbcH10**	***CyclinB*** →	Yes	The presence of *APC/C*^{Cdh1} substrates, including *CyclinB*, inhibit the autoubiquitination of *UbcH10* but not its function, thus preserving APC activity [84].
	UbcH10 →	Yes	Active *UbcH10* cannot be autoubiquitinated in the presence of *APC/C*^{Cdh1} substrates and thus remains active [84].
	(NOT ***Cdh1***) OR [***UbcH10*** AND (***Cdc20*** OR ***CyclinA*** OR ***CyclinB***)]		The ubiquitin-conjugating enzyme (E2) *UbcH10* is active in the absence of *Cdh1*. Alternatively, active *UbcH10* is maintained in the presence of *Cdh1* when some of its targets are present: *Cdc20* OR *CyclinA* OR *CyclinB* [84].

**Wee1**		***Cdk1*** $\dashv$	Yes	The somatic *Wee1* protein is an order of magnitude more sensitive to *Cdk1* activity than *Cdc25C*. Thus, both *Cyclin A/Cdk1* and *Cyclin B/Cdk1* strongly induce *Wee1* phosphorylation and deactivation [80].
		CyclinA $\dashv$	Yes	*Cyclin A/Cdk1* is a strong inducer of *Wee1* phosphorylation and deactivation [80].
		CyclinB $\dashv$	Yes	*Cyclin B/Cdk1* is a strong inducer of *Wee1* phosphorylation and deactivation [80].
	NOT [(***CyclinA*** OR ***CyclinB***) AND ***Cdk1***]			*CyclinA/Cdk1* OR *CyclinB/Cdk1* can block the activity of *Wee1* [80].

Supplementary Table S3. The full (p21-TR) Cell Cycle Model

Node	Gate	Ext. link	Direct	Description & References
**GF**		***GF*** $\to$	No	We assume a stable external presence ($GF = 1$) or absence ($GF = 0$) of growth factors.
	GF			We model the stability of the Cell Cycle Model's external environment by a self-activating GF node. Due to this self-loop, this node acts as switch – in the decoupled system's dynamics it gives rise to 2 attractors for every attractors combination of the other two switches (**Fig. 3A**).
**$p21_{TR}$**		***$p21_{TR}$*** $\to$	No	We assume a stable presence ($p21_{TR} = 1$) or absence ($p21_{TR} = 0$) of $p21$ transcription, which acts as an intracellular input node to the modeled cell cycle network.
	$p21_{TR}$			Due to this self-loop, this node also acts as switch: in the decoupled system's dynamics it gives rise to 2 attractors for every attractors combination of the other three switches. When set to $p21_{TR} = 0$, the dynamics of this model is equivalent to the $p21$-null model presented in the first part of the paper (**Figs. 2** to **5**).
**CyclinD1**		***Replication*** $\dashv$	No	During replication, checkpoint kinases suppress *Cyclin D1* [89], which has a very short half- life ($\sim$ 24 min) [90].
		GF $\to$	No	Extracellular growth signals activate the MAPK pathway, leading to transcriptional activation of *Cyclin D*, stabilization of its mRNA levels, and formation of active *Cyclin D/Cdk4,6* complexes [91, 92].
		p21-B $\dashv$	Yes	*p21*^{Cip1} is a cyclin-dependent kinase (Cdk) inhibitor which binds to and blocks the activity of *Cdk2*, *Cdk3*, *Cdk4* and *Cdk6* kinases [93], and thus inhibits *CyclinD1/Cdk4,6* [94].
**CyclinD1**	(NOT ***Replication***) AND ⟨[***GF*** AND (NOT ***p21-B***) AND (***Myc*** OR ***E2F1***)] OR [***Myc*** AND ***E2F1*** AND {(NOT ***p21-B***) OR (***GF*** AND [***CyclinD1*** OR (NOT ***RB***)])}]⟩			Ongoing *Replication* inhibits *CyclinD1*. Under ideal conditions for its activation, namely when *GF* are present and *p21-B* is inactive, *CyclinD1* is transcribed by either *Myc* OR *E2F1*, and active. Under non-ideal conditions, we assume that both *Myc* AND *E2F1* are required for *CyclinD1* transcription; in addition, *CyclinD1* activity requires the absence of inhibition from *p21-B*, OR the presence of *GF* as well as already active *CyclinD1* or deactivated *RP*.
**CyclinE**		***Cdh1*** $\to$	No	The final step in the activation of nuclear *Cyclin E/Cdk2* kinase complexes involves chromatin association of *Cdk2* at pre-replication complexes (pre-RCs), mediated by the pre-RC protein *Cdc6* [95]. In addition to *Cdc6*, pre-RCs formation also requires *Cdt1*, kept inactive by the replication licensing inhibitor *geminin*. *Geminin*, in turn, is targeted for degradation by *APC/C*^{Cdh1} [68]. Thus, the ***Cdh1*** $\to$ ***CyclinE*** link in our model stands for *APC/C*^{Cdh1} $\dashv$ *geminin* $\dashv$ *Ctd1* $\to$ pre-RCs formation $\to$ *Cdc6* $\to$ *CyclinE/Cdk2*.
		Metaphase $\dashv$	No	In the absence of *APC/C*^{Cdh1} activity, *geminin* is targeted for degradation by *APC/C*^{Cdc20} at the SAC. As *Metaphase* $= ON$ in our model marks the window of full *Cyclin B/Cdk1* activity prior to SAC passage, and thus preceding *APC/C*^{Cdc20} activation, the ***Metaphase*** $\dashv$ ***CyclinE*** link in our model stands for *Metaphase* $\dashv$ *APC/C*^{Cdc20} $\dashv$ *geminin* $\dashv$ *Ctd1* $\to$ pre-RCs formation $\to$ *Cdc6* $\to$ *CyclinE*.
	[***Cdh1*** OR (NOT ***Metaphase***)] AND {***E2F1*** AND [NOT (***p27Kip1*** OR ***RB***)]}			The *CyclinE* node, representing active *CyclinE/Cdk2* complexes, is transcribed by *E2F1*. Unless inhibited by *p27Kip1* OR *RB*, *CyclinE* is fully activated by binding *Cdc6* at pre-RC complexes, present when *Cdh1* is active OR *Metaphase* has passed.
**E2F1**		***CyclinA*** $\dashv$	Yes	The phosphorylation of the *E2F1*-binding *DP-1* protein by *Cyclin A*, which binds directly to *E2F-1* (as well as *E2F-2,3*) downregulates *E2F* transcriptional activity in S phase [37, 96, 51].
	(NOT ***CyclinA*** OR ***RB***) AND (***E2F1*** OR ***Myc***)			In the absence of *CyclinA* [37, 96, 51] AND *RB* [53, 54], *E2F1* transcription can be induced by already active *E2F1* OR *Myc* [30, 31, 32].

**Myc**		*GF* →	No	Extracellular growth signals induce *c-Myc* transcription [97, 98, 92].
	GF OR *E2F1*			c-Myc is turned on by growth factor stimulation, and can be sustained in their absence by *E2F1*.
**p27Kip1**		*CyclinB* ⊣	Yes	*Cyclin B/Cdk1* complexes phosphorylate $p27^{Kip1}$ [99], and although they do not promote its degradation, phosphorylated $p27^{Kip1}$ is exported from the nuclear compartment and looses its ability to inhibit *Cdk* activity [100].
		Cdk1 ⊣	Yes	*Cyclin B/Cdk1* complexes phosphorylate $p27^{Kip1}$ [99], and although they do not promote its degradation, phosphorylated $p27^{Kip1}$ is exported from the nuclear compartment and looses its ability to inhibit *Cdk* activity [100].
		CyclinA ⊣	Yes	*Cyclin A/Cdk2* complexes bind and inactivate $p27^{Kip1}$ by sequestration, phosphorylate it, and promote its degradation [99].
	[NOT (*CyclinB* AND *Cdk1*)] AND {NOT [*CyclinD1* OR (*CyclinA* AND *CyclinE*)]}			$p27^{Kip1}$ is active in the absence of *Cyclin/Cdk* complexes. Active p27 is sequestered and inhibited by *Cyclin D/Cdk4,6*, OR by to binding to both *Cyclin E/Cdk2* AND *Cyclin A/Cdk2* (the inhibition is mutual; $p27^{Kip1}$ can block one, but not) [17, 64, 101].
**RB**		*CyclinA* ⊣	Yes	*Cyclin A/Cdk1,2* complexes phosphorylate and deactivate *RB* [102].
		CyclinB ⊣	Yes	*Cyclin B/Cdk1* deactivate *RB* [64, 17].
**RB**		*Cdk1* ⊣	Yes	*Cdk1* complexed with cyclins can deactivate *RB* [102, 64, 17].
	NOT (*CyclinB* AND *Cdk1*) AND (NOT *CyclinD1*) AND [*p27Kip1* OR NOT (*CyclinA* OR *CyclinE*)]			*RB* can be phosphorylated and deactivated by several *Cyclin/Cdk* combinations, including *cyclin D/Cdk4,6*, *cyclin E/Cdk2*, and *Cyclin A/Cdk1,2* [102, 64, 103]. *RB* can maintain its activity in the presence of *cyclin E* or *cyclin A* (but not both), provided their inhibitory activity is blocked by $p27^{Kip1}$ [101].
**Cdc20**	*pAPC* AND (NOT *Cdh1*) AND (NOT *Mad2*)			All inputs are internal to the ***Phase Switch*** (Supplementary Table S2).
**Cdc25A**		*E2F1* →	Yes	Deactivation of the retinoblastoma protein in late G1 leads to *E2F1*-mediated transcriptional activation of *Cdc25A* [104].
		CyclinE →	Yes	*Cdc25A* protein levels are stabilized during S-phase by *CyclinE/Cdk2*-dependent phosphorylation [73].
	[*E2F1* AND (*CyclinE* OR *CyclinA*)] OR [(NOT *Cdh1*) AND *CyclinE* AND *CyclinA*]			*Cdc25A* is stabilized during S-G2 by *Cdk2*-dependent phosphorylation [73]. This requires transcription by *E2F1* AND activation by *Cyclin E/Cdk2* OR *Cyclin A/Cdk2*. Alternatively, *Cdc25A* can stay active in the absence of *E2F1* if it is not degraded by APC/C^{Cdh1}, AND both *Cyclin E/Cdk2* AND *Cyclin A/Cdk2* are active [74].
**Cdc25C**	*CyclinA* OR (*CyclinB* AND *Cdk1*)			All inputs are internal to the ***Phase Switch*** (Supplementary Table S2).
**Cdh1**	[NOT (*CyclinB* AND *Cdk1*)] AND (NOT *CyclinA*)			All inputs are internal to the ***Phase Switch*** (Supplementary Table S2).
**Cdk1**	*Cdc25C* AND (*CyclinA* OR *CyclinB*) AND [*Cdk1* OR (NOT *Wee1*)]			All inputs are internal to the ***Phase Switch*** (Supplementary Table S2).
**CyclinA**		*E2F1* →	Yes	*Cyclin A* is transcriptionally activated by E2F factors [51].
	[(*E2F1* AND *Cdc25A*) OR *CyclinA*] AND {NOT [(*pAPC* AND *Cdc20*) OR (*Cdh1* AND *Ubc1*)] }			S-phase activation of *Cyclin A* requires transcription by *E2F* factors AND *Cdk2* activation by *Cdc25A* [51]. When already active, *Cyclin A* sustains its function in the absence of *APC/C*-mediated degradation (APC/C^{Cdc20}, OR APC/C^{Cdh1} bound to *UbcH10* [77, 84]).
**CyclinB**	NOT (*pAPC* AND *Cdc20*) AND (NOT *Cdh1*)			All inputs are internal to the ***Phase Switch*** (Supplementary Table S2).
**Mad2**		*Metaphase* ⊣	No	In our model, the *Metaphase* node turns ON when spindle assembly is completed, and none of the kinetochores are left unattached. At this point, *Mad2* is inactivated [69, 70].
	CyclinB AND *Cdk1* AND NOT [(*pAPC* AND *Cdc20*) OR *Metaphase*]			*Mad2* is ON during metaphase (i.e., *before* the *Metaphase* node turns ON, marking the completion of spindle assembly) when *CyclinB* and *Cdk1* is active, and APC/C^{Cdc20} (*pAPC* AND *Cdc20*) is inactive.
**pAPC**	(*pAPC* AND *Cdc20*) OR (*CyclinB* AND *Cdk1*)			All inputs are internal to the ***Phase Switch*** (Supplementary Table S2).
**UbcH10**	(NOT *Cdh1*) OR [*UbcH10* AND (*Cdc20* OR *CyclinA* OR *CyclinB*)]			All inputs are internal to the ***Phase Switch*** (Supplementary Table S2).
**Wee1**		*Replication* →	No	During DNA replication *Wee1* is activated by the checkpoint kinase *Chk1* [105, 106].

**Wee1**	*Replication* AND {NOT [(***CyclinA*** OR ***CyclinB***) AND ***Cdk1***]			*Wee1* is active during *Replication*, unless its activity is blocked by *CyclinA/Cdk1* OR *CyclinB/Cdk1* [80].
**Metaphase**		*4N DNA* →	No	Metaphase starts once replication is complete and cells have duplicated their DNA content (*4N DNA* $= ON$). Sister chromatids are held together by their own kinetochores, which face in opposing directions and attach to opposite poles of the mitotic spindle. These sister chromatids are aligned with respect to the metaphase plane, and the two copies of each chromosome become attached to opposite poles of the mitotic spindle [68]. The completion of this process is marked in our model by the *Metaphase* $= OFF \rightarrow ON$ transition [70], and passage of the SAC.
		CyclinB →	No	Activation of *CyclinB/Cdk1* marks the onset of *Metaphase*.
		Cdk1 →	No	Activation of *CyclinB/Cdk1* marks the onset of *Metaphase*.
		pAPC ⊣	No	SAC passage is marked by the activation of *APC/C*^{Cdc20}.
		Cdc20 ⊣	No	SAC passage is marked by the activation of *APC/C*^{Cdc20}.
	4N DNA AND ***CyclinB*** AND ***Cdk1*** AND [NOT(***pAPC*** AND ***Cdc20***)]			*Metaphase* is initiated by *CyclinB/Cdk1* complexes in cells with 4N DNA content (i.e., in G2 phase), and terminated by activation of *APC/C*^{Cdc20}.
**Replication**		***CyclinE*** →	No	DNA replication is initiated by fully active *CyclinE/Cdk2* [107].
		Cdc25A →	No	Active *Cdc25A* is required for onset as well as progression through S-phase [108, 109].
		4N DNA ⊣	No	Complete duplication of a cell's DNA, represented in our model by *4N DNA* $= ON$, marks the end of active *Replication*.
	CyclinE AND ***Cdc25A*** AND (NOT *4N DNA*)			*Replication* is ongoing in the presence of *CyclinE/Cdk2* AND *Cdc25A*, and turns off when cells double their DNA content (*4N DNA*).
**4N DNA**		***CyclinA*** →	No	*Cyclin A/Cdk1* complexes regulate the origin firing program in mammalian cells, and are required for the completion of DNA replication [82, 107, 83]
		Replication →	No	DNA content is doubled by the process of *Replication*.
		Cdh1 ⊣	No	*APC/C*^{Cdh1} reactivation occurs at exit from mitosis. In our model, *APC/C*^{Cdh1} activation marks the start of cytokinesis and the subsequent resetting of daughter cell DNA content to 2N [68].
		4N DNA →	No	Once achieved, a cell's *4N DNA* content is sustained up to the point of cytokinesis.
	(NOT ***Cdh1***) AND [*4N DNA* OR (***CyclinA*** AND ***Replication***)]			Cells achieve *4N DNA* content when *Replication* is completed by assistance from *Cyclin A*, and sustain it in until cytokinesis (marked by *Cdh1* activation).

References

[1] Teruel, M. N. & Meyer, T. Parallel single-cell monitoring of receptor-triggered membrane translocation of a calcium-sensing protein module. *Science (New York, NY)* **295**, 1910–1912 (2002).

[2] Madar, D. *et al.* Promoter activity dynamics in the lag phase of Escherichia coli. *BMC systems biology* **7**, 136 (2013).

[3] Yin, Z. *et al.* A screen for morphological complexity identifies regulators of switch-like transitions between discrete cell shapes. *Nat Cell Biol* **15**, 860–871 (2013).

[4] Wang, R.-S., Saadatpour, A. & Albert, R. Boolean modeling in systems biology: an overview of methodology and applications. *Physical biology* **9**, 055001 (2012).

[5] Karlebach, G. & Shamir, R. Modelling and analysis of gene regulatory networks. *Nature Reviews Molecular Cell Biology* **9**, 770–780 (2008).

[6] De Jong, H. Modeling and simulation of genetic regulatory systems: A literature review. *J Comput Biol* **9**, 67–103 (2002).

[7] Laub, M. T. & Loomis, W. F. A molecular network that produces spontaneous oscillations in excitable cells of Dictyostelium. *Mol Biol Cell* **9**, 3521–3532 (1998).

[8] Ma, L. & Iglesias, P. A. Quantifying robustness of biochemical network models. *BMC Bioinformatics* **3**, 38 (2002).

[9] Ma, W., Lai, L., Ouyang, Q. & Tang, C. Robustness and modular design of the Drosophila segment polarity network. *Molecular systems biology* **2**, 70 (2006).

[10] Tsai, T. Y.-C. *et al.* Robust, tunable biological oscillations from interlinked positive and negative feedback loops. *Science (New York, NY)* **321**, 126–129 (2008).

[11] Ma, W., Trusina, A., El-Samad, H., Lim, W. A. & Tang, C. Defining network topologies that can achieve biochemical adaptation. *Cell* **138**, 760–773 (2009).

[12] Dayarian, A., Chaves, M., Sontag, E. D. & Sengupta, A. M. Shape, size, and robustness: feasible regions in the parameter space of biochemical networks. *PLoS Comput Biol* **5**, e1000256 (2009).

[13] Thomas, R. Boolean formalization of genetic control circuits. *Journal of Theoretical Biology* **42**, 563–585 (1973).

[14] Bornholdt, S. Boolean network models of cellular regulation: prospects and limitations. *J. Roy. Soc. Interface* **5**, S85–S94 (2008).

[15] Albert, R. Boolean Modeling of Genetic Regulatory Networks. *Lecture Notes is Physics* **650**, 459–481 (2004).

[16] Li, F., Long, T., Lu, Y., Ouyang, Q. & Tang, C. The yeast cell-cycle network is robustly designed. *Proc Natl Acad Sci U S A* **101**, 4781–4786 (2004).

[17] Faure, A., Naldi, A., Chaouiya, C. & Thieffry, D. Dynamical analysis of a generic Boolean model for the control of the mammalian cell cycle. *Bioinformatics* **22**, e124–31 (2006).

[18] Davidich, M. I. & Bornholdt, S. Boolean Network Model Predicts Cell Cycle Sequence of Fission Yeast. *PLoS ONE* **3**, e1672 (2008).

[19] Albert, R. & Othmer, H. G. The topology of the regulatory interactions predicts the expression pattern of the segment polarity genes in Drosophila melanogaster. *J Theor Biol* **223**, 1–18 (2003).

[20] Li, S., Assmann, S. M. & Albert, R. Predicting essential components of signal transduction networks: a dynamic model of guard cell abscisic acid signaling. *PLoS Biol* **4**, e312 (2006).

[21] Wang, R.-S. *et al.* Common and unique elements of the ABA-regulated transcriptome of Arabidopsis guard cells. *BMC Genomics* **12**, 216 (2011).

[22] Zhang, R. *et al.* Network model of survival signaling in large granular lymphocyte leukemia. *Proceedings of the National Academy of Sciences* **105**, 16308–16313 (2008).

[23] Schlatter, R. *et al.* ON/OFF and beyond–a boolean model of apoptosis. *PLoS Comput Biol* **5**, e1000595 (2009).

[24] Philippi, N. *et al.* Modeling system states in liver cells: survival, apoptosis and their modifications in response to viral infection. *BMC systems biology* **3**, 97 (2009).

[25] Mai, Z. & Liu, H. Boolean network-based analysis of the apoptosis network: irreversible apoptosis and stable surviving. *Journal of Theoretical Biology* **259**, 760–769 (2009).

[26] Alvarez-Buylla, E. R. *et al.* Floral morphogenesis: stochastic explorations of a gene network epigenetic landscape. *PLoS ONE* **3**, e3626 (2008).

[27] Flöttmann, M., Scharp, T. & Klipp, E. A Stochastic Model of Epigenetic Dynamics in Somatic Cell Reprogramming. *Frontiers in physiology* **3**, 216 (2012).

[28] Mortveit, H. & Reidys, C. *An Introduction to Sequential Dynamical Systems* (Springer Science & Business Media, 2007).

[29] Gire, V. & Dulic, V. Senescence from G2 arrest, revisited. *Cell Cycle* **14**, 297–304 (2015).

[30] Dong, P. *et al.* Division of labour between Myc and G1 cyclins in cell cycle commitment and pace control. *Nature Communications* **5**, 4750 (2014).

[31] Leone, G., DeGregori, J., Sears, R., Jakoi, L. & Nevins, J. R. Myc and Ras collaborate in inducing accumulation of active cyclin E/Cdk2 and E2F. *Nature* **387**, 422–426 (1997).

[32] Sears, R., Ohtani, K. & Nevins, J. R. Identification of positively and negatively acting elements regulating expression of the E2F2 gene in response to cell growth signals. *Molecular and cellular biology* **17**, 5227–5235 (1997).

[33] Batsché, E., Lipp, M. & Cremisi, C. Transcriptional repression and activation in the same cell type of the human c-MYC promoter by the retinoblastoma gene protein: antagonisation of both effects by SV40 T antigen. *Oncogene* **9**, 2235–2243 (1994).

[34] Oswald, F., Lovec, H., Möröy, T. & Lipp, M. E2F-dependent regulation of human MYC: trans-activation by cyclins D1 and A overrides tumour suppressor protein functions. *Oncogene* **9**, 2029–2036 (1994).

[35] Fan, J. & Bertino, J. R. Functional roles of E2F in cell cycle regulation. *Oncogene* **14**, 1191–1200 (1997).

[36] Sherr, C. J. & Roberts, J. M. Living with or without cyclins and cyclin-dependent kinases. *Genes & Development* **18**, 2699–2711 (2004).

[37] Krek, W. *et al.* Negative regulation of the growth-promoting transcription factor E2F-1 by a stably bound cyclin A-dependent protein kinase. *Cell* **78**, 161–172 (1994).

[38] Spencer, S. L. *et al.* The proliferation-quiescence decision is controlled by a bifurcation in CDK2 activity at mitotic exit. *Cell* **155**, 369–383 (2013).

[39] Overton, K. W., Spencer, S. L., Noderer, W. L., Meyer, T. & Wang, C. L. Basal p21 controls population heterogeneity in cycling and quiescent cell cycle states. *Proceedings of the National Academy of Sciences* 201409797 (2014).

[40] Lu, Z. & Hunter, T. Ubiquitylation and proteasomal degradation of the p21(Cip1), p27(Kip1) and p57(Kip2) CDK inhibitors. *Cell Cycle* **9**, 2342–2352 (2010).

[41] Stewart, Z. A., Leach, S. D. & Pietenpol, J. A. p21(Waf1/Cip1) inhibition of cyclin E/Cdk2 activity prevents endoreduplication after mitotic spindle disruption. *Molecular and cellular biology* **19**, 205–215 (1999).

[42] Mann, M. & Klemm, K. Efficient exploration of discrete energy landscapes. *Phys. Rev. E* **83**, 011113 (2011).

[43] Ravasz, E., Gnanakaran, S. & Toroczkai, Z. Network Structure of Protein Folding Pathways. *arXiv:0705.0912v1* (2007).

[44] FRUCHTERMAN, T. & REINGOLD, E. Graph Drawing by Force-Directed Placement. *Software - Practice and Experience* **21**, 1129–1164 (1991).

[45] Guo, Z.-y. *et al.* The elements of human cyclin d1 promoter and regulation involved. *Clinical epigenetics* **2**, 63–76 (2011).

[46] Daksis, J. I., Lu, R. Y., Facchini, L. M., Marhin, W. W. & Penn, L. Myc induces cyclin d1 expression in the absence of de novo protein synthesis and links mitogen-stimulated signal transduction to the cell cycle. *Oncogene* **9**, 3635–3645 (1994).

[47] Mateyak, M., Obaya, A. & Sedivy, J. c-Myc regulates cyclin D-Cdk4 and-Cdk6 activity but affects cell cycle progression at multiple independent points. *Molecular and cellular biology* **19**, 4672–4683 (1999).

[48] Matsumura, I., Tanaka, H. & Kanakura, Y. E2f1 and c-myc in cell growth and death. *Cell Cycle* **2**, 332–335 (2003).

[49] Leung, J., Ehmann, G., Giangrande, P. & Nevins, J. A role for myc in facilitating transcription activation by e2f1. *Oncogene* **27**, 4172–4179 (2008).

[50] Ohtani, K., Degregori, J. & Nevins, J. R. Regulation of the cyclin e gene by transcription factor e2f1. *Proceedings of the National Academy of Sciences* **92**, 12146–12150 (1995).

[51] Helin, K. Regulation of cell proliferation by the e2f transcription factors. *Current opinion in genetics & development* **8**, 28–35 (1998).

[52] Coqueret, O. New roles for p21 and p27 cell-cycle inhibitors: a function for each cell compartment? *Trends in cell biology* **13**, 65–70 (2003).

[53] Chellappan, S. P., Hiebert, S., Mudryj, M., Horowitz, J. M. & Nevins, J. R. The e2f transcription factor is a cellular target for the rb protein. *Cell* **65**, 1053–1061 (1991).

[54] Weintraub, S. J., Prater, C. A. & Dean, D. C. Retinoblastoma protein switches the E2F site from positive to negative element. *Nature* **358**, 259–261 (1992).

[55] Johnson, D. G., Ohtani, K. & Nevins, J. R. Autoregulatory control of e2f1 expression in response to positive and negative regulators of cell cycle progression. *Genes & Development* **8**, 1514–1525 (1994).

[56] Leone, G., DeGregori, J., Sears, R., Jakoi, L. & Nevins, J. R. Myc and ras collaborate in inducing accumulation of active cyclin e/cdk2 and e2f. *Nature* **387**, 422–426 (1997).

[57] Tanaka, H. *et al.* E2f1 and c-myc potentiate apoptosis through inhibition of nf-κb activity that facilitates mnsod-mediated ros elimination. *Molecular cell* **9**, 1017–1029 (2002).

[58] Thalmeier, K., Synovzik, H., Mertz, R., Winnacker, E. & Lipp, M. Nuclear factor e2f mediates basic transcription and trans-activation by e1a of the human myc promoter. *Genes & development* **3**, 527–536 (1989).

[59] Roussel, M., Davis, J., Cleveland, J., Ghysdael, J. & Hiebert, S. Dual control of myc expression through a single dna binding site targeted by ets family proteins and e2f-1. *Oncogene* **9**, 405–415 (1994).

[60] Müller, D. *et al.* Cdk2-dependent phosphorylation of p27 facilitates its Myc-induced release from cyclin E/cdk2 complexes. *Oncogene* **15**, 2561–2576 (1997).

[61] Sheaff, R. J., Groudine, M., Gordon, M., Roberts, J. M. & Clurman, B. E. Cyclin e-cdk2 is a regulator of p27kip1. *Genes & development* **11**, 1464–1478 (1997).

[62] Kato, J., Matsushime, H., Hiebert, S. W., Ewen, M. E. & Sherr, C. J. Direct binding of cyclin d to the retinoblastoma gene product (prb) and prb phosphorylation by the cyclin d-dependent kinase cdk4. *Genes and Development* **7**, 331–331 (1993).

[63] Ewen, M. E. *et al.* Functional interactions of the retinoblastoma protein with mammalian d-type cyclins. *Cell* **73**, 487–497 (1993).

[64] Novak, B. & Tyson, J. J. A model for restriction point control of the mammalian cell cycle. *Journal of Theoretical Biology* **230**, 563–579 (2004).

[65] Hinds, P. W. *et al.* Regulation of retinoblastoma protein functions by ectopic expression of human cyclins. *Cell* **70**, 993–1006 (1992).

[66] Manchado, E., Eguren, M. & Malumbres, M. The anaphase-promoting complex/cyclosome (APC/C): cell-cycle-dependent and -independent functions. *Biochem Soc Trans* **38**, 65–71 (2010).

[67] Peters, J.-M. The anaphase promoting complex/cyclosome: a machine designed to destroy. *Nature reviews Molecular cell biology* **7**, 644–656 (2006).

[68] Qiao, X., Zhang, L., Gamper, A. M., Fujita, T. & Wan, Y. APC/C-Cdh1: from cell cycle to cellular differentiation and genomic integrity. *Cell Cycle* **9**, 3904–3912 (2010).

[69] Reddy, S. K., Rape, M., Margansky, W. A. & Kirschner, M. W. Ubiquitination by the anaphase-promoting complex drives spindle checkpoint inactivation. *Nature* **446**, 921–925 (2007).

[70] Nezi, L. & Musacchio, A. Sister chromatid tension and the spindle assembly checkpoint. *Current opinion in cell biology* **21**, 785–795 (2009).

[71] Thornton, B. R. & Toczyski, D. P. Precise destruction: an emerging picture of the apc. *Genes & development* **20**, 3069–3078 (2006).

[72] Donzelli, M. *et al.* Dual mode of degradation of cdc25 a phosphatase. *The EMBO journal* **21**, 4875–4884 (2002).

[73] Hoffmann, I., Draetta, G. & Karsenti, E. Activation of the phosphatase activity of human cdc25A by a cdk2-cyclin E dependent phosphorylation at the G1/S transition. *EMBO J* **13**, 4302–4310 (1994).

[74] Frazer, C. & Young, P. G. Phosphorylation mediated regulation of Cdc25 activity, localization and stability. In Huang, C. (ed.) *Protein Phosphorylation in Human Health*, 395–436 (InTech, 2012).

[75] Karaïskou, A., Cayla, X., Haccard, O., Jessus, C. & Ozon, R. MPF amplification in Xenopus oocyte extracts depends on a two-step activation of cdc25 phosphatase. *Experimental Cell Research* **244**, 491–500 (1998).

[76] Hoffmann, I., Clarke, P., Marcote, M. J., Karsenti, E. & Draetta, G. Phosphorylation and activation of human cdc25-c by cdc2–cyclin b and its involvement in the self-amplification of mpf at mitosis. *The EMBO journal* **12**, 53 (1993).

[77] Harper, J. W. The anaphase-promoting complex: it's not just for mitosis any more. *Genes & Development* **16**, 2179–2206 (2002).

[78] Jackman, M. & Pines, J. Cyclins and the g2/m transition. *Cancer surveys* **29**, 47–73 (1996).

[79] Heald, R., McLoughlin, M. & McKeon, F. Human wee1 maintains mitotic timing by protecting the nucleus from cytoplasmically activated Cdc2 kinase. *Cell* **74**, 463–474 (1993).

[80] Deibler, R. W. & Kirschner, M. W. Quantitative reconstitution of mitotic CDK1 activation in somatic cell extracts. *Mol Cell* **37**, 753–767 (2010).

[81] Blomberg, I. & Hoffmann, I. Ectopic expression of cdc25a accelerates the g1/s transition and leads to premature activation of cyclin e-and cyclin a-dependent kinases. *Molecular and cellular biology* **19**, 6183–6194 (1999).

[82] Katsuno, Y. *et al.* Cyclin A-Cdk1 regulates the origin firing program in mammalian cells. *Proceedings of the National Academy of Sciences* **106**, 3184–3189 (2009).

[83] Yam, C. H., Fung, T. K. & Poon, R. Y. C. Cyclin A in cell cycle control and cancer. *Cellular and molecular life sciences : CMLS* **59**, 1317–1326 (2002).

[84] Rape, M. & Kirschner, M. W. Autonomous regulation of the anaphase-promoting complex couples mitosis to S-phase entry. *Nature* **432**, 588–595 (2004).

[85] He, E. *et al.* System-level feedbacks make the anaphase switch irreversible. *Proceedings of the National Academy of Sciences* **108**, 10016–10021 (2011).

[86] Chen, R.-H., Waters, J. C., Salmon, E. & Murray, A. W. Association of spindle assembly checkpoint component xmad2 with unattached kinetochores. *Science* **274**, 242–246 (1996).

[87] Musacchio, A. & Salmon, E. D. The spindle-assembly checkpoint in space and time. *Nature reviews Molecular cell biology* **8**, 379–393 (2007).

[88] Rudner, A. D. & Murray, A. W. Phosphorylation by cdc28 activates the cdc20-dependent activity of the anaphase-promoting complex. *The Journal of cell biology* **149**, 1377–1390 (2000).

[89] Stacey, D. W. Three Observations That Have Changed Our Understanding of Cyclin D1 and p27 in Cell Cycle Control. *Genes & cancer* **1**, 1189–1199 (2010).

[90] Diehl, J. A., Zindy, F. & Sherr, C. J. Inhibition of cyclin D1 phosphorylation on threonine-286 prevents its rapid degradation via the ubiquitin-proteasome pathway. *Genes & Development* **11**, 957–972 (1997).

[91] Hitomi, M. & Stacey, D. W. Cyclin d1 production in cycling cells depends on ras in a cell-cycle-specific manner. *Current biology* **9**, 1075–S2 (1999).

[92] Aktas, H., Cai, H. & Cooper, G. M. Ras links growth factor signaling to the cell cycle machinery via regulation of cyclin d1 and the cdk inhibitor p27kip1. *Molecular and cellular biology* **17**, 3850–3857 (1997).

[93] Harper, J. W. *et al.* Inhibition of cyclin-dependent kinases by p21. *Molecular biology of the cell* **6**, 387–400 (1995).

[94] Xiong, Y. *et al.* p21 is a universal inhibitor of cyclin kinases. *nature* **366**, 701–704 (1993).

[95] Lunn, C. L., Chrivia, J. C. & Baldassare, J. J. Activation of Cdk2/Cyclin E complexes is dependent on the origin of replication licensing factor Cdc6 in mammalian cells. *Cell Cycle* **9**, 4533–4541 (2010).

[96] Xu, M., Sheppard, K. A., Peng, C. Y., Yee, A. S. & Piwnica-Worms, H. Cyclin A/CDK2 binds directly to E2F-1 and inhibits the DNA-binding activity of E2F-1/DP-1 by phosphorylation. *Molecular and cellular biology* **14**, 8420–8431 (1994).

[97] Kelly, K., Cochran, B. H., Stiles, C. D. & Leder, P. Cell-specific regulation of the c-myc gene by lymphocyte mitogens and platelet-derived growth factor. *Cell* **35**, 603–610 (1983).

[98] Zhu, J., Blenis, J. & Yuan, J. Activation of PI3K/Akt and MAPK pathways regulates Myc-mediated transcription by phosphorylating and promoting the degradation of Mad1. *Proceedings of the National Academy of Sciences* **105**, 6584–6589 (2008).

[99] Montagnoli, A. *et al.* Ubiquitination of p27 is regulated by cdk-dependent phosphorylation and trimeric complex formation. *Genes & development* **13**, 1181–1189 (1999).

[100] Ishida, N. *et al.* Phosphorylation of p27 kip1 on serine 10 is required for its binding to crm1 and nuclear export. *Journal of Biological Chemistry* **277**, 14355–14358 (2002).

[101] Coqueret, O. New roles for p21 and p27 cell-cycle inhibitors: a function for each cell compartment? *Trends in cell biology* **13**, 65–70 (2003).

[102] Harbour, J. W., Luo, R. X., Dei Santi, A., Postigo, A. A. & Dean, D. C. Cdk phosphorylation triggers sequential intramolecular interactions that progressively block rb functions as cells move through g1. *Cell* **98**, 859–869 (1999).

[103] Taya, Y. Rb kinases and rb-binding proteins: new points of view. *Trends in biochemical sciences* **22**, 14–17 (1997).

[104] Chen, X. & Prywes, R. Serum-induced expression of the cdc25agene by relief of e2f-mediated repression. *Molecular and cellular biology* **19**, 4695–4702 (1999).

[105] Sørensen, C. S. *et al.* Chk1 regulates the S phase checkpoint by coupling the physiological turnover and ionizing radiation-induced accelerated proteolysis of Cdc25A. *Cancer cell* **3**, 247–258 (2003).

[106] Lee, J., Kumagai, A. & Dunphy, W. G. Positive regulation of wee1 by chk1 and 14-3-3 proteins. *Molecular biology of the cell* **12**, 551–563 (2001).

[107] Coverley, D., Laman, H. & Laskey, R. A. Distinct roles for cyclins e and a during dna replication complex assembly and activation. *Nature Cell Biology* **4**, 523–528 (2002).

[108] Mailand, N. *et al.* Rapid destruction of human cdc25a in response to dna damage. *Science* **288**, 1425–1429 (2000).

[109] Donzelli, M. & Draetta, G. F. Regulating mammalian checkpoints through Cdc25 inactivation. *EMBO reports* **4**, 671–677 (2003).
